# TRAT1 overexpression delays cancer progression and is associated with immune infiltration in lung adenocarcinoma

**DOI:** 10.3389/fonc.2022.960866

**Published:** 2022-10-06

**Authors:** Xiao-Yue Xiao, Qiang Guo, Song Tong, Chuang-Yan Wu, Jiu-Ling Chen, Yu Ding, Jun-Hao Wan, Shan-Shan Chen, Si-Hua Wang

**Affiliations:** ^1^ Department of Cardiovascular Surgery, Union Hospital, Tongji Medical College, Huazhong University of Science and Technology, Wuhan, China; ^2^ Department of Thoracic Surgery, Union Hospital, Tongji Medical College, Huazhong University of Science and Technology, Wuhan, China; ^3^ Key Laboratory for Molecular Diagnosis of Hubei Province, The Central Hospital of Wuhan, Tongji Medical College, Huazhong University of Science and Technology, Wuhan, China

**Keywords:** T cell receptor associated transmembrane adaptor 1, lung adenocarcinoma, T-cell receptor, immune microenvironment, RNA modification

## Abstract

The roles and mechanisms of T-cell receptor (TCR)-associated transmembrane adaptor 1 (TRAT1) in lung adenocarcinoma (LAC) have not yet been reported in the relevant literature. Therefore, this study aimed to understand the roles and mechanisms of TRAT1 in LAC using bioinformatics and *in vitro* experiments. TRAT1 expression levels in LAC samples were analysed using various databases. TRAT1 co-expressed genes were acquired by the correlation analysis of LAC tissues. The functional mechanisms and protein network of TRAT1 co-expressed genes were analysed using bioinformatics analysis. The expression of TRAT1 was activated in LAC cells, and the roles of TRAT1 overexpression in the growth and migration of cancer cells was investigated using flow cytometry, Cell Counting Kit-8 (CCK-8), and migration and invasion assays. The relationship between TRAT1 overexpression, the immune microenvironment, and RNA modification was evaluated using correlation analysis. TRAT1 expression levels were significantly abnormal at multiple mutation sites and were related to the prognosis of LAC. TRAT1 co-expressed genes were involved in cell proliferation, adhesion, and differentiation, and TRAT1 overexpression significantly inhibited cell viability, migration, and invasion and promoted apoptosis of A549 and H1299 cells, which might be related to the TCR, B cell receptor (BCR), MAPK, and other pathways. TRAT1 expression levels were significantly correlated with the ESTIMATE, immune, and stromal scores in the LAC microenvironment. Additionally, TRAT1 expression levels were significantly correlated with the populations of B cells, CD8 T cells, cytotoxic cells, and other immune cells. TRAT1 overexpression was significantly correlated with the expression of immune cell markers (such as *PDCD1, CD2, CD3E*) and genes involved in RNA modification (such as *ALKBH1*, *ALKBH3*, *ALKBH5*). In conclusions, TRAT1 overexpression inhibited the growth and migration of LAC cells, thereby delaying cancer progression, and was correlated with the LAC microenvironment and RNA modifications.

## Introduction

Lung adenocarcinoma (LAC) is a common subtype of non-small cell lung cancer (NSCLC) (1–3). At present, most patients with early LAC achieve better survival after surgical treatment (4). However, the long-term prognosis of patients with advanced LAC after surgical treatment remains poor. Some genes, microRNAs (miRNAs), and long noncoding RNAs (lncRNAs) play crucial roles in the occurrence of LAC (5–9). For example, the lncRNA LINC00319 is overexpressed in LAC tissues and cells. However, the prognosis of patients with LAC showing significantly elevated expression levels of LINC00319 is dismal. Inhibition of LINC00319 expression inhibits the growth of LAC cells *in vitro*. LINC00319 promotes LAC progression by regulating the miR-450b-5p/EZH2 signalling pathway (6). Additionally, the lncRNA RAET1K is significantly upregulated in LAC tissues and correlates with poor prognosis in patients with LAC. RAET1K affects cell cycle, causing G1/S arrest *via* the miR-135a-5p/CCNE1 signalling mechanism and subsequently participates in the regulation of LAC progression (7). Similarly, RHPN2 is also overexpressed in the LAC tissues. High expression levels of RHPN2 are associated with a poor prognosis in patients with LAC and promote LAC cell growth and migration (9).

T cell receptor (TCR)-associated transmembrane adaptor 1 (TRAT1) is one of the hub regulatory genes of TCR and is associated with cancer progression (10–12). For example, IL-4 can trigger IL-17-producing CD8 T cell (Tc17) cytotoxicity and induce cell proliferation. IL-4/AKT signalling drives the upregulation of TRAT1 expression in Tc17 cells to stabilize the TCR and enhance Tc17 cytotoxicity (11). A risk model based on five immune response-related genes, *TRAT1*, *IL2RB*, *CTLA4*, *IGHM*, and *IL21R*, can predict the prognosis of patients with breast cancer (12). Our previous results published in the journal of Current Medical Science showed that the expression level of TRAT1 was significantly decreased in NSCLC, LAC, and lung squamous cell carcinoma (LUSC). Decreased expression levels of TRAT1 are associated with poor prognosis in patients with NSCLC, LAC, and LUSC and are associated with smoking, clinical stage, histological subtype, and lymph node metastasis in patients with LAC. Based on these findings, we aimed to explore the roles and mechanisms of TRAT1 in the progression of LAC using a comprehensive analysis in this study. Activation of TRAT1 expression in LAC cells resulted in the identification of the effects of TRAT1 overexpression on LAC cell proliferation, migration, and invasion, and the relationship between TRAT1 overexpression, LAC immune microenvironment, and RNA modifications was investigated to assess the role of TRAT1 in LAC progression.

## Materials and methods

### cBioPortal database

The cBioPortal (http://www.cbioportal.org/index.do) database contains cancer- and immune-related data from multiple research centres. Data on LAC tissues obtained from the cBioPortal database was used to explore the relationship between TRAT1 expression, LAC mutation sites, and the clinicopathological characteristics of patients with LAC. Additionally, the relationship between TRAT1 expression and LAC immunity was explored using data from the MSK LAC cellular immunity.

### Identification of TRAT1 expression at mutation sites in patients with LAC

The muTarget (https://www.mutarget.com/) database is used for cancer biomarker discovery. In the muTarget database, we used the following screening parameters: target-TRAT1, cancer type-LAC, and mutation rate >1%, to explore the expression levels of TRAT1 at the mutation sites of patients with LAC.

### Biological functions, mechanisms, and protein-protein interaction (PPI) network of TRAT1 strongly co-expressed genes

TRAT1 co-expressed genes in 535 LAC tissues from The Cancer Genome Atlas (TCGA) database were identified using a correlation analysis. Genes were identified to be strongly co-expressed when the correlation coefficient r > 0.4 or < −0.4 and P < 0.001, as described in the literature (13, 14). The biological process, molecular function, and cellular composition of TRAT1 strongly co-expressed genes were explored using the gene ontology (GO) annotation and adjusted P < 0.05 as the standard of significance. The signalling mechanisms associated with the strongly co-expressed genes of TRAT1 were investigated using the Kyoto Encyclopedia of Genes and Genomes (KEGG) analysis (14). The PPI network of TRAT1 strongly co-expressed genes was constructed using the STRING database and further visualized using Cytoscape software.

### Cell culture and construction of TRAT1 overexpression cell lines

A549 and H1299 cell lines were obtained from the Shanghai Institute of Biochemistry and Cell Biology (Shanghai, China) and were cultured in Dulbecco’s modified Eagle’s medium (DMEM) (Thermo Fisher Scientific, USA) supplemented with 10% foetal bovine serum (FBS) (Thermo Fisher Scientific, USA) at 37°C in an incubator with 5% CO_2_. The pCDNA3.1(-) TRAT1 expression vector and pCDNA3.1(-) control vector were synthesized by Tianyi Huiyuan (China). A549 and H1299 cells were cultured in 6-well plates at a suitable density for 24 h in an incubator and then transfected with TRAT1 overexpression and control vectors using Lipofectamine 3000 (Thermo Fisher Scientific, USA) for 24 h. TRAT1 overexpression was confirmed in the established cell models of LAC by RT-PCR and western blotting.

### Identification of cell models of LAC *via* the RT-PCR

TRIzol was used to isolate total RNA from LAC cells, and total RNA was reverse transcribed into cDNA using the Revertra Ace qPCR RT Kit (Toyobo Life Science, Japan). UltraSYBR mixture (CWBio) was used to perform the PCR cycles on ABI Stepone Plus (Thermo Fisher Scientific, USA). Changes in the relative TRAT1 mRNA expression levels were calculated by the 2^^ΔΔct^ method. The primer sequences used for TRAT1 and β-actin were as follows: TRAT1 forward: 5′-ACAAATGAAAGCCCGACCAG-3′ and reverse: 5′-ATCAAGTGAGGCGTAGCACA-3′; β-actin forward 5’-ACTCTTCCAGCCTTCCTTCC-3’ and reverse 5’-CGTCATACTCCTGCTTGCTG-3’.

### Western blotting

In well-grown transfected A549 and H1299 cells, each group of A549 and H1299 cells were lysed using a lysis buffer (Beyotime, China) and protein quantification was performed by bicinchoninic acid (BCA) method (Beyotime, China). Total protein of 30 µg was used for protein gel electrophoresis, followed by TRAT1 antibody (1:1000) (Univ, China) incubation and secondary antibody (Univ, China) (1:5000) incubation. These steps were followed by membrane washing and exposure to determine TRAT1 protein expression.

### Detection of proliferation and apoptosis of LAC cells

Cells were plated in 96-well plates at a density of 5 × 10^3^ cells/well. Following 24 h of incubation, the cells were transfected with pCDNA3.1(-) TRAT1 expression vector and pCDNA3.1(-) control vector for 24, 48, and 72 h, followed by the addition of 10 μl Cell Counting Kit-8 (CCK-8) solution to each well. Absorbance was measured at 450 nm using an EnSpire multimode plate reader, and the optical density (OD) was calculated for the cell viability assay. Cell apoptosis was analysed using the phycoerythrin (PE) Annexin V Apoptosis Detection Kit І (BD Biosciences). Cells were seeded in 6-well plates, transfected for 24 h, and stained according to the manufacturer’s instructions. Apoptotic cells were immediately detected using the FACS Caliber II Sorter and the Cell Quest FACS system (BD Biosciences, USA). Data were analysed using the FlowJo software (version 7.6.5).

### Detection of migration and invasion of LAC cells

The cells were cultured in 12-well plates. A clean pipette tip was used to inflict a ‘wound’ when cells formed a confluent monolayer, and the cells cultured in DMEM were supplemented with 2% FBS. The cells were then transfected and images of the wound margins were captured using an optical light microscope (Olympus Corporation, Japan) at 0 h time point. Following incubation for 24 h, images of the same region of cells were captured for measurement. The wound healing rate was calculated using the following formula: The cells were subjected to a Transwell assay (Transwell system; Corning, USA) after transfection. The basolateral chamber was balanced with 600 ml of DMEM supplemented with 10% FBS, and 200 ml of A549 and H1299 cell suspensions were added to the apical chamber in FBS-free DMEM. After culturing the cells for 24 h at 37°C with 5% CO_2_, the transwell chambers were fixed with 5% paraformaldehyde for 15 min and then stained with 0.1% crystal violet for 10 min according to the manufacturer’s protocol. Transmembrane cells were observed under an inverted fluorescence microscope.

### Identification of the relationship between TRAT1 expression and immune microenvironment

The ESTIMATE, immune and stromal scores, and the populations of immune-infiltrating cells in 535 LAC tissues were calculated using single sample gene set enrichment analysis (ssGSEA) and an ESTIMATE algorithm. Correlation analysis was used to explore the relationship between TRAT1 expression and the ESTIMATE, immune and stromal scores, and immune-infiltrating cells. The ESTIMATE, immune and stromal scores, and the populations of immune infiltrating cells in the high- and low-TRAT1 expression groups were identified and grouped based on the median value of TRAT1 expression. In the Kaplan–Meier (K–M) plotter (http://kmplot.com/analysis/) database, the relationship between high- and low-TRAT1 expression levels and the prognosis-related immune cells of patients with LAC was explored using the K–M survival analysis and grouped by the median value of TRAT1 expression.

### Identification of the relationship between TRAT1 expression and immune cell markers and RNA modifications

The expression data of immune cell markers in the LAC tissues were extracted (15). Correlation analysis was used to explore the relationship between TRAT1 expression and the levels of immune-infiltrating cell markers in LAC tissues. The expression levels of LAC immune-infiltrating cell markers in the high- and low-TRAT1 expression groups were evaluated based on the median value of TRAT1 expression. Additionally, the relationship between TRAT1 expression levels and N6-methyladenosine (m6A)-, N1-methyladenosine (m1A)-, and 5-methylcytosine (m5C)-regulated genes was evaluated using correlation analysis.

### Statistical analysis

The expression levels of TRAT1 in the LAC cell models were analysed using the t-test. The relationship between the expression levels of TRAT1 and the immune microenvironment was analysed using a correlation analysis. Statistical significance was set at P < 0.05.

## Results

### Clinical values of TRAT1 expression level in LAC

**Figure 1:**
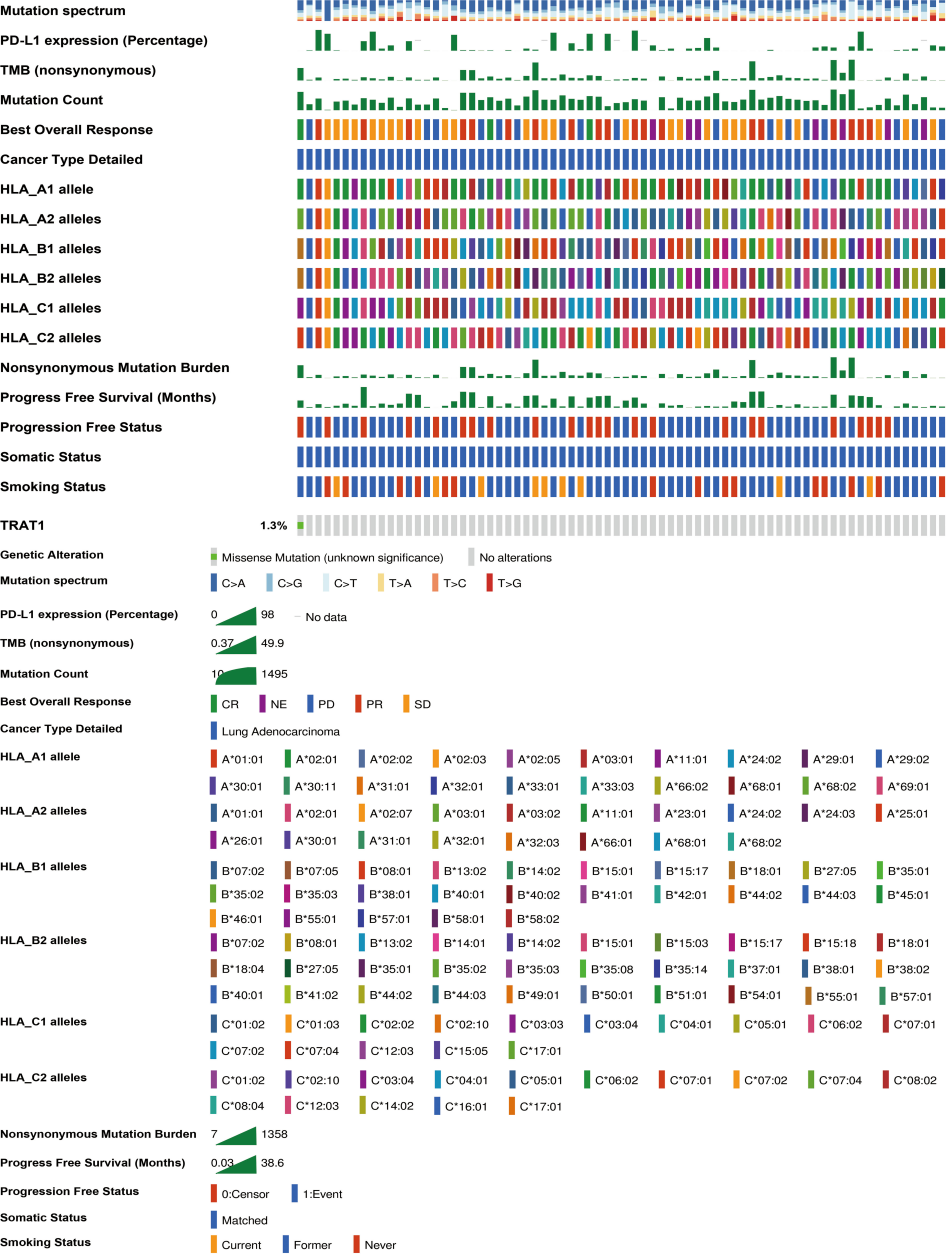
The relationship between TRAT1 expression levels and immune characteristics of LAC cells. LAC, lung adenocarcinoma.

**Figure 2:**
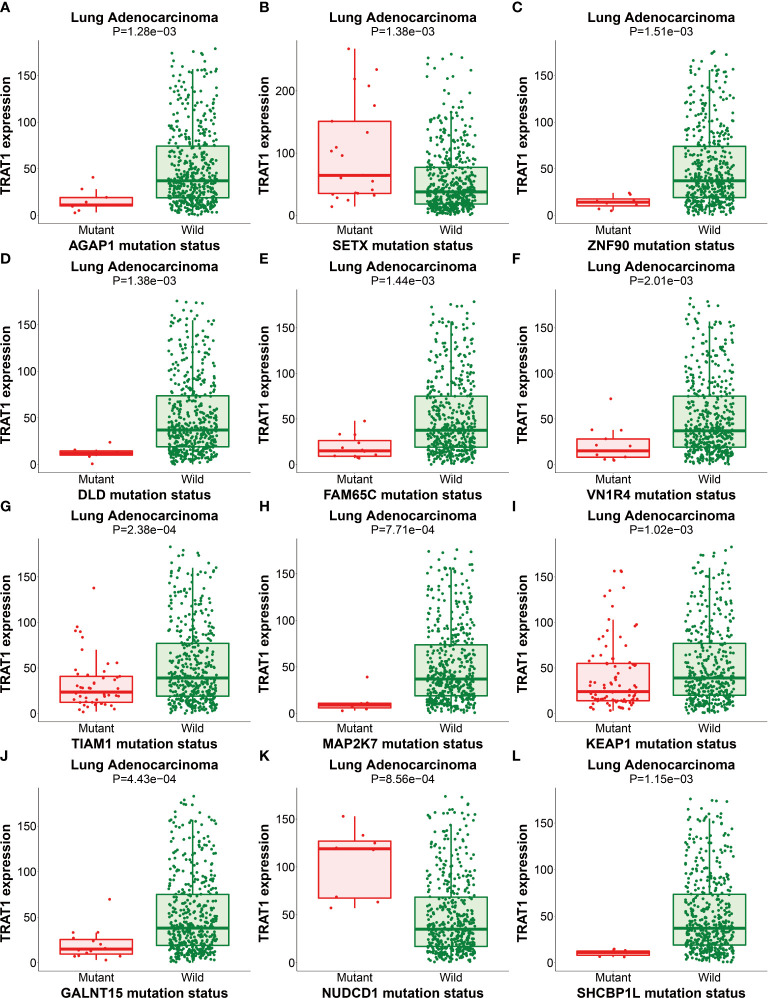
The expression of TRAT1 in LAC mutation sites. **(A)** *AGAP1*; **(B)** *SETX*; **(C)** *ZNF90*; **(D)** *DLD*; **(E)** *FAM65C*; **(F)** *VN1R4*; **(G)** *TIAM1*; **(H)** *MAP2K7*; **(I)** *KEAP1*; **(J)** *GALNT15*; **(K)** *NUDCD1*; **(L)** *SHCBP1*. LAC, lung adenocarcinoma.

We previously found that TRAT1 expression was significantly decreased, which was associated with dismal prognosis in NSCLC. In this study, the TRAT1 expression was related to overall survival (OS), progression-free survival (PFS), smoking history, forced expiratory volume in 1 s (FEV1), and other clinical characteristics of patients with LAC in the cBioPortal database (**Figure S1**). The relationship between TRAT1 expression levels and PD-L1 expression, tumour mutational burden (TMB), response efficacy, and other immune characteristics in LAC cells was also obtained from the cBioPortal database (**Figure 1**). In the muTarget database, TRAT1 expression levels were significantly elevated at *NUDCD1*, *SETX*, *CUL4A*, and other gene mutation sites, and were significantly decreased at *TIAM1*, *GALNT15*, *MAP2K7*, and other gene mutation sites (**Table 1** and **Figure 2**), which preliminarily suggested that TRAT1 has an important prognostic role and might be a prognostic biomarker in patients with LAC.

**Table 1:** Expression of TRAT1 in LAC mutation sites.

Sites	FC (M/W)	Direction	p	Sites	FC (M/W)	Direction	p
TIAM1	2	down	2.38E-04	UACA	2.5	down	5.33E-03
GALNT15	3.12	down	4.43E-04	SSTR1	2.22	down	5.46E-03
MAP2K7	5	down	7.71E-04	FOXO4	3.45	down	5.70E-03
NUDCD1	2.13	up	8.56E-04	SYT10	1.65	up	5.78E-03
KEAP1	1.52	down	1.02E-03	COL6A2	1.89	down	5.94E-03
SHCBP1L	5.88	down	1.15E-03	PDE6A	2.27	down	6.00E-03
AGAP1	4	down	1.28E-03	DIS3	3.03	down	6.34E-03
DLD	5	down	1.38E-03	ARHGAP4	2.38	down	6.52E-03
SETX	1.96	up	1.38E-03	MEIS1	2.94	down	6.68E-03
FAM65C	3.23	down	1.44E-03	CHD7	2	down	6.94E-03
ZNF90	4.35	down	1.51E-03	CEACAM20	4	down	7.32E-03
VN1R4	2.86	down	2.01E-03	AHR	1.96	up	7.39E-03
BMPER	2.17	down	2.65E-03	NNT	2.56	down	7.41E-03
LRRK1	1.69	down	2.80E-03	OR8D1	3.03	down	7.42E-03
CYP2B6	4.17	down	3.03E-03	LCE2B	3.7	down	7.51E-03
ZNF559-ZNF177	2.08	up	3.14E-03	SYT16	2.5	down	7.58E-03
COL28A1	2.44	down	3.22E-03	LAMA4	1.47	down	7.73E-03
XPC	4	down	3.46E-03	CARD8	1.97	up	8.09E-03
MYH15	2.27	down	3.47E-03	SIX3	3.33	down	8.19E-03
CUL4A	2.08	up	3.63E-03	WNT10B	3.33	down	8.19E-03
ANKRD34A	3.57	down	3.69E-03	OR5H15	2.63	down	8.53E-03
HELZ2	2.86	down	3.77E-03	KRT19	4	down	8.61E-03
CCDC141	1.95	up	4.30E-03	CP	3.85	down	8.71E-03
EFCAB5	2.21	up	4.36E-03	PCDH20	2.33	down	9.04E-03
MANEA	1.88	up	4.41E-03	ZNF181	2.34	up	9.04E-03
MTBP	3.7	down	4.57E-03	ERC1	2.17	down	9.21E-03
ZBTB33	3.57	down	4.73E-03	FMR1	2.38	down	9.43E-03
NPHS1	2.17	down	4.81E-03	TGFB2	2.34	up	9.73E-03
FGF6	3.85	down	5.17E-03	SLC36A4	2.08	up	9.77E-03
GCC2	2.13	down	5.26E-03	PC	3.03	down	9.85E-03

LAC, lung adenocarcinoma; M, mutant; W, wild; FC, fold change.

### Roles, mechanisms, and PPI network involved in the strongly co-expressed genes of TRAT1

**Figure 3:**
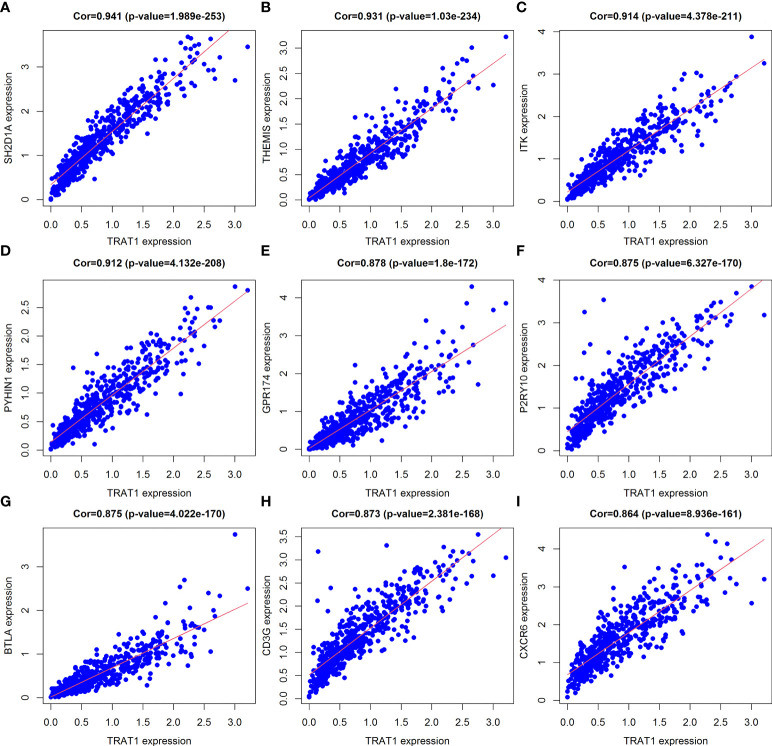
Top 9 TRAT1 co-expressed genes. **(A)** *SH2D1A*; **(B)** *THEMIS*; **(C)** *ITK*; **(D)** *PYHIN1*; **(E)** *GPR174*; **(F)** *P2RY10*; **(G)** *BTLA*; **(H)** *CD3G*; **(I)** *CXCR6*.

**Figure 4:**
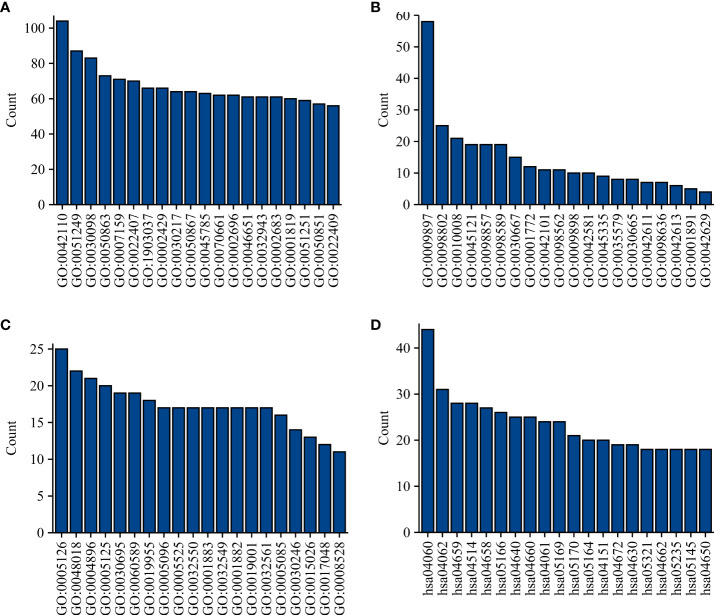
Functions and mechanisms of TRAT1 co-expressed genes. **(A)** BP; **(B)** CC; **(C)** MF; **(D)** Signalling pathways. BP, biological process; MF, molecular function; CC, cell component.

**Figure 5:**
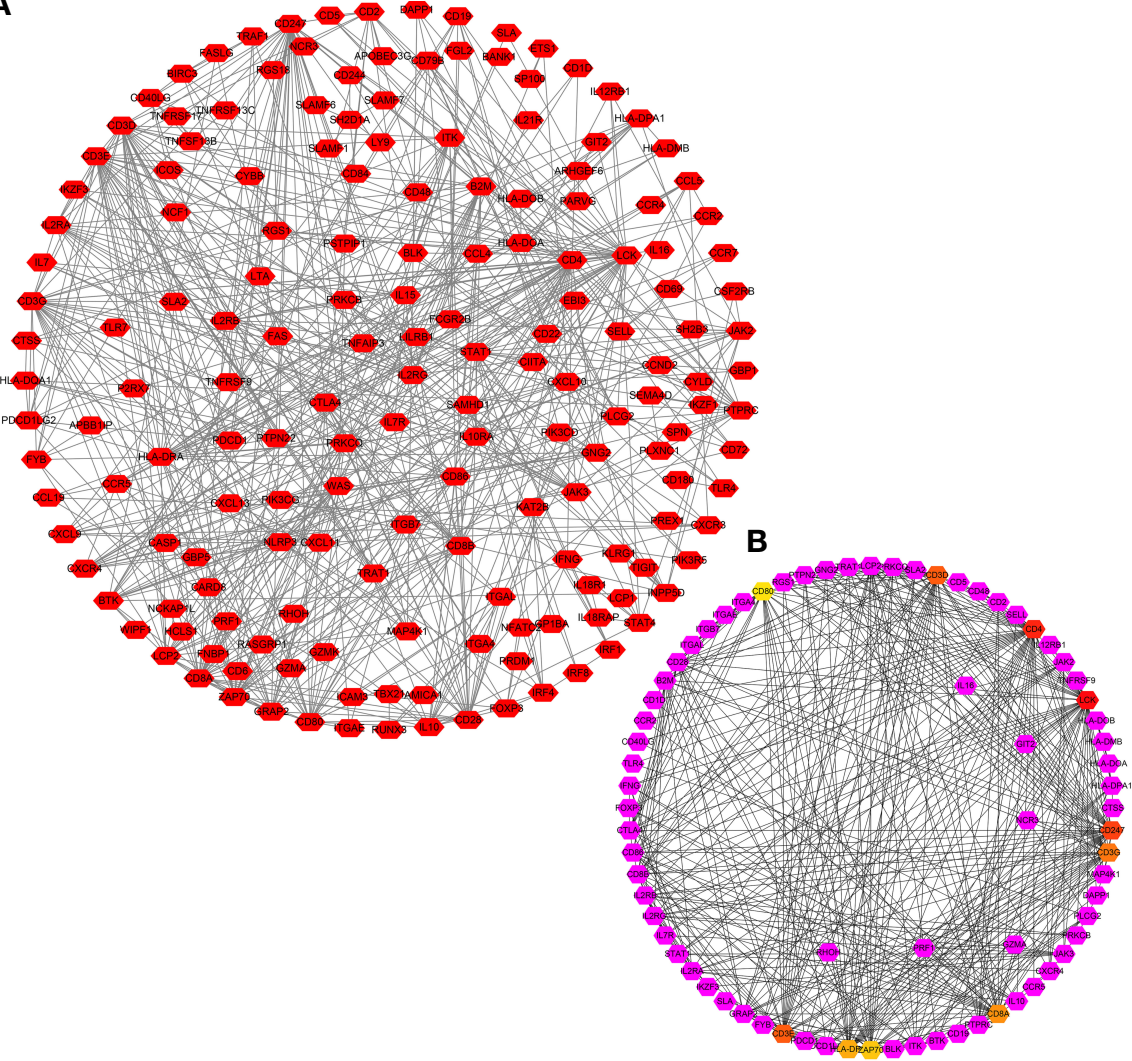
PPI network of TRAT1 co-expressed genes. **(A)** PPI network; **(B)** Hub genes in network. PPI, protein-protein interaction.

There were 806 strongly co-expressed genes of TRAT1 (**Table S1** and **Figure 3**). TRAT1 co-expressed genes were found to be involved in T cell activation, lymphocyte differentiation, T cell differentiation, lymphocyte proliferation, mononuclear cell proliferation, and others (**Figures 4A-C** and **Table S2**). In addition, TRAT1 co-expressed genes were involved in Th1, Th2, and Th17 cell differentiation; PD-L1 expression and PD-1 checkpoint pathway in cancer; TCR, B cell receptor (BCR), NF-kappa B, JAK-STAT, PI3K-AKT, VEGF, and other signalling pathways as per KEGG analysis (**Figure 4D** and **Table 2**). **Figure 5** shows the PPI network between the strongly co-expressed genes of TRAT1. Abnormal expression of TRAT1 is associated with cancer mechanisms and immunity, indicating that TRAT1 has an important role in LAC progression.

**Table 2:** Signalling pathways associated with strongly co-expressed genes of TRAT1.

ID	Description	P
hsa04658	Th1 and Th2 cell differentiation	7.81422E-21
hsa04060	Cytokine-cytokine receptor interaction	1.41871E-20
hsa04659	Th17 cell differentiation	4.51022E-20
hsa04640	Hematopoietic cell lineage	1.29929E-17
hsa04672	Intestinal immune network for IgA production	1.41944E-17
hsa05340	Primary immunodeficiency	4.00356E-17
hsa04660	T cell receptor signaling pathway	4.68872E-17
hsa04061	Viral protein interaction with cytokine and cytokine receptor	2.13951E-16
hsa04514	Cell adhesion molecules	5.56017E-16
hsa04062	Chemokine signaling pathway	1.19763E-15
hsa05330	Allograft rejection	2.97102E-14
hsa05321	Inflammatory bowel disease	9.45343E-14
hsa04940	Type I diabetes mellitus	4.90446E-12
hsa04662	B cell receptor signaling pathway	7.2681E-12
hsa05320	Autoimmune thyroid disease	8.25255E-12
hsa05235	PD-L1 expression and PD-1 checkpoint pathway in cancer	3.16225E-11
hsa05332	Graft-versus-host disease	6.01992E-11
hsa05140	Leishmaniasis	2.67387E-10
hsa05166	Human T-cell leukemia virus 1 infection	3.62424E-10
hsa05145	Toxoplasmosis	1.66618E-09
hsa05169	Epstein-Barr virus infection	1.78122E-09
hsa04064	NF-kappa B signaling pathway	3.67919E-09
hsa05323	Rheumatoid arthritis	4.99423E-09
hsa04650	Natural killer cell mediated cytotoxicity	2.1676E-08
hsa05162	Measles	5.55332E-08
hsa05164	Influenza A	5.65974E-08
hsa05416	Viral myocarditis	7.72787E-08
hsa04630	JAK-STAT signaling pathway	1.18029E-07
hsa04612	Antigen processing and presentation	2.12056E-07
hsa05135	Yersinia infection	2.47375E-07
hsa05170	Human immunodeficiency virus 1 infection	4.57741E-07
hsa04380	Osteoclast differentiation	5.10574E-07
hsa05310	Asthma	1.55445E-06
hsa05152	Tuberculosis	2.7408E-06
hsa04620	Toll-like receptor signaling pathway	6.15948E-06
hsa05167	Kaposi sarcoma-associated herpesvirus infection	2.81945E-05
hsa04217	Necroptosis	3.80142E-05
hsa05150	Staphylococcus aureus infection	7.31499E-05
hsa05142	Chagas disease	0.000127458
hsa04668	TNF signaling pathway	0.00029351
hsa05322	Systemic lupus erythematosus	0.000425906
hsa05144	Malaria	0.00045068
hsa04621	NOD-like receptor signaling pathway	0.000557478
hsa04625	C-type lectin receptor signaling pathway	0.000653286
hsa04611	Platelet activation	0.000704231
hsa04670	Leukocyte transendothelial migration	0.001334521
hsa04151	PI3K-Akt signaling pathway	0.002239946
hsa05143	African trypanosomiasis	0.00351225
hsa04145	Phagosome	0.003629697
hsa05163	Human cytomegalovirus infection	0.004372263
hsa04666	Fc gamma R-mediated phagocytosis	0.005808208
hsa04014	Ras signaling pathway	0.013684596
hsa05161	Hepatitis B	0.015824107
hsa05133	Pertussis	0.019672919
hsa05146	Amoebiasis	0.024475814
hsa04370	VEGF signaling pathway	0.024563808

### Elevated TRAT1 expression inhibited LAC cell growth and metastasis

**Figure 6:**
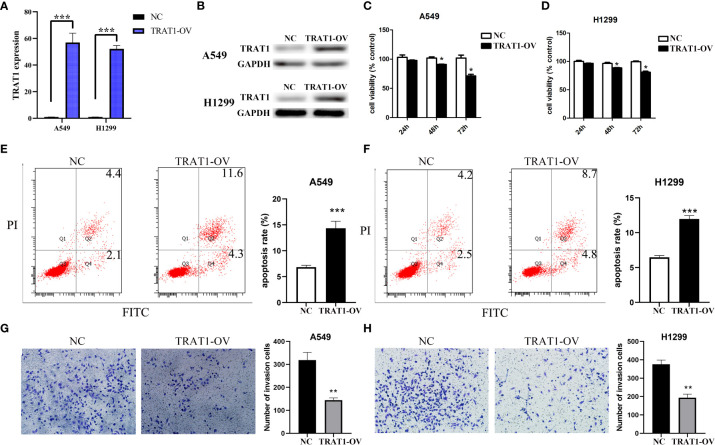
TRAT1 overexpression inhibited the cell growth and immune invasion in LAC cell models. **(A, B)** Construction of A549 and H1299 cell models of TRAT1 overexpression using the PCR and Western Blotting; **(C-F)** Decreased cell viability and increased apoptosis in TRAT1 overexpression group determined using the CCK-8 assay and flow cytometry; **(G, H)** Decreased cell invasive ability in TRAT1 overexpression group determined using the transwell experiment. LAC, lung adenocarcinoma; CCK-8, Cell Counting Kit-8; *P < 0.05; **P < 0.01; ***P < 0.001.

**Figure 7:**
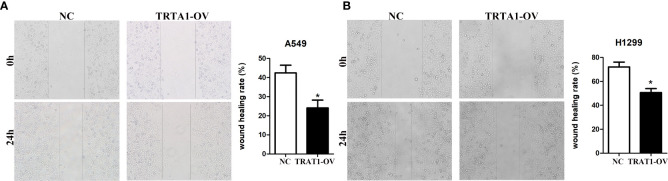
Elevated TRAT1 expression inhibited cell migration in LAC A549 and H1299 cells. **(A)** A549 cells; **(B)** H1299 cells. LAC, lung adenocarcinoma; *P < 0.05.

Cell models of TRAT1 overexpression were successfully established using the A549 and H1299 cells and confirmed by PCR and western-blotting (**Figures 6A, B**). The CCK-8 assay results showed that compared with the control group, the viability of A549 and H1299 cells in the TRAT1 overexpression group significantly decreased, and the cell viability of the two groups at 48 and 72 h was statistically significant (**Figures 6C, D**). TRAT1 overexpression significantly promoted apoptosis in the A549 and H1299 cells (**Figures 6E, F**). The results of the invasion experiment showed that increased expression of TRAT1 significantly inhibited the invasion of A549 and H1299 cells (**Figures 6G, H**). Additionally, the results of the scratch assay showed that elevated TRAT1 expression significantly inhibited the migration of A549 and H1299 cells with significant differences between the two groups (**Figures 7A, B**). This suggests that TRAT1 functions as a tumour suppressor in LAC progression to inhibit cancer growth and migration.

### TRAT1 was significantly associated with the LAC immune microenvironment

**Figure 8:**
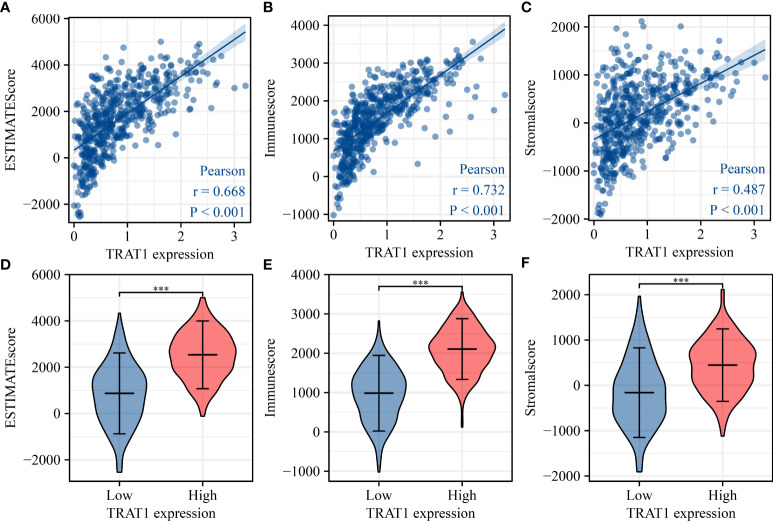
TRAT1 expression was significantly correlated with the levels of stromal, immune, and ESTIMATE scores in LAC tissues. **(A)** Stromal score; **(B)** Immune score; **(C)** ESTIMATE score; **(D–F)** The levels of stromal, immune, and ESTIMATE scores in high- and low-TRAT1 expression groups. LAC, lung adenocarcinoma.

**Figure 9:**
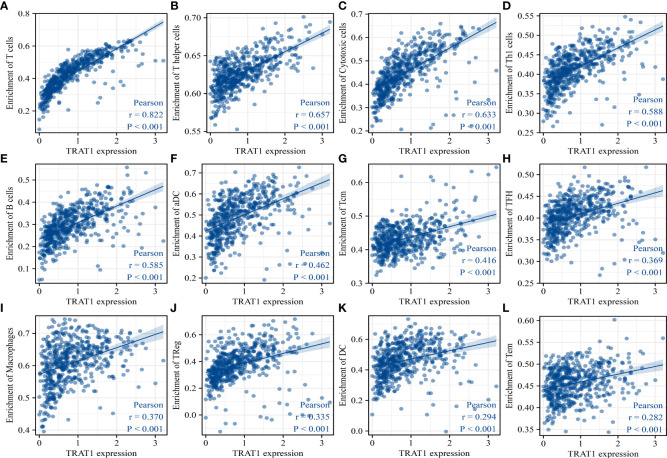
TRAT1 expression was significantly correlated with the populations of immune cells in LAC tissues. **(A)** T cells; **(B)** T helper cells; **(C)** Cytotoxic cells; **(D)** Th1 cells; **(E)** B cells; **(F)** aDC; **(G)** TCM; **(H)** TFH; **(I)** macrophages; **(J)** Treg; **(K)** DC; **(L)** TEM. LAC, lung adenocarcinoma; aDC, activated dendritic cells; TCM, central memory T cells; TFH, follicular helper T cells; Treg, regulatory T cells; TEM, effector memory T cells.

TRAT1 expression level was significantly correlated with the stromal (r = 0.487), immune (r = 0.732), and ESTIMATE scores (r = 0.668) (**Figures 8A-C**). TRAT1 expression was also significantly correlated with the populations of activated dendritic cells (aDCs) (r = 0.462), B cells (r = 0.585), CD8 T cells (r = 0.25), cytotoxic cells (r = 0.633), DCs (r = 0.294), eosinophils (r = 0.128), immature dendritic cells (iDCs) (r = 0.25), macrophages (r = 0.37), mast cells (r = 0.149), neutrophils (r = 0.117), natural killer (NK) CD56bright cells (r = -0.120), NK CD56dim cells (r = 0.229), NK cells (r = -0.186), plasmacytoid dendritic cells (pDCs) (r = 0.229), T cells (r = 0.822), T helper cells (r = 0.657), central memory T (TCM) cells (r = 0.416), effector memory T (TEM) cells (r = 0.282), T follicular helper (TFH) cells (r = 0.369), gamma delta T (Tγδ) cells (r = 0.115), Th1 cells (r = 0.588), Th17 cells (r = 0.104), regulatory T (Treg) cells (r = 0.335), and Th2 cells (r = 0.075), as determined using the Pearson correlation analysis (**Figures 9**, **S2**). Grouping by the median value of TRAT1 expression showed that the stromal, immune, and ESTIMATE scores were significantly different (**Figures 8D-F**), and the levels of B, CD8 T, cytotoxic, and other cells were significantly different between the high- and low-TRAT1 expression groups (**Figure S3**).

**Figure 10:**
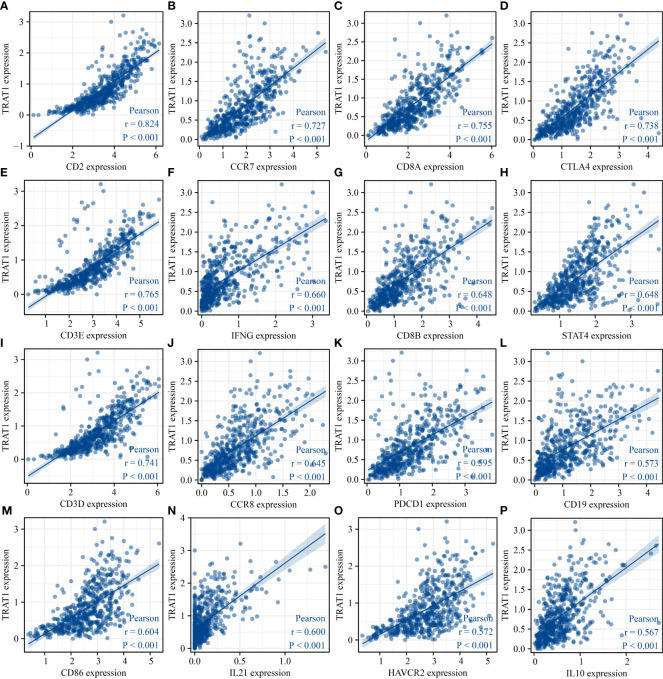
TRAT1 expression was significantly correlated with the levels of immune cell markers in LAC tissues. **(A)** CD2; **(B)** CCR7; **(C)** CD8A; **(D)** CTLA4; **(E)** CD3E; **(F)** IFNG; **(G)** CD8B; **(H)** STAT4; **(I)** CD3D; **(J)** CCR8; **(K)** PDCD1; **(L)** CD19; **(M)** CD86; **(N)** IL21; **(O)** HAVCR2; **(P)** IL10. LAC, lung adenocarcinoma.

**Figure 11:**
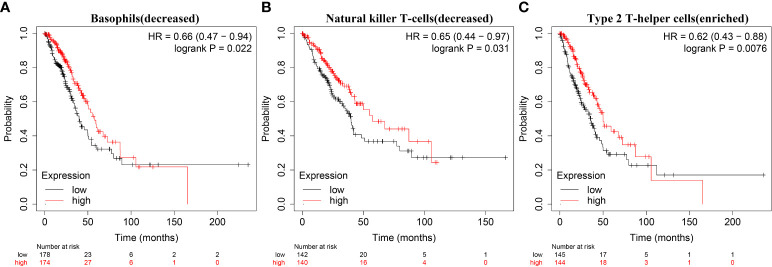
Decreased expression of TRAT1 was significantly associated with prognosis-related immune cells in patients with LAC. **(A)** Basophils (decreased); **(B)** Natural killer T-cells (decreased); **(C)** Type 2 T-helper cells (enriched). LAC, lung adenocarcinoma.

The expression data of immune cell markers in LAC tissues were obtained from TCGA database, and using correlation analysis, we found that the expression levels of TRAT1 correlated with the expression levels of CD8A, CD8B, CD3D, and others (**Figure 10** and **Table 3**). Grouping by the median value of TRAT1 expression showed that the levels of PDCD1, CD79A, CD19, and other immune cell markers were significantly different (**Figure S4**). In the K–M plotter database, K–M survival analysis revealed that the decreased expression of TRAT1 was significantly associated with poor prognosis in patients with LAC under the following conditions: basophils (decreased), natural killer T-cells (decreased), and type 2 T-helper cells (enriched) (**Figure 11**). Further, TRAT1 plays an important role in the progression of LAC and is a potential immune marker.

**Table 3:** Correlation between TRAT1 expression and expression levels of immune cell markers in LAC.

Markers	Cor	P	Markers	Cor	P	Markers	Cor	P
CD8A	0.755	<0.001	ITGAM	0.356	<0.001	IFNG	0.660	<0.001
CD8B	0.648	<0.001	CCR7	0.727	<0.001	TNF	0.330	<0.001
CD3D	0.741	<0.001	KIR2DL1	0.004	0.920	GATA3	0.327	<0.001
CD3E	0.765	<0.001	KIR2DL3	0.100	0.020	STAT6	0.024	0.583
CD2	0.824	<0.001	KIR2DL4	0.255	<0.001	STAT5A	0.486	<0.001
CD19	0.573	<0.001	KIR3DL1	0.091	0.036	IL13	0.271	<0.001
CD79A	0.472	<0.001	KIR3DL2	0.105	0.015	BCL6	0.059	0.174
CD86	0.604	<0.001	KIR3DL3	0.059	0.173	IL21	0.600	<0.001
CSF1R	0.431	<0.001	KIR2DS4	0.068	0.116	STAT3	-0.004	0.925
CCL2	0.283	<0.001	HLA-DPB1	0.484	<0.001	IL17A	0.275	<0.001
CD68	0.188	<0.001	HLA-DQB1	0.359	<0.001	FOXP3	0.518	<0.001
IL10	0.567	<0.001	HLA-DRA	0.515	<0.001	CCR8	0.645	<0.001
NOS2	0.035	0.423	HLA-DPA1	0.505	<0.001	STAT5B	0.340	<0.001
IRF5	0.270	<0.001	CD1C	0.306	<0.001	TGFB1	0.161	<0.001
PTGS2	-0.091	0.035	NRP1	0.251	<0.001	PDCD1	0.595	<0.001
CD163	0.447	<0.001	ITGAX	0.460	<0.001	CTLA4	0.738	<0.001
VSIG4	0.346	<0.001	TBX21	0.551	<0.001	LAG3	0.448	<0.001
MS4A4A	0.496	<0.001	STAT4	0.648	<0.001	HAVCR2	0.572	<0.001
CEACAM8	0.060	0.168	STAT1	0.559	<0.001	GZMB	0.495	<0.001

LAC, lung adenocarcinoma.

### TRAT1 was associated with RNA modification regulator genes

**Figure 12:**
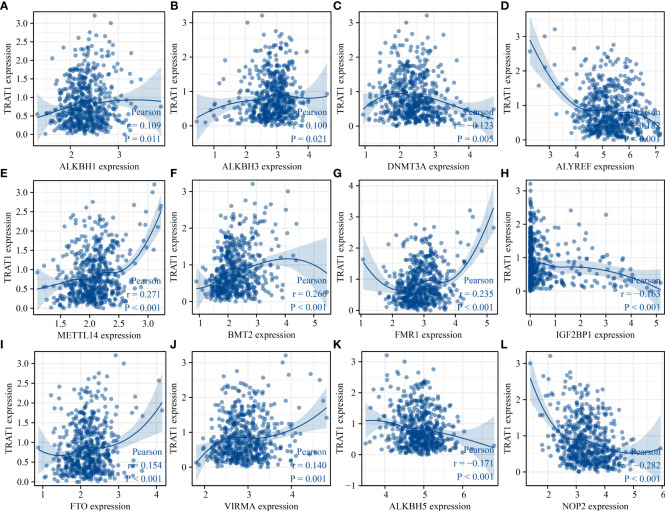
TRAT1 was associated with the RNA modification regulator genes. **(A)** ALKBH1; **(B)** ALKBH3; **(C)** DNMT3A; **(D)** ALYREF; **(E)** METTL14; **(F)** BMT2; **(G)** FMR1; **(H)** IGF2BP1; **(I)** FTO; **(J)** VIRMA; **(K)** ALKBH5; **(L)** NOP2.

Correlation analysis revealed that the expression levels of TRAT1 were associated with the expression levels of genes associated with RNA modifications, including *ALKBH1, ALKBH3, DNMT3A, ALYREF, BMT2, DNMT3A, FMR1, FTO, IGF2BP1, METTL14, NOP2, NSUN2, NSUN3, NSUN5, RBM15, RBM15B, RBMX, RRP8, TET2, TRDMT1, WTAP, YBX1, YTHDC1, YTHDC2, YTHDF1, YTHDF2, YTHDF3*, and *TRMT61A* (**Figures 12**, **S5**). Grouping by the median value of TRAT1 expression showed that the levels of *BMT2, ALYREF, ALKBH5, ALKBH3, ALKBH1, TET2, RRP8, RBMX, RBM15B*, and *RBM15* were significantly different between the high- and low-TRAT1 expression groups (**Figure S6**).

## Discussion

Studies have shown that oncogenes or tumour suppressor genes can promote or inhibit LAC progression (14, 16–18). The mRNA and protein expression levels of SPTBN2 in LAC tissues were significantly higher than those in normal tissues. Elevated SPTBN2 expression was associated with poor prognosis in patients with LAC. Interference with SPTBN2 inhibited the proliferation of A549 and H1299 cells. Interference with SPTBN2 expression resulted in decreased cell migration and invasion abilities compared to the control cell group (16). FYN is downregulated in LAC tissues and cells, and decreased FYN expression levels are associated with poor prognosis in patients with LAC. FYN overexpression significantly inhibited A549 cell viability, invasion, migration, and angiogenesis, accelerated cell apoptosis, and inhibited E-cadherin, vimentin, Snail, and PI3K/AKT protein expression in cancer cells (17). Our previous study found that the expression levels of TRAT1 in NSCLC, LAC, and LUSC tissues were significantly decreased, and the decreased expression level of TRAT1 was associated with poor prognosis in patients with cancer. In our study, we found that TRAT1 was associated with OS, PFS, smoking history, FEV1, PD-L1 expression, TMB, and response effects in patients with LAC. Additionally, TRAT1 co-expressed genes were found to be involved in cell differentiation, cell activation, cell-cell adhesion, and cell proliferation. In our cell model, elevated TRAT1 expression inhibited cell viability, migration, and invasion, and promoted apoptosis in A549 and H1299 cells. This suggests that TRAT1 may be involved in cell growth and migration as a tumour suppressor gene in LAC.

TRAT1 is a TCR regulator. We found that TRAT1 strongly co-expressed genes were involved in Th1, Th2, and Th17 cell differentiation and the TCR signalling pathway *via* KEGG analyses, which confirmed that TRAT1 was significantly associated with TCR. In addition, elevated TRAT1 expression might be involved in LAC progression through the Toll-like receptor and VEGF, MAPK, and MTOR signalling pathways, which are related to the progression of LAC (2, 19–23). For example, STAMBP expression is significantly increased in LAC cells and is closely related to tumour size, lymph node invasion, and tumour stage. STAMBP overexpression predicted shorter OS and disease-free survival in patients with LAC and promoted cell migration, invasion, and EGFR stabilization, whereas decreased STAMBP expression induces EGFR degradation. Small molecule inhibitors of EGFR and MAPK can block STAMBP-induced cell migration and invasion (21). DLC1 is significantly downregulated in LAC tissues and cells. DLC1 overexpression inhibits cell proliferation, migration, and invasion, whereas DLC1 downregulation promotes cell proliferation, migration, and invasion. DLC1 inhibits the proliferation and invasion of LAC cells by inhibiting the MAPK/ERK signalling pathway (22). However, the roles of TRAT1 overexpression and the Toll-like receptor, VEGF, MAPK, and other cancer signalling pathways need to be further confirmed in our models *via* western blotting. In addition, TRAT1 is associated with immunity (24, 25), and the immune microenvironment was significantly associated with LAC progression (26–28). For example, a meta-analysis by Brody et al. showed that high PD-L1 expression was associated with shorter survival. Anti-PD-1/PD-L1 drugs could improve the survival time of patients with cancer (28). In our research, we also found that the expression level of TRAT1 was significantly correlated with the levels of stromal, immune, and ESTIMATE scores, and was significantly correlated with the immune infiltration of CD8 T, cytotoxic, T, Th1, Th17, Treg, and other immune cell populations. TRAT1 expression was significantly correlated with the levels of CD8A, CD8B, CD3D, and other immune cell markers. The decreased expression of TRAT1 was associated with the prognosis of patients with LAC with basophils (decreased), natural killer T-cells (decreased), and type 2 T-helper cells (enriched). Preliminary evidence suggests that TRAT1 is an immune-related prognostic biomarker. However, the relationship between the expression level of TRAT1 in LAC and immune cells needs further study for verification.

Currently, RNA modification is one of the main mechanisms underlying cancer progression (29–34). For example, the m6A regulator gene METTL3 is overexpressed in lung cancer and associated with OS in patients with cancer. METTL3 overexpression can activate the PI3K/AKT/mTOR signalling pathway and mTOR-mediated protein synthesis in cancer cells to promote lung cancer progression (33). The m6A reader YTHDC2 is suppressed in LAC tissues. Decreased YTHDC2 expression levels are associated with poor clinical outcomes in patients with LAC. YTHDC2 reduces tumorigenesis in a mouse model and inhibits LAC cell progression. YTHDC2 inhibits cystine uptake through m6A recognition of the YTH domain, thus blocking the downstream antioxidation program. YTHDC2 destabilizes *SLC7A11* mRNA in an m6A-dependent manner (34). Therefore, we explored the relationship between TRAT1 expression and RNA modification regulatory genes. We found that TRAT1 expression levels correlated with m6A, m1A, and m5C modification regulator genes (*ALKBH1, ALKBH3, DNMT3A*, and *others*).

The shortcomings of our study were the lack of LAC tissue specimens, which resulted in us not able to confirm the expression of TRAT1 in LAC tissues and the relationship between the expression levels of TRAT1 and prognosis of patients with cancer, which needs to be verified in the future. In addition, the elevated expression of TRAT1 is associated with cancer signalling pathways such as Toll-like receptor, VEGF, and MAPK pathways, which should be further confirmed by western blotting in the future. In conclusion, we found that TRAT1 could inhibit the progression of LAC, which was related to the prognosis of patients with LAC based on the results of a comprehensive analysis. Furthermore, TRAT1 expression levels were significantly correlated with immune cell infiltration and immune cell marker levels in LAC, which is worthy of further exploration. Our findings will provide a basis for the further investigation of the interaction between TRAT1 and the immune microenvironment for regulating LAC progression.

## Data Availability Statement

Publicly available datasets were analysed in this study. Any additional data can be obtained from the corresponding author upon reasonable request.

## Author contributions

S-HW and S-SC conceptualized and designed the study. QG and C-YW performed the research experiments and plotted graphs. X-YX, QG, and S-SC wrote the manuscript and supervised the experiments. S-SC, ST, J-LC, J-HW, and YD performed cell culture experiments, constructed LAC cell models, and performed functional studies. All authors have read the manuscript and agreed to its publication.

## Funding

This work was supported by the Natural Science Foundation of China (No. 82100115, 82070431, 82100299, and 82100116), Natural Science Foundation of Hubei (No. 2020CFB392 and 2020CFB818), and Wuhan Municipal Health Commission Foundation(No. wx21Q38).

## Acknowledgments

We would like to thank TCGA, cBioPortal, muTarget and Kaplan-Meier plotter databases for providing the LAC data used in our study.

## Conflict of interest

The authors declare that the research was conducted in the absence of any commercial or financial relationships that could be construed as a potential conflict of interest.

## Publisher’s note

All claims expressed in this article are solely those of the authors and do not necessarily represent those of their affiliated organizations, or those of the publisher, the editors and the reviewers. Any product that may be evaluated in this article, or claim that may be made by its manufacturer, is not guaranteed or endorsed by the publisher.
