# Supplementary material for: TRAT1 overexpression delays cancer progression and is associated with immune infiltration in lung adenocarcinoma
Source: Front Oncol. 2022 Oct 6;12:960866. doi: 10.3389/fonc.2022.960866 (PMC9582843; doi:10.3389/fonc.2022.960866)
Supplement: Supplementary file 1 [file DataSheet_1.docx]

**Supplementary Figures**


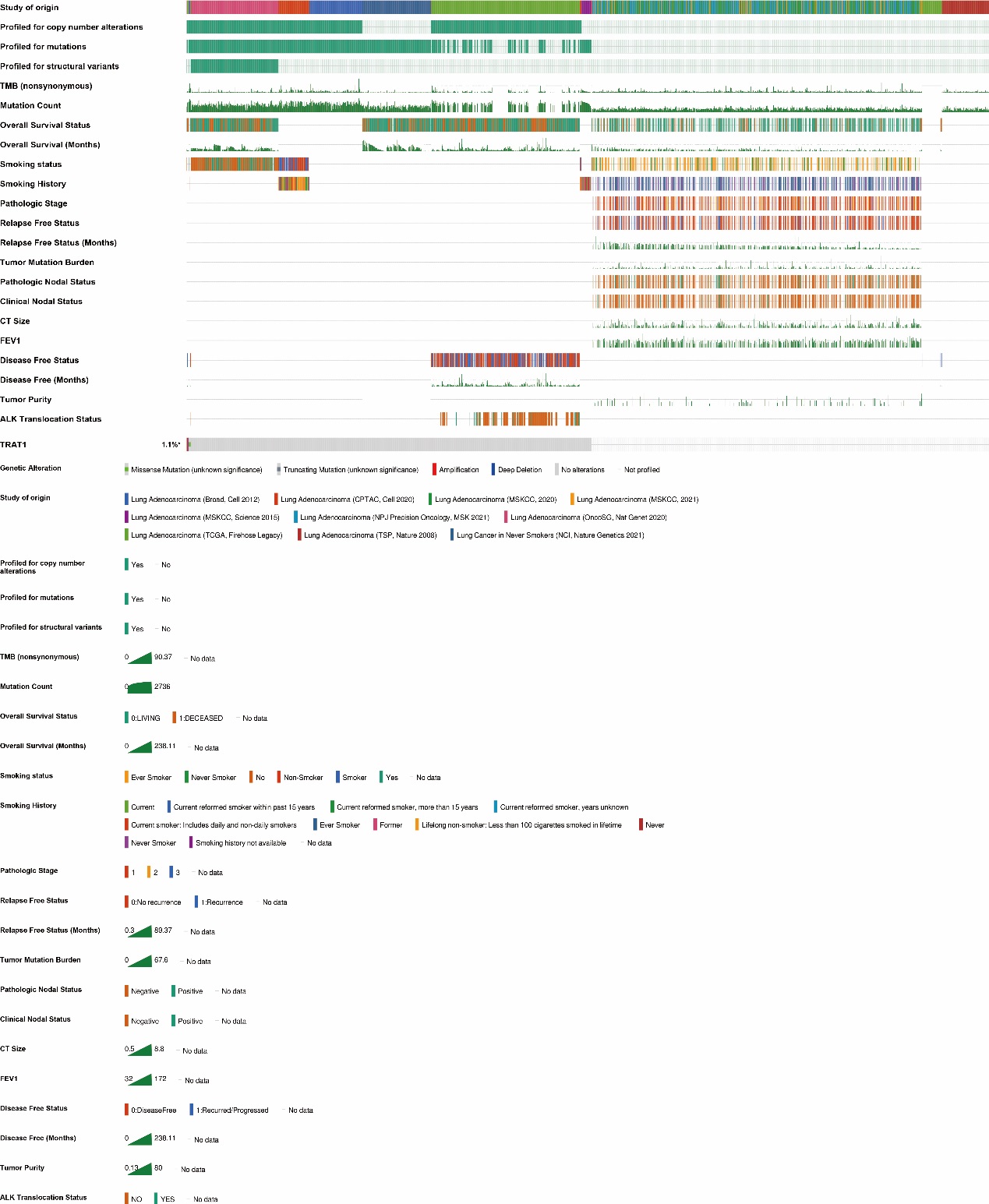
 Figure S1. Relationship between TRAT1 expression levels and clinical characteristics of patients with LAC in the cBioPortal database.


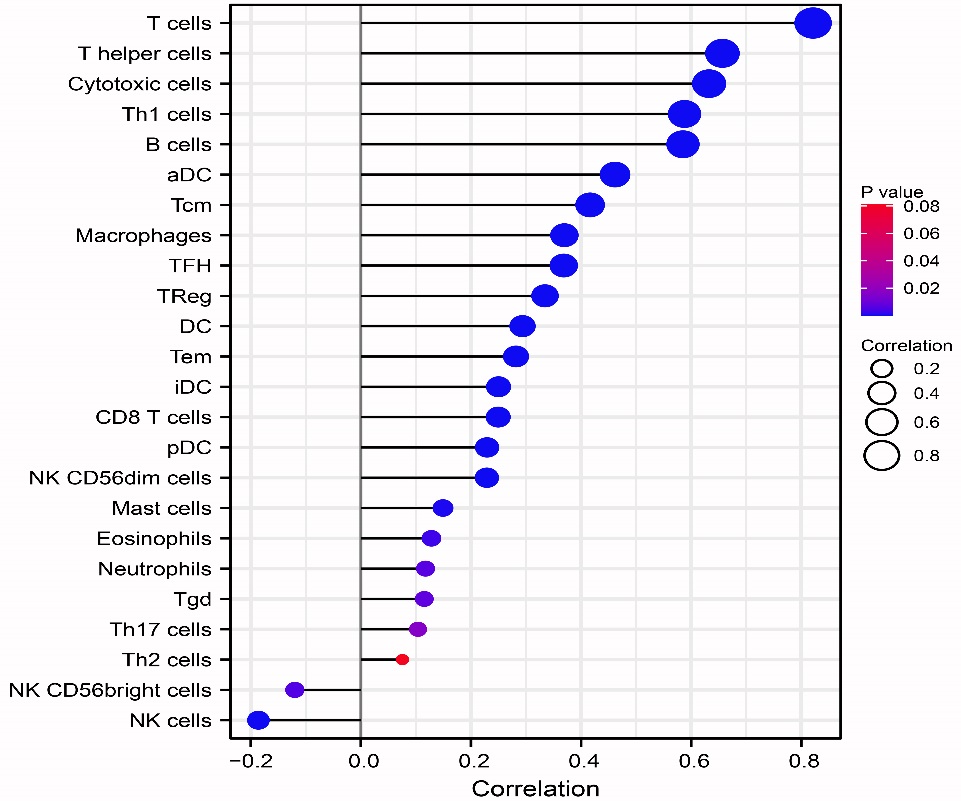


Figure S2. Correlation between TRAT1 expression and the levels of immune cells in LAC.

Note: LAC, lung adenocarcinoma.


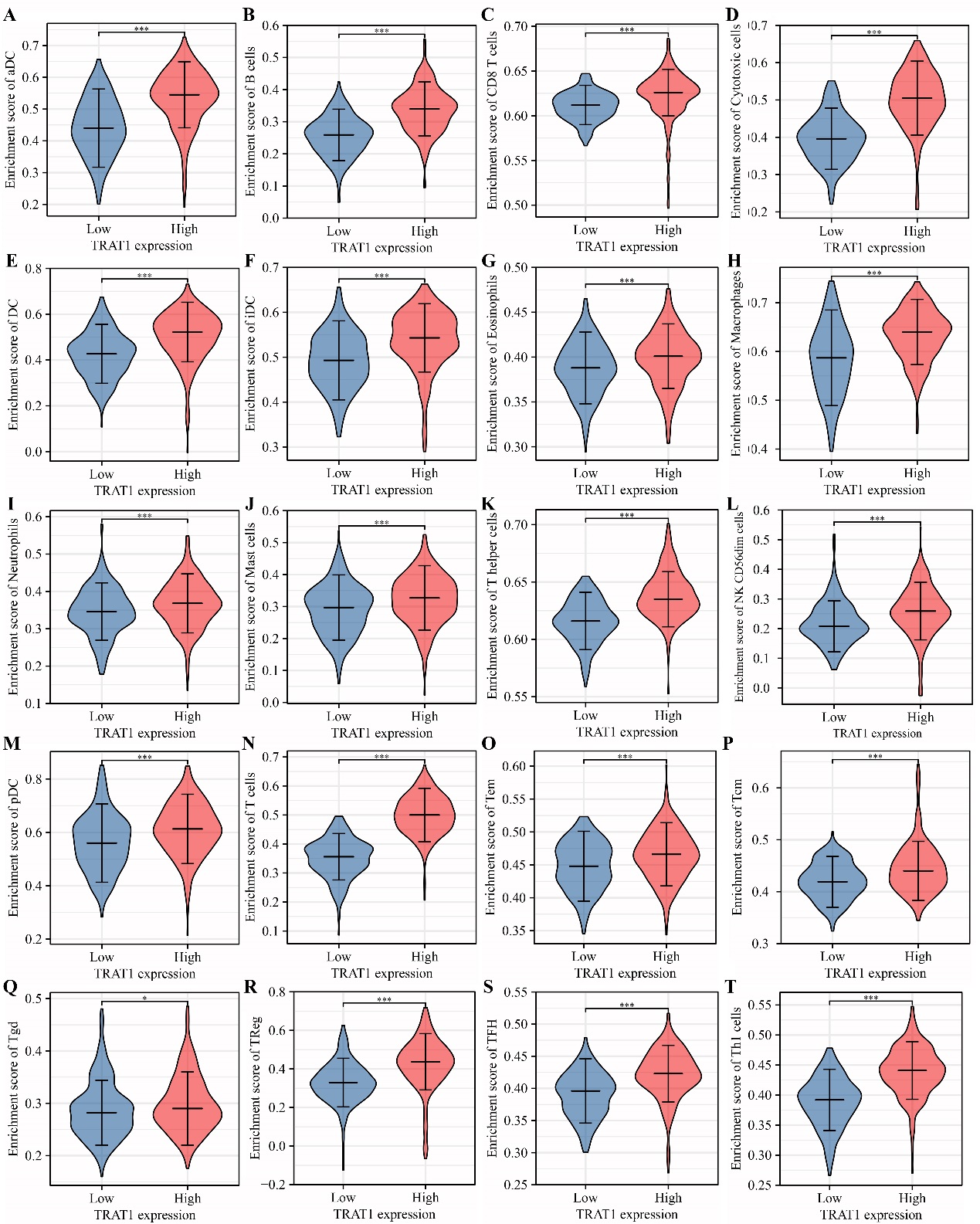


Figure S3. Expression levels of immune cells in high- and low-TRAT1 expression groups.


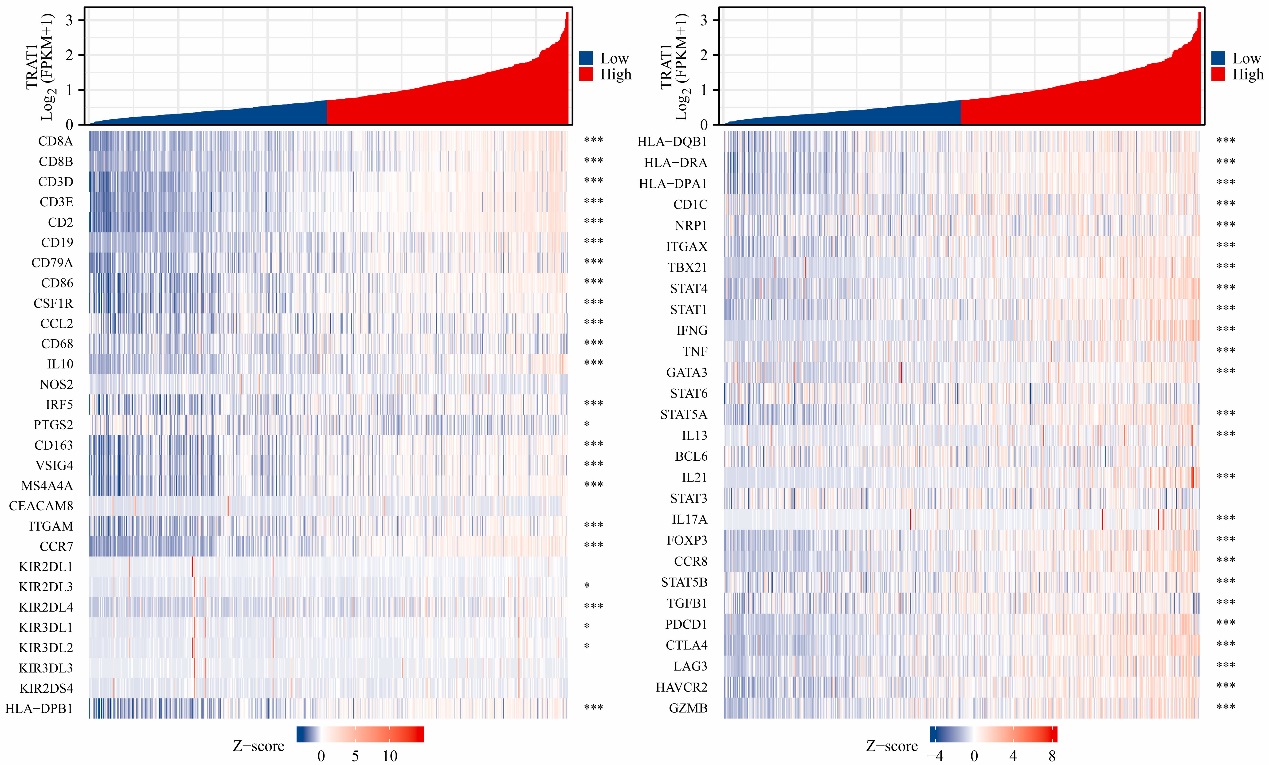
 Figure S4. expression levels of cellular markers in high- and low-TRAT1 expression groups.
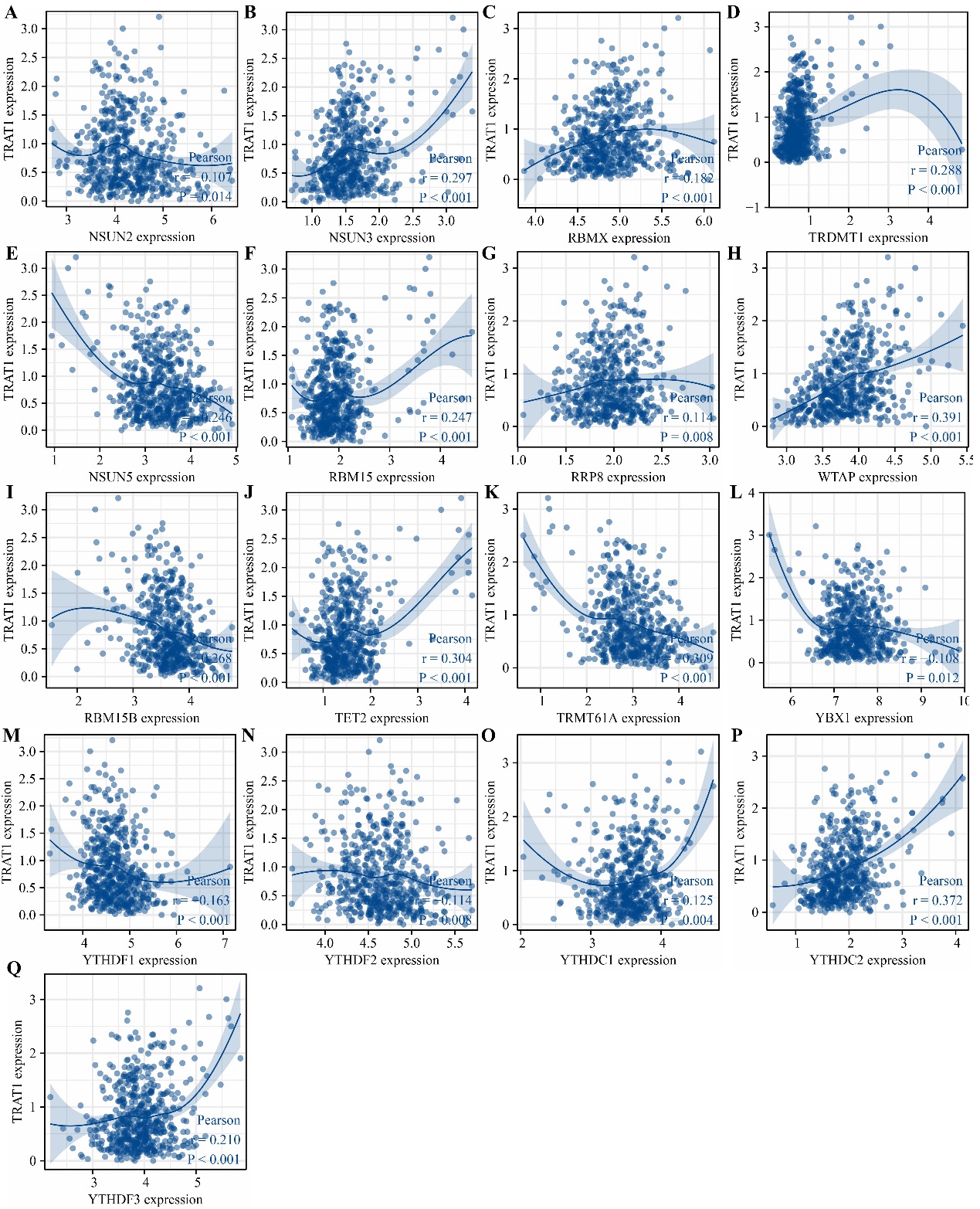


Figure S5. TRAT1-associated RNA modification regulator genes.


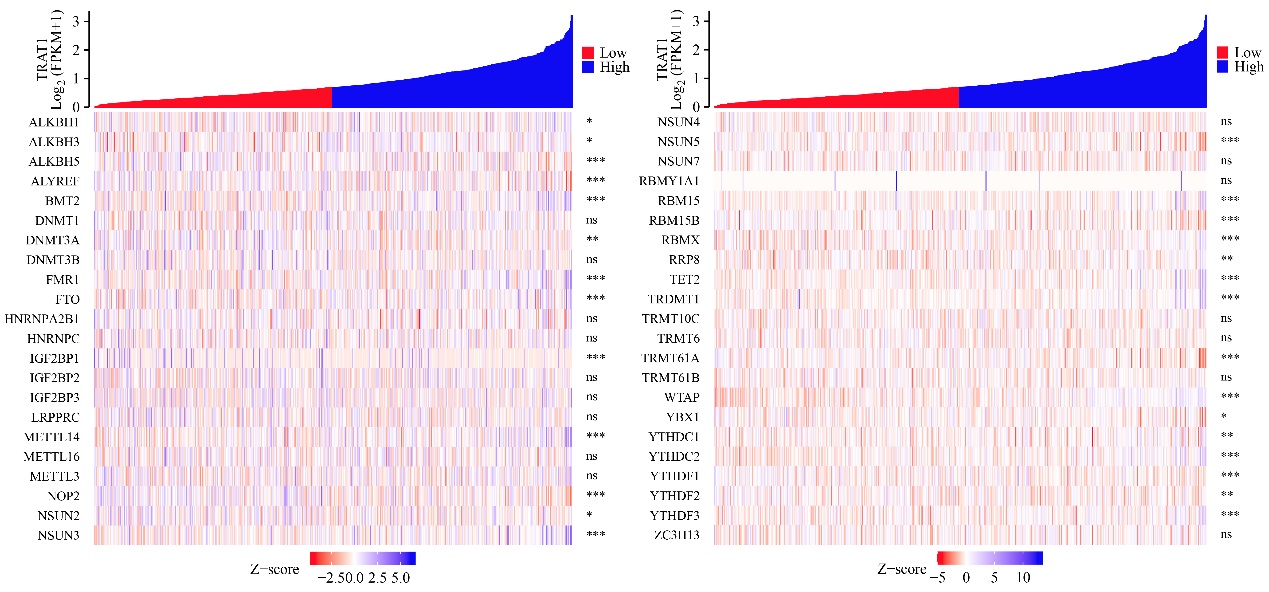


Figure S6. Expression levels of RNA modification regulator genes in high- and low-TRAT1 expression groups.

**Table S**

**Table S1**. TRAT1 co-expressed genes.

| Gene | Cor | p |
| --- | --- | --- |
| CPVL | 0.425 | 6.33E-25 |
| SP100 | 0.536 | 3.44E-41 |
| AKAP5 | 0.531 | 3.55E-40 |
| PARP8 | 0.557 | 6.33E-45 |
| LEF1 | 0.413 | 1.80E-23 |
| IL16 | 0.761 | 2.72E-102 |
| ZNF708 | 0.411 | 2.82E-23 |
| CSF1R | 0.431 | 1.41E-25 |
| ANK2 | 0.402 | 3.34E-22 |
| STAMBPL1 | 0.566 | 1.06E-46 |
| ITPRIPL1 | 0.516 | 9.81E-38 |
| CCR5 | 0.77 | 3.45E-106 |
| CXCL12 | 0.434 | 5.67E-26 |
| PLEKHO1 | 0.466 | 3.35E-30 |
| CD40LG | 0.779 | 4.63E-110 |
| STAT5A | 0.486 | 5.19E-33 |
| PLXNC1 | 0.676 | 1.03E-72 |
| CERKL | 0.525 | 3.58E-39 |
| GLIPR1 | 0.568 | 4.55E-47 |
| CEP85L | 0.563 | 3.85E-46 |
| ITGAE | 0.501 | 2.24E-35 |
| MPP1 | 0.418 | 4.65E-24 |
| LNPEP | 0.458 | 3.93E-29 |
| PDE4B | 0.585 | 1.61E-50 |
| THEMIS2 | 0.443 | 4.61E-27 |
| RAB33A | 0.555 | 1.34E-44 |
| RUBCNL | 0.603 | 3.54E-54 |
| PRKCB | 0.74 | 1.13E-93 |
| CRLF3 | 0.556 | 1.18E-44 |
| ABI3BP | 0.598 | 2.78E-53 |
| AIM2 | 0.457 | 6.47E-29 |
| CD209 | 0.403 | 2.88E-22 |
| ZNF267 | 0.562 | 6.28E-46 |
| GNA13 | 0.472 | 4.71E-31 |
| SLA2 | 0.781 | 3.85E-111 |
| GRAP2 | 0.733 | 2.81E-91 |
| MIER1 | 0.43 | 1.98E-25 |
| KCNAB2 | 0.411 | 3.58E-23 |
| IRF1 | 0.571 | 1.15E-47 |
| FMO2 | 0.434 | 5.26E-26 |
| ITGA4 | 0.687 | 7.74E-76 |
| CD247 | 0.818 | 2.31E-130 |
| CASP1 | 0.556 | 1.09E-44 |
| CD84 | 0.706 | 5.14E-82 |
| APOBEC3H | 0.5 | 3.67E-35 |
| SLC9A9 | 0.663 | 4.93E-69 |
| GLIPR2 | 0.407 | 8.91E-23 |
| SLAMF7 | 0.654 | 1.59E-66 |
| CCDC102B | 0.465 | 4.40E-30 |
| SLCO2B1 | 0.488 | 2.19E-33 |
| CD274 | 0.458 | 4.69E-29 |
| CXCR4 | 0.681 | 5.26E-74 |
| ITGAX | 0.46 | 2.57E-29 |
| TIMD4 | 0.407 | 9.84E-23 |
| POU2AF1 | 0.499 | 4.38E-35 |
| HCK | 0.413 | 2.08E-23 |
| SELP | 0.431 | 1.37E-25 |
| LGALS2 | 0.526 | 1.96E-39 |
| ZNF205 | -0.401 | 4.39E-22 |
| CR1 | 0.569 | 3.24E-47 |
| ALPK1 | 0.408 | 6.42E-23 |
| TNFAIP8L2 | 0.51 | 9.79E-37 |
| TIGIT | 0.824 | 1.54E-133 |
| TCF7 | 0.569 | 3.62E-47 |
| P2RX7 | 0.564 | 3.41E-46 |
| SLC7A7 | 0.494 | 3.42E-34 |
| MCOLN2 | 0.697 | 4.07E-79 |
| CD244 | 0.674 | 4.86E-72 |
| FLI1 | 0.668 | 1.64E-70 |
| SEPT1 | 0.689 | 1.93E-76 |
| NCF1 | 0.532 | 2.19E-40 |
| FCHSD2 | 0.487 | 3.36E-33 |
| TRAF1 | 0.607 | 4.05E-55 |
| EOMES | 0.797 | 5.34E-119 |
| IKZF1 | 0.834 | 1.41E-139 |
| WDFY1 | 0.435 | 4.59E-26 |
| LYSMD2 | 0.42 | 3.19E-24 |
| DCP2 | 0.47 | 1.09E-30 |
| RCSD1 | 0.764 | 1.36E-103 |
| GNG2 | 0.71 | 2.21E-83 |
| NR2F6 | -0.445 | 2.05E-27 |
| CD8B | 0.648 | 5.03E-65 |
| MILR1 | 0.45 | 4.85E-28 |
| CHRDL1 | 0.409 | 5.55E-23 |
| RBP5 | 0.585 | 1.72E-50 |
| CD48 | 0.801 | 1.16E-120 |
| PTAFR | 0.428 | 2.82E-25 |
| HELB | 0.473 | 4.15E-31 |
| BMP2K | 0.569 | 2.88E-47 |
| GBP5 | 0.705 | 1.85E-81 |
| HAVCR2 | 0.572 | 6.67E-48 |
| MCUB | 0.426 | 4.70E-25 |
| PLA2G4C | 0.465 | 4.94E-30 |
| RAC2 | 0.483 | 1.37E-32 |
| CXCL11 | 0.563 | 3.99E-46 |
| IRF2 | 0.402 | 3.35E-22 |
| TLR7 | 0.643 | 8.62E-64 |
| GIMAP8 | 0.588 | 5.16E-51 |
| IL10RA | 0.735 | 6.52E-92 |
| CNRIP1 | 0.41 | 4.43E-23 |
| FCGR2A | 0.402 | 3.68E-22 |
| EBI3 | 0.541 | 4.98E-42 |
| C11orf21 | 0.525 | 3.18E-39 |
| SNX20 | 0.677 | 6.24E-73 |
| DDHD1 | 0.438 | 1.93E-26 |
| LAX1 | 0.681 | 5.24E-74 |
| CCL19 | 0.621 | 2.65E-58 |
| ITGB7 | 0.609 | 1.37E-55 |
| LIPA | 0.452 | 2.98E-28 |
| MAF | 0.457 | 5.37E-29 |
| DUSP2 | 0.509 | 1.26E-36 |
| BLNK | 0.464 | 6.65E-30 |
| LAIR1 | 0.531 | 3.20E-40 |
| EVI2B | 0.737 | 1.15E-92 |
| TCAF2 | 0.425 | 6.83E-25 |
| DOCK4 | 0.431 | 1.41E-25 |
| CD80 | 0.657 | 1.67E-67 |
| S1PR1 | 0.411 | 2.82E-23 |
| SYNE3 | 0.549 | 1.87E-43 |
| LRRC8C | 0.619 | 5.95E-58 |
| CCL4L2 | 0.437 | 2.08E-26 |
| CD38 | 0.458 | 3.96E-29 |
| TP53INP1 | 0.456 | 7.35E-29 |
| MAFB | 0.497 | 1.17E-34 |
| CXCR3 | 0.552 | 5.06E-44 |
| LAP3 | 0.499 | 5.41E-35 |
| STAT4 | 0.648 | 4.27E-65 |
| SLA | 0.706 | 7.21E-82 |
| CEPT1 | 0.442 | 4.93E-27 |
| CD86 | 0.604 | 2.14E-54 |
| NKG7 | 0.585 | 1.58E-50 |
| IFIH1 | 0.401 | 4.04E-22 |
| ATM | 0.498 | 6.53E-35 |
| ABCB1 | 0.435 | 4.69E-26 |
| FCRL2 | 0.475 | 2.17E-31 |
| DOCK8 | 0.67 | 6.18E-71 |
| SIT1 | 0.742 | 1.27E-94 |
| ETS1 | 0.622 | 1.44E-58 |
| THEMIS | 0.931 | 1.03E-234 |
| ADAMDEC1 | 0.622 | 1.29E-58 |
| CD101 | 0.413 | 1.74E-23 |
| SIRPB2 | 0.478 | 7.41E-32 |
| ZFP36L2 | 0.418 | 5.24E-24 |
| RASA2 | 0.485 | 6.22E-33 |
| XRN1 | 0.529 | 6.24E-40 |
| PARP15 | 0.582 | 8.41E-50 |
| PSMB9 | 0.457 | 6.69E-29 |
| GIMAP7 | 0.722 | 3.70E-87 |
| CALHM6 | 0.605 | 8.76E-55 |
| CCL4 | 0.623 | 6.26E-59 |
| NLRC5 | 0.594 | 2.88E-52 |
| MAT2B | 0.445 | 2.09E-27 |
| LYRM7 | 0.403 | 2.94E-22 |
| CD300LF | 0.45 | 5.04E-28 |
| LYZ | 0.468 | 1.69E-30 |
| HLA-DMB | 0.584 | 2.47E-50 |
| GPRIN3 | 0.473 | 3.68E-31 |
| SPOCK2 | 0.433 | 8.48E-26 |
| HLA-DPB1 | 0.484 | 9.97E-33 |
| TDO2 | 0.438 | 1.97E-26 |
| CASS4 | 0.54 | 7.19E-42 |
| MYO5A | 0.428 | 2.82E-25 |
| ZBED2 | 0.407 | 8.99E-23 |
| F13A1 | 0.403 | 2.46E-22 |
| VPS13C | 0.414 | 1.48E-23 |
| BIN2 | 0.688 | 3.38E-76 |
| MNDA | 0.526 | 2.16E-39 |
| CYLD | 0.642 | 2.25E-63 |
| FCRL6 | 0.56 | 1.44E-45 |
| DDX60 | 0.447 | 1.22E-27 |
| SLC4A7 | 0.43 | 1.84E-25 |
| NCF4 | 0.431 | 1.30E-25 |
| UBE2J1 | 0.494 | 3.14E-34 |
| LY75 | 0.48 | 4.11E-32 |
| PLCL1 | 0.445 | 2.01E-27 |
| MANEA | 0.509 | 1.49E-36 |
| RASAL3 | 0.636 | 5.50E-62 |
| CCND2 | 0.505 | 5.42E-36 |
| GZMM | 0.448 | 8.68E-28 |
| SESN1 | 0.418 | 4.55E-24 |
| PRF1 | 0.537 | 2.37E-41 |
| BAG6 | -0.408 | 6.36E-23 |
| USP15 | 0.478 | 6.86E-32 |
| BANK1 | 0.573 | 4.64E-48 |
| ACAP1 | 0.696 | 1.36E-78 |
| WDFY4 | 0.443 | 3.48E-27 |
| FGD2 | 0.626 | 1.59E-59 |
| TNFAIP3 | 0.608 | 2.47E-55 |
| IRF8 | 0.749 | 2.69E-97 |
| LXN | 0.437 | 2.61E-26 |
| C1S | 0.414 | 1.33E-23 |
| CTSW | 0.407 | 9.18E-23 |
| EVI2A | 0.742 | 1.62E-94 |
| FCER1G | 0.42 | 2.62E-24 |
| ZEB1 | 0.449 | 6.15E-28 |
| PTPRC | 0.836 | 4.20E-141 |
| CGGBP1 | 0.416 | 8.38E-24 |
| ZEB2 | 0.599 | 2.09E-53 |
| SETX | 0.433 | 8.20E-26 |
| CEP128 | 0.403 | 2.71E-22 |
| NPAT | 0.408 | 6.76E-23 |
| PSTPIP1 | 0.664 | 2.00E-69 |
| PDCD1LG2 | 0.67 | 7.26E-71 |
| SASH3 | 0.7 | 4.82E-80 |
| LST1 | 0.437 | 2.68E-26 |
| CD8A | 0.755 | 1.14E-99 |
| LHFPL2 | 0.418 | 4.67E-24 |
| CD79B | 0.577 | 6.69E-49 |
| IL15 | 0.625 | 2.09E-59 |
| RAB39B | 0.459 | 3.29E-29 |
| IGFLR1 | 0.431 | 1.35E-25 |
| EPSTI1 | 0.535 | 5.37E-41 |
| PAPLN | 0.443 | 3.96E-27 |
| SFMBT2 | 0.45 | 4.69E-28 |
| GAPT | 0.61 | 7.99E-56 |
| CPNE5 | 0.455 | 1.07E-28 |
| DNAJB14 | 0.444 | 3.17E-27 |
| CD33 | 0.458 | 3.67E-29 |
| SLC31A2 | 0.53 | 4.15E-40 |
| ZNF430 | 0.401 | 4.04E-22 |
| CLUH | -0.402 | 3.25E-22 |
| XAF1 | 0.453 | 1.97E-28 |
| VPREB3 | 0.469 | 1.25E-30 |
| PTPN7 | 0.718 | 7.46E-86 |
| ICAM3 | 0.657 | 2.78E-67 |
| LIMS1 | 0.419 | 3.61E-24 |
| OR2I1P | 0.533 | 1.16E-40 |
| LTA | 0.665 | 1.61E-69 |
| STX11 | 0.56 | 1.51E-45 |
| STK4 | 0.603 | 2.61E-54 |
| SRGN | 0.497 | 8.95E-35 |
| INTS6L | 0.464 | 7.12E-30 |
| CD6 | 0.743 | 7.79E-95 |
| CD53 | 0.709 | 8.45E-83 |
| TNFSF13B | 0.754 | 3.27E-99 |
| PRKCQ | 0.549 | 2.29E-43 |
| PNOC | 0.474 | 2.65E-31 |
| ZAP70 | 0.686 | 1.74E-75 |
| FPR3 | 0.557 | 5.34E-45 |
| PREX1 | 0.56 | 1.80E-45 |
| DPEP2 | 0.439 | 1.25E-26 |
| MPEG1 | 0.638 | 2.09E-62 |
| HLA-DPA1 | 0.505 | 6.46E-36 |
| CCDC200 | 0.419 | 3.64E-24 |
| FOXN2 | 0.486 | 4.29E-33 |
| SAMHD1 | 0.583 | 3.97E-50 |
| RGS1 | 0.586 | 1.47E-50 |
| ZC3H12D | 0.623 | 6.15E-59 |
| CLEC2B | 0.416 | 8.01E-24 |
| NCR3 | 0.67 | 4.87E-71 |
| PLEKHA2 | 0.501 | 2.47E-35 |
| ARHGEF6 | 0.719 | 2.53E-86 |
| PDCD1 | 0.595 | 1.96E-52 |
| GCSAM | 0.514 | 2.25E-37 |
| CXCR6 | 0.864 | 8.94E-161 |
| AKAP7 | 0.513 | 2.85E-37 |
| DVL1 | -0.402 | 3.61E-22 |
| ARAP2 | 0.476 | 1.36E-31 |
| RASSF5 | 0.461 | 1.69E-29 |
| CD37 | 0.577 | 1.03E-48 |
| FCRL1 | 0.638 | 2.28E-62 |
| PIK3R1 | 0.448 | 9.63E-28 |
| ARHGAP18 | 0.476 | 1.41E-31 |
| CYTIP | 0.794 | 3.32E-117 |
| NELL2 | 0.544 | 1.65E-42 |
| CD3D | 0.741 | 2.01E-94 |
| BHLHE22 | 0.486 | 4.55E-33 |
| APOBEC3D | 0.531 | 3.37E-40 |
| RASGRP2 | 0.522 | 8.91E-39 |
| FAM49A | 0.513 | 2.73E-37 |
| GPR174 | 0.878 | 1.80E-172 |
| NCKAP1L | 0.694 | 4.25E-78 |
| CCDC152 | 0.461 | 1.77E-29 |
| ASB2 | 0.619 | 7.57E-58 |
| RAB8B | 0.623 | 6.98E-59 |
| CENPC | 0.416 | 7.65E-24 |
| PIK3R6 | 0.401 | 4.83E-22 |
| CTLA4 | 0.738 | 3.26E-93 |
| GPR82 | 0.545 | 1.02E-42 |
| FAM78A | 0.661 | 2.39E-68 |
| CALCRL | 0.423 | 1.17E-24 |
| HCFC2 | 0.484 | 1.04E-32 |
| HLA-DQA1 | 0.567 | 9.14E-47 |
| PARP9 | 0.405 | 1.74E-22 |
| TLR6 | 0.446 | 1.76E-27 |
| IL33 | 0.444 | 2.79E-27 |
| CD200R1 | 0.779 | 2.40E-110 |
| TIFA | 0.423 | 1.15E-24 |
| P2RY10 | 0.875 | 6.33E-170 |
| PLEK | 0.684 | 4.14E-75 |
| P2RY8 | 0.649 | 2.12E-65 |
| PLXDC2 | 0.442 | 5.98E-27 |
| FCRL3 | 0.738 | 2.96E-93 |
| IFFO1 | 0.407 | 8.22E-23 |
| ABCA1 | 0.412 | 2.22E-23 |
| DMXL1 | 0.458 | 4.66E-29 |
| APBB1IP | 0.598 | 2.70E-53 |
| SNX10 | 0.516 | 9.64E-38 |
| CD69 | 0.75 | 6.23E-98 |
| SPN | 0.581 | 1.14E-49 |
| CDC42SE2 | 0.578 | 5.01E-49 |
| PARVG | 0.513 | 2.58E-37 |
| VNN2 | 0.491 | 9.34E-34 |
| OGFRL1 | 0.422 | 1.65E-24 |
| P2RX5 | 0.504 | 8.78E-36 |
| CD22 | 0.532 | 2.06E-40 |
| LACTB | 0.457 | 5.82E-29 |
| LY9 | 0.775 | 2.77E-108 |
| TMEM156 | 0.412 | 2.48E-23 |
| MGAT4A | 0.41 | 3.90E-23 |
| RCBTB2 | 0.469 | 1.37E-30 |
| CRTAM | 0.81 | 1.27E-125 |
| TNFSF4 | 0.406 | 1.23E-22 |
| RASGRP3 | 0.612 | 2.69E-56 |
| HERPUD1 | 0.424 | 1.07E-24 |
| ERAP1 | 0.476 | 1.35E-31 |
| PIK3IP1 | 0.419 | 3.34E-24 |
| IL18BP | 0.486 | 4.06E-33 |
| CXCL9 | 0.67 | 7.05E-71 |
| MALT1 | 0.401 | 4.25E-22 |
| SH3BGRL | 0.474 | 2.78E-31 |
| PIK3CG | 0.723 | 1.46E-87 |
| LAMA2 | 0.419 | 3.84E-24 |
| GPR171 | 0.664 | 2.07E-69 |
| ADA2 | 0.449 | 6.79E-28 |
| LYST | 0.431 | 1.16E-25 |
| ZBTB1 | 0.452 | 2.43E-28 |
| PIEZO2 | 0.402 | 2.99E-22 |
| CCR2 | 0.711 | 1.25E-83 |
| MMP25 | 0.471 | 6.32E-31 |
| C3AR1 | 0.509 | 1.17E-36 |
| PTGER4 | 0.647 | 1.03E-64 |
| WARS | 0.447 | 1.13E-27 |
| KBTBD8 | 0.714 | 1.15E-84 |
| INPP5D | 0.597 | 5.12E-53 |
| ABCA6 | 0.535 | 6.54E-41 |
| HLA-B | 0.414 | 1.47E-23 |
| CCDC50 | 0.433 | 6.58E-26 |
| MAN1A1 | 0.423 | 1.34E-24 |
| CYP1B1 | 0.425 | 6.43E-25 |
| IL18R1 | 0.517 | 6.81E-38 |
| KLRB1 | 0.812 | 1.43E-126 |
| ADAP2 | 0.43 | 1.71E-25 |
| LACC1 | 0.409 | 4.84E-23 |
| CCR1 | 0.464 | 6.99E-30 |
| FCRLA | 0.589 | 3.52E-51 |
| ZNF101 | 0.652 | 3.71E-66 |
| CARD8 | 0.613 | 1.68E-56 |
| IRAK4 | 0.455 | 1.03E-28 |
| EPS15 | 0.532 | 1.78E-40 |
| SENP7 | 0.43 | 1.63E-25 |
| GBP2 | 0.492 | 6.91E-34 |
| MS4A4A | 0.496 | 1.68E-34 |
| WDR7 | 0.432 | 9.83E-26 |
| TNFRSF13C | 0.537 | 2.30E-41 |
| C1orf162 | 0.491 | 7.72E-34 |
| SIRPG | 0.745 | 1.36E-95 |
| GBP1 | 0.605 | 8.71E-55 |
| IRF4 | 0.629 | 2.79E-60 |
| FGR | 0.42 | 2.49E-24 |
| CXCL10 | 0.594 | 3.00E-52 |
| PIM2 | 0.544 | 1.38E-42 |
| CCR4 | 0.776 | 9.67E-109 |
| GAB3 | 0.683 | 1.17E-74 |
| ZNF25 | 0.428 | 2.71E-25 |
| EPB41L2 | 0.47 | 1.04E-30 |
| BLK | 0.574 | 2.79E-48 |
| EAF2 | 0.487 | 3.60E-33 |
| CPED1 | 0.509 | 1.26E-36 |
| PRKD3 | 0.408 | 7.99E-23 |
| KCNN3 | 0.414 | 1.28E-23 |
| HS3ST3B1 | 0.507 | 2.32E-36 |
| HAPLN3 | 0.472 | 5.25E-31 |
| JAK2 | 0.678 | 2.09E-73 |
| GYPC | 0.483 | 1.32E-32 |
| PRKCH | 0.578 | 5.97E-49 |
| TNFRSF9 | 0.578 | 4.43E-49 |
| SCIMP | 0.709 | 5.05E-83 |
| ZNF366 | 0.41 | 4.20E-23 |
| AIF1 | 0.494 | 3.21E-34 |
| LSAMP | 0.453 | 1.88E-28 |
| SAMSN1 | 0.739 | 2.22E-93 |
| BTN3A3 | 0.623 | 9.33E-59 |
| VCAM1 | 0.493 | 3.74E-34 |
| HGF | 0.427 | 3.99E-25 |
| SEPT6 | 0.543 | 2.00E-42 |
| PPP3CC | 0.548 | 2.42E-43 |
| IQGAP2 | 0.438 | 1.82E-26 |
| STX7 | 0.446 | 1.84E-27 |
| GSAP | 0.403 | 2.84E-22 |
| IFNAR2 | 0.404 | 1.78E-22 |
| SPIB | 0.57 | 1.68E-47 |
| CREBL2 | 0.428 | 2.99E-25 |
| SELL | 0.681 | 4.18E-74 |
| LDLRAD4 | 0.459 | 2.92E-29 |
| CCR7 | 0.727 | 3.33E-89 |
| CD2 | 0.824 | 6.76E-134 |
| MYO1F | 0.535 | 6.61E-41 |
| DOCK2 | 0.728 | 2.96E-89 |
| GIMAP6 | 0.701 | 2.99E-80 |
| ZNF699 | 0.418 | 4.65E-24 |
| LPXN | 0.7 | 5.40E-80 |
| GAS7 | 0.5 | 3.24E-35 |
| TRIM69 | 0.497 | 1.18E-34 |
| CXorf21 | 0.621 | 2.31E-58 |
| SOD2 | 0.433 | 7.91E-26 |
| THAP4 | -0.419 | 3.33E-24 |
| ENTPD1 | 0.594 | 2.76E-52 |
| TXK | 0.482 | 1.51E-32 |
| CYB5R4 | 0.441 | 7.30E-27 |
| CNOT6L | 0.596 | 1.01E-52 |
| SMAP2 | 0.498 | 8.30E-35 |
| CD4 | 0.552 | 4.46E-44 |
| ITM2A | 0.621 | 1.75E-58 |
| FERMT3 | 0.452 | 3.06E-28 |
| LCP1 | 0.603 | 2.23E-54 |
| QKI | 0.494 | 3.17E-34 |
| CTSS | 0.566 | 1.20E-46 |
| SERPINB9 | 0.552 | 6.16E-44 |
| BIRC3 | 0.524 | 5.51E-39 |
| MAP3K1 | 0.463 | 8.37E-30 |
| IL24 | 0.653 | 2.00E-66 |
| C3orf38 | 0.405 | 1.44E-22 |
| BTLA | 0.875 | 4.02E-170 |
| FCER2 | 0.413 | 1.77E-23 |
| CD1D | 0.548 | 2.42E-43 |
| SLC8A1 | 0.57 | 1.89E-47 |
| PTPN22 | 0.637 | 2.59E-62 |
| LILRB1 | 0.586 | 1.11E-50 |
| CLEC7A | 0.473 | 3.27E-31 |
| LINS1 | 0.441 | 6.56E-27 |
| PSTPIP2 | 0.43 | 1.90E-25 |
| FCGR3A | 0.448 | 8.04E-28 |
| CXCL13 | 0.665 | 1.09E-69 |
| STAP1 | 0.684 | 5.94E-75 |
| XCL2 | 0.586 | 1.06E-50 |
| HLA-E | 0.475 | 1.98E-31 |
| RASSF4 | 0.479 | 4.30E-32 |
| CYSLTR1 | 0.401 | 4.15E-22 |
| PARP11 | 0.451 | 3.98E-28 |
| MBNL3 | 0.415 | 1.00E-23 |
| SYNPO2 | 0.43 | 1.97E-25 |
| CLEC1A | 0.438 | 1.97E-26 |
| TNFRSF17 | 0.552 | 5.54E-44 |
| AGAP2 | 0.608 | 2.72E-55 |
| BCL2A1 | 0.432 | 1.09E-25 |
| FAM13B | 0.401 | 4.62E-22 |
| STK17B | 0.542 | 3.63E-42 |
| FAS | 0.501 | 2.53E-35 |
| TLR8 | 0.638 | 1.98E-62 |
| BTN3A1 | 0.629 | 2.52E-60 |
| CST7 | 0.521 | 1.55E-38 |
| CREBRF | 0.504 | 7.67E-36 |
| CTSO | 0.406 | 1.33E-22 |
| SUSD3 | 0.522 | 8.54E-39 |
| IL21R | 0.677 | 5.09E-73 |
| NFAM1 | 0.482 | 1.90E-32 |
| CYP7B1 | 0.421 | 2.18E-24 |
| AMPD1 | 0.419 | 3.51E-24 |
| OSTM1 | 0.475 | 1.83E-31 |
| NAAA | 0.413 | 1.99E-23 |
| MSL3 | 0.508 | 1.85E-36 |
| TRIM22 | 0.6 | 1.60E-53 |
| PAX5 | 0.494 | 2.81E-34 |
| TAGAP | 0.802 | 1.85E-121 |
| ZBTB25 | 0.432 | 1.02E-25 |
| ARHGAP15 | 0.842 | 4.70E-145 |
| MYCBP2 | 0.436 | 2.93E-26 |
| LCP2 | 0.728 | 2.42E-89 |
| STK10 | 0.501 | 2.79E-35 |
| FLT3LG | 0.441 | 8.25E-27 |
| DHCR7 | -0.403 | 2.77E-22 |
| CARD16 | 0.466 | 3.74E-30 |
| TBC1D10C | 0.682 | 1.86E-74 |
| KCNA3 | 0.437 | 2.28E-26 |
| LY86 | 0.457 | 5.78E-29 |
| TRIM38 | 0.414 | 1.28E-23 |
| ZNF136 | 0.459 | 3.20E-29 |
| IL7 | 0.523 | 5.87E-39 |
| TAP2 | 0.458 | 4.29E-29 |
| TCF4 | 0.456 | 7.62E-29 |
| RNF125 | 0.496 | 1.37E-34 |
| NFATC2 | 0.619 | 5.85E-58 |
| HSD11B1 | 0.568 | 5.63E-47 |
| CD72 | 0.622 | 1.28E-58 |
| DOK2 | 0.41 | 4.48E-23 |
| RGS18 | 0.672 | 2.10E-71 |
| HCLS1 | 0.553 | 3.89E-44 |
| KAT2B | 0.551 | 6.70E-44 |
| PATL2 | 0.591 | 1.13E-51 |
| IL10 | 0.567 | 7.81E-47 |
| FGFR1OP2 | 0.454 | 1.45E-28 |
| COL6A5 | 0.505 | 6.05E-36 |
| C9orf72 | 0.49 | 1.00E-33 |
| ADAM19 | 0.455 | 1.07E-28 |
| CLEC4A | 0.573 | 6.42E-48 |
| SLAMF8 | 0.545 | 1.18E-42 |
| FASLG | 0.679 | 1.55E-73 |
| TM6SF1 | 0.506 | 3.40E-36 |
| TLR1 | 0.545 | 8.83E-43 |
| APLF | 0.41 | 4.27E-23 |
| SKAP1 | 0.407 | 9.52E-23 |
| C21orf91 | 0.422 | 1.77E-24 |
| BHLHE41 | 0.422 | 1.81E-24 |
| ZNF683 | 0.502 | 1.88E-35 |
| SGTB | 0.547 | 3.95E-43 |
| NUGGC | 0.626 | 1.81E-59 |
| PYHIN1 | 0.912 | 4.13E-208 |
| RASGEF1B | 0.56 | 1.81E-45 |
| SP110 | 0.529 | 5.65E-40 |
| UBASH3A | 0.796 | 1.72E-118 |
| RORA | 0.484 | 8.11E-33 |
| PLEKHO2 | 0.477 | 1.06E-31 |
| DPYD | 0.404 | 2.13E-22 |
| TENT2 | 0.496 | 1.37E-34 |
| SIGLEC8 | 0.539 | 1.13E-41 |
| ARHGEF3 | 0.503 | 1.19E-35 |
| TLR4 | 0.613 | 1.40E-56 |
| HELQ | 0.436 | 2.90E-26 |
| RNASE6 | 0.558 | 4.10E-45 |
| ABI3 | 0.483 | 1.31E-32 |
| CD79A | 0.472 | 4.36E-31 |
| FNBP1 | 0.581 | 1.55E-49 |
| CEACAM21 | 0.441 | 7.95E-27 |
| ATP8B4 | 0.525 | 2.63E-39 |
| LRMP | 0.605 | 1.27E-54 |
| PLCL2 | 0.555 | 1.31E-44 |
| TAP1 | 0.429 | 2.24E-25 |
| RIPOR2 | 0.534 | 1.05E-40 |
| IGSF6 | 0.642 | 1.73E-63 |
| GM2A | 0.417 | 6.97E-24 |
| DOCK11 | 0.573 | 6.22E-48 |
| JAK3 | 0.597 | 7.01E-53 |
| PIP4K2A | 0.5 | 3.50E-35 |
| CLECL1 | 0.757 | 1.67E-100 |
| P2RY14 | 0.644 | 6.68E-64 |
| CCL5 | 0.657 | 2.77E-67 |
| GZMB | 0.495 | 1.78E-34 |
| SLC25A46 | 0.427 | 3.80E-25 |
| ANGPTL1 | 0.458 | 4.13E-29 |
| CD27 | 0.609 | 1.32E-55 |
| DMXL2 | 0.422 | 1.65E-24 |
| TMEM229B | 0.453 | 2.01E-28 |
| ARL14EP | 0.454 | 1.66E-28 |
| SAMD9L | 0.579 | 3.25E-49 |
| IL18RAP | 0.719 | 2.11E-86 |
| APOL6 | 0.58 | 1.93E-49 |
| PPP1R16B | 0.732 | 8.39E-91 |
| PIK3R5 | 0.587 | 6.86E-51 |
| POLK | 0.428 | 3.19E-25 |
| GZMA | 0.719 | 2.55E-86 |
| ALDOA | -0.414 | 1.49E-23 |
| PCNX1 | 0.454 | 1.37E-28 |
| FCMR | 0.69 | 9.12E-77 |
| CLEC2D | 0.719 | 2.83E-86 |
| MOXD1 | 0.455 | 1.16E-28 |
| SYK | 0.406 | 1.29E-22 |
| CLEC10A | 0.625 | 3.09E-59 |
| RTN1 | 0.489 | 1.65E-33 |
| ZNF791 | 0.414 | 1.64E-23 |
| GIMAP1 | 0.643 | 9.21E-64 |
| TENT5C | 0.486 | 4.73E-33 |
| CORO1A | 0.495 | 2.18E-34 |
| FDCSP | 0.493 | 3.93E-34 |
| ST8SIA4 | 0.469 | 1.37E-30 |
| NLRC4 | 0.459 | 3.54E-29 |
| NPL | 0.53 | 4.76E-40 |
| LILRB4 | 0.515 | 1.48E-37 |
| ARHGAP25 | 0.736 | 1.85E-92 |
| BTN3A2 | 0.503 | 1.13E-35 |
| PTGDS | 0.457 | 5.01E-29 |
| TMC8 | 0.478 | 6.99E-32 |
| FCGR1A | 0.453 | 1.79E-28 |
| PDE5A | 0.426 | 5.01E-25 |
| PXK | 0.464 | 5.64E-30 |
| GIMAP4 | 0.765 | 5.33E-104 |
| NABP1 | 0.441 | 7.86E-27 |
| CSGALNACT2 | 0.428 | 3.31E-25 |
| HIVEP3 | 0.412 | 2.44E-23 |
| CD40 | 0.42 | 3.02E-24 |
| OSBPL8 | 0.428 | 3.21E-25 |
| FGD3 | 0.549 | 2.25E-43 |
| HLA-DOB | 0.578 | 6.02E-49 |
| KLHL6 | 0.708 | 2.11E-82 |
| IL2RB | 0.639 | 1.13E-62 |
| NIN | 0.453 | 1.76E-28 |
| RASGRP1 | 0.518 | 5.40E-38 |
| CCR8 | 0.645 | 2.91E-64 |
| GNB4 | 0.469 | 1.28E-30 |
| EVL | 0.443 | 3.77E-27 |
| DAPP1 | 0.542 | 3.54E-42 |
| FCRL5 | 0.577 | 9.87E-49 |
| CMKLR1 | 0.594 | 3.05E-52 |
| TPK1 | 0.444 | 2.79E-27 |
| LPAR6 | 0.561 | 1.37E-45 |
| JAZF1 | 0.487 | 3.44E-33 |
| IKZF3 | 0.751 | 3.91E-98 |
| CRBN | 0.451 | 3.14E-28 |
| CHIC1 | 0.404 | 1.78E-22 |
| SH2B3 | 0.51 | 1.02E-36 |
| HLA-DRA | 0.515 | 1.54E-37 |
| BTN2A2 | 0.576 | 1.20E-48 |
| TRPV2 | 0.422 | 1.62E-24 |
| APOBEC3G | 0.653 | 1.94E-66 |
| HCST | 0.43 | 1.76E-25 |
| LAT2 | 0.442 | 4.90E-27 |
| MEF2C | 0.485 | 5.50E-33 |
| CYTH4 | 0.626 | 1.42E-59 |
| TESPA1 | 0.79 | 2.33E-115 |
| IDO1 | 0.448 | 1.04E-27 |
| ARHGAP9 | 0.694 | 3.49E-78 |
| FOXP3 | 0.518 | 4.53E-38 |
| WIPF1 | 0.721 | 6.34E-87 |
| FMNL3 | 0.583 | 5.61E-50 |
| KPNA5 | 0.434 | 6.00E-26 |
| TSPAN32 | 0.452 | 2.97E-28 |
| SELPLG | 0.474 | 2.77E-31 |
| MIF | -0.418 | 4.66E-24 |
| AKNA | 0.605 | 1.28E-54 |
| ICOS | 0.861 | 3.02E-158 |
| WASHC4 | 0.407 | 8.90E-23 |
| PLA2G2D | 0.663 | 4.26E-69 |
| CSF2RA | 0.456 | 7.70E-29 |
| HTRA4 | 0.456 | 8.96E-29 |
| AMPD3 | 0.412 | 2.32E-23 |
| SEMA4D | 0.535 | 5.05E-41 |
| RASSF2 | 0.6 | 1.21E-53 |
| CDK17 | 0.41 | 4.20E-23 |
| CD83 | 0.439 | 1.33E-26 |
| SIGLEC14 | 0.447 | 1.27E-27 |
| KMO | 0.488 | 2.34E-33 |
| GIT2 | 0.538 | 1.52E-41 |
| VAV1 | 0.434 | 5.88E-26 |
| TNFSF8 | 0.774 | 9.26E-108 |
| HLA-F | 0.449 | 6.27E-28 |
| IL15RA | 0.434 | 5.14E-26 |
| CD96 | 0.769 | 8.62E-106 |
| NLRC3 | 0.793 | 1.19E-116 |
| CDC14A | 0.497 | 1.16E-34 |
| ITGAL | 0.71 | 2.60E-83 |
| SNAI3 | 0.401 | 4.20E-22 |
| RAP1A | 0.434 | 5.48E-26 |
| HPS5 | 0.457 | 5.30E-29 |
| ITK | 0.914 | 4.38E-211 |
| KCTD12 | 0.464 | 7.49E-30 |
| GPR141 | 0.571 | 1.59E-47 |
| CD52 | 0.527 | 1.65E-39 |
| SNRK | 0.484 | 7.88E-33 |
| PTK2B | 0.403 | 2.76E-22 |
| ZNF438 | 0.401 | 4.28E-22 |
| SH2D1A | 0.941 | 1.99E-253 |
| LTB | 0.463 | 8.44E-30 |
| IFNG | 0.66 | 2.45E-68 |
| LAG3 | 0.448 | 9.73E-28 |
| COL6A6 | 0.446 | 1.76E-27 |
| CLEC4E | 0.497 | 1.18E-34 |
| DENND4A | 0.451 | 3.19E-28 |
| GIMAP2 | 0.695 | 2.03E-78 |
| RAB30 | 0.561 | 1.28E-45 |
| STAT1 | 0.559 | 2.40E-45 |
| CD200 | 0.41 | 3.99E-23 |
| TNFAIP8 | 0.624 | 4.90E-59 |
| CD3G | 0.873 | 2.38E-168 |
| PTPRO | 0.465 | 4.46E-30 |
| TCL1A | 0.499 | 5.65E-35 |
| GPR65 | 0.781 | 3.07E-111 |
| PHF11 | 0.443 | 3.75E-27 |
| CD19 | 0.573 | 5.70E-48 |
| PPM1K | 0.514 | 1.93E-37 |
| ADGRG5 | 0.557 | 6.35E-45 |
| GNGT2 | 0.432 | 9.57E-26 |
| MS4A6A | 0.64 | 6.41E-63 |
| TMEM140 | 0.479 | 4.69E-32 |
| DNAJC24 | 0.401 | 4.16E-22 |
| MOB1A | 0.473 | 3.45E-31 |
| JUP | -0.444 | 3.39E-27 |
| PARP14 | 0.444 | 3.32E-27 |
| PLA2G7 | 0.523 | 6.28E-39 |
| UBD | 0.573 | 5.17E-48 |
| AP1S2 | 0.541 | 6.05E-42 |
| IL12RB1 | 0.702 | 1.43E-80 |
| TLR10 | 0.672 | 1.86E-71 |
| COASY | -0.409 | 5.33E-23 |
| MFNG | 0.434 | 5.04E-26 |
| DOCK10 | 0.653 | 3.02E-66 |
| LILRB2 | 0.464 | 5.72E-30 |
| LCK | 0.751 | 3.52E-98 |
| AC136428.1 | 0.405 | 1.41E-22 |
| DTHD1 | 0.425 | 8.05E-25 |
| CSF2RB | 0.659 | 4.74E-68 |
| CYBB | 0.626 | 1.88E-59 |
| WAS | 0.528 | 8.59E-40 |
| ZBP1 | 0.58 | 2.51E-49 |
| SLFN12L | 0.75 | 8.91E-98 |
| MBNL1 | 0.453 | 2.17E-28 |
| TMEM273 | 0.617 | 2.53E-57 |
| SLAMF6 | 0.843 | 1.27E-145 |
| SIGLEC10 | 0.564 | 2.78E-46 |
| SLC39A7 | -0.411 | 3.50E-23 |
| KLF12 | 0.515 | 1.27E-37 |
| MS4A1 | 0.692 | 2.40E-77 |
| OXNAD1 | 0.506 | 4.78E-36 |
| IL7R | 0.736 | 2.66E-92 |
| EIF4E3 | 0.442 | 4.78E-27 |
| AOAH | 0.771 | 1.16E-106 |
| JCHAIN | 0.569 | 3.52E-47 |
| CD3E | 0.765 | 4.69E-104 |
| SP140 | 0.753 | 9.39E-99 |
| UBE2L6 | 0.422 | 1.62E-24 |
| MSR1 | 0.419 | 3.99E-24 |
| SH2D2A | 0.441 | 6.90E-27 |
| SSPN | 0.421 | 1.89E-24 |
| NLRP3 | 0.54 | 8.59E-42 |
| PAG1 | 0.426 | 4.88E-25 |
| BCL11B | 0.699 | 1.64E-79 |
| HACD4 | 0.41 | 4.47E-23 |
| ARHGAP30 | 0.621 | 1.91E-58 |
| B2M | 0.588 | 4.37E-51 |
| BTK | 0.67 | 4.43E-71 |
| RHOH | 0.702 | 1.49E-80 |
| C1orf54 | 0.48 | 3.91E-32 |
| TBC1D4 | 0.462 | 1.17E-29 |
| TNFRSF1B | 0.455 | 9.68E-29 |
| GMFG | 0.578 | 4.93E-49 |
| GPR132 | 0.415 | 1.16E-23 |
| GZMK | 0.836 | 8.28E-141 |
| NFKBIL1 | -0.411 | 3.51E-23 |
| JAML | 0.599 | 1.96E-53 |
| GP1BA | 0.511 | 7.51E-37 |
| APOL4 | 0.408 | 6.32E-23 |
| N4BP2L1 | 0.619 | 8.01E-58 |
| CFLAR | 0.449 | 7.66E-28 |
| IL2RA | 0.571 | 1.20E-47 |
| TMOD2 | 0.441 | 7.60E-27 |
| DNAJC5B | 0.508 | 1.74E-36 |
| FYN | 0.493 | 3.62E-34 |
| PIK3CD | 0.501 | 2.25E-35 |
| FYB1 | 0.745 | 7.45E-96 |
| TRAF3IP3 | 0.842 | 2.97E-145 |
| KIAA1551 | 0.58 | 1.75E-49 |
| KLRG1 | 0.775 | 3.37E-108 |
| ZNF441 | 0.441 | 8.13E-27 |
| SPCS3 | 0.404 | 2.15E-22 |
| FCGR2B | 0.506 | 4.24E-36 |
| B4GALT2 | -0.409 | 4.88E-23 |
| MORC3 | 0.422 | 1.62E-24 |
| GPR18 | 0.751 | 2.99E-98 |
| BCL2 | 0.436 | 3.06E-26 |
| CEP120 | 0.467 | 2.77E-30 |
| RUNX3 | 0.597 | 6.49E-53 |
| CLEC12A | 0.486 | 4.37E-33 |
| CD28 | 0.815 | 3.64E-128 |
| LAPTM5 | 0.55 | 1.04E-43 |
| LRRN3 | 0.418 | 4.36E-24 |
| IL2RG | 0.639 | 8.24E-63 |
| TFEC | 0.754 | 3.26E-99 |
| HLA-DOA | 0.554 | 2.06E-44 |
| GPR15 | 0.408 | 7.87E-23 |
| PNRC1 | 0.529 | 5.86E-40 |
| GPR155 | 0.565 | 1.60E-46 |
| C16orf54 | 0.609 | 1.66E-55 |
| PDE7A | 0.404 | 1.98E-22 |
| P2RX1 | 0.433 | 8.34E-26 |
| DENND2D | 0.402 | 3.43E-22 |
| ANKRD44 | 0.693 | 7.85E-78 |
| LILRA4 | 0.556 | 8.24E-45 |
| RAPGEF6 | 0.446 | 1.75E-27 |
| PLCG2 | 0.589 | 3.50E-51 |
| PRDM1 | 0.591 | 8.83E-52 |
| GBP4 | 0.696 | 1.23E-78 |
| P2RY12 | 0.578 | 4.45E-49 |
| TRAT1 | 1 | 0 |
| ZNF671 | 0.416 | 9.16E-24 |
| ITPR2 | 0.417 | 5.90E-24 |
| MAP4K1 | 0.667 | 3.28E-70 |
| GFI1 | 0.748 | 5.69E-97 |
| TBX21 | 0.551 | 7.42E-44 |
| APOL3 | 0.63 | 1.32E-60 |
| C7 | 0.418 | 4.37E-24 |
| GPR183 | 0.654 | 1.71E-66 |
| GZMH | 0.604 | 1.56E-54 |
| LRRK1 | 0.447 | 1.24E-27 |
| TRANK1 | 0.511 | 6.99E-37 |
| RIC1 | 0.48 | 3.08E-32 |
| HVCN1 | 0.566 | 1.26E-46 |
| CIITA | 0.571 | 1.18E-47 |
| POU2F2 | 0.514 | 1.72E-37 |
| MFSD1 | 0.481 | 2.70E-32 |
| SLAMF1 | 0.844 | 2.21E-146 |
| NXPE3 | 0.431 | 1.46E-25 |
| MEI1 | 0.509 | 1.33E-36 |
| SLC1A3 | 0.571 | 1.03E-47 |
| SAMD9 | 0.417 | 7.10E-24 |
| ATP6V1B2 | 0.444 | 3.25E-27 |
| P2RY13 | 0.675 | 2.44E-72 |
| CD5 | 0.726 | 7.15E-89 |
| MCAT | -0.42 | 2.48E-24 |
| FGL2 | 0.686 | 1.34E-75 |
| HECA | 0.46 | 2.48E-29 |
| PIK3AP1 | 0.617 | 2.41E-57 |
| SETDB2 | 0.483 | 1.42E-32 |
| MS4A7 | 0.543 | 1.96E-42 |
| GPR34 | 0.564 | 3.18E-46 |
| CD163 | 0.447 | 1.41E-27 |
| ATG7 | 0.464 | 7.43E-30 |
| CD180 | 0.607 | 3.63E-55 |

**Table S2**. Roles of strongly co-expressed genes of TRAT1.

| Type | ID | Description | p | Count |
| --- | --- | --- | --- | --- |
| BP | GO:0042110 | T cell activation | 1.02965E-78 | 104 |
| BP | GO:0030098 | lymphocyte differentiation | 5.21324E-64 | 83 |
| BP | GO:0051249 | regulation of lymphocyte activation | 1.36414E-56 | 87 |
| BP | GO:0050863 | regulation of T cell activation | 1.01704E-55 | 73 |
| BP | GO:0030217 | T cell differentiation | 7.41289E-53 | 64 |
| BP | GO:0007159 | leukocyte cell-cell adhesion | 5.68667E-51 | 71 |
| BP | GO:1903037 | regulation of leukocyte cell-cell adhesion | 3.18182E-48 | 66 |
| BP | GO:0046651 | lymphocyte proliferation | 1.94101E-45 | 61 |
| BP | GO:0032943 | mononuclear cell proliferation | 3.10399E-45 | 61 |
| BP | GO:0022407 | regulation of cell-cell adhesion | 2.89039E-44 | 70 |
| BP | GO:0070661 | leukocyte proliferation | 4.4185E-44 | 62 |
| BP | GO:1903039 | positive regulation of leukocyte cell-cell adhesion | 1.07771E-42 | 54 |
| BP | GO:0050870 | positive regulation of T cell activation | 4.67774E-42 | 52 |
| BP | GO:0022409 | positive regulation of cell-cell adhesion | 3.60027E-41 | 56 |
| BP | GO:0050670 | regulation of lymphocyte proliferation | 7.48115E-39 | 50 |
| BP | GO:0032944 | regulation of mononuclear cell proliferation | 9.65744E-39 | 50 |
| BP | GO:0050867 | positive regulation of cell activation | 1.39791E-38 | 64 |
| BP | GO:0070663 | regulation of leukocyte proliferation | 1.47203E-38 | 51 |
| BP | GO:0051251 | positive regulation of lymphocyte activation | 1.1153E-37 | 59 |
| BP | GO:0002696 | positive regulation of leukocyte activation | 1.74694E-37 | 62 |
| BP | GO:0045785 | positive regulation of cell adhesion | 5.99573E-37 | 63 |
| BP | GO:0050851 | antigen receptor-mediated signaling pathway | 6.41104E-37 | 57 |
| BP | GO:0046631 | alpha-beta T cell activation | 4.47199E-36 | 41 |
| BP | GO:0002429 | immune response-activating cell surface receptor signaling pathway | 1.36734E-35 | 66 |
| BP | GO:0042098 | T cell proliferation | 6.28577E-33 | 43 |
| BP | GO:0045619 | regulation of lymphocyte differentiation | 3.88715E-32 | 41 |
| BP | GO:0002683 | negative regulation of immune system process | 1.91102E-31 | 61 |
| BP | GO:1902105 | regulation of leukocyte differentiation | 1.10278E-30 | 48 |
| BP | GO:0001819 | positive regulation of cytokine production | 1.78426E-30 | 60 |
| BP | GO:0042129 | regulation of T cell proliferation | 6.46046E-30 | 38 |
| BP | GO:0002285 | lymphocyte activation involved in immune response | 1.15484E-29 | 40 |
| BP | GO:0045580 | regulation of T cell differentiation | 2.03674E-29 | 36 |
| BP | GO:0050671 | positive regulation of lymphocyte proliferation | 2.88626E-29 | 35 |
| BP | GO:0032946 | positive regulation of mononuclear cell proliferation | 3.86576E-29 | 35 |
| BP | GO:0070665 | positive regulation of leukocyte proliferation | 3.63918E-28 | 35 |
| BP | GO:0042113 | B cell activation | 5.19724E-28 | 48 |
| BP | GO:0002460 | adaptive immune response based on somatic recombination of  immune receptors built from immunoglobulin superfamily domains | 8.73085E-28 | 51 |
| BP | GO:0032609 | interferon-gamma production | 1.29798E-27 | 32 |
| BP | GO:0042102 | positive regulation of T cell proliferation | 1.53095E-27 | 30 |
| BP | GO:0046632 | alpha-beta T cell differentiation | 1.21021E-26 | 30 |
| BP | GO:1903706 | regulation of hemopoiesis | 2.19591E-26 | 56 |
| BP | GO:0050852 | T cell receptor signaling pathway | 1.67902E-25 | 38 |
| BP | GO:0002697 | regulation of immune effector process | 1.33002E-24 | 53 |
| BP | GO:0045058 | T cell selection | 2.09681E-23 | 21 |
| BP | GO:0002695 | negative regulation of leukocyte activation | 2.12138E-23 | 34 |
| BP | GO:0050866 | negative regulation of cell activation | 1.52434E-22 | 35 |
| BP | GO:0002819 | regulation of adaptive immune response | 1.70717E-22 | 32 |
| BP | GO:0051250 | negative regulation of lymphocyte activation | 1.62148E-21 | 30 |
| BP | GO:0032649 | regulation of interferon-gamma production | 1.79544E-21 | 26 |
| BP | GO:0050854 | regulation of antigen receptor-mediated signaling pathway | 2.89343E-21 | 22 |
| BP | GO:0050900 | leukocyte migration | 3.02608E-21 | 51 |
| BP | GO:0046634 | regulation of alpha-beta T cell activation | 3.35511E-21 | 25 |
| BP | GO:0002449 | lymphocyte mediated immunity | 5.28037E-21 | 43 |
| BP | GO:0042100 | B cell proliferation | 5.97272E-21 | 25 |
| BP | GO:0002822 | regulation of adaptive immune response based on somatic  recombination of immune receptors built from  immunoglobulin superfamily domains | 1.72419E-20 | 29 |
| BP | GO:0002706 | regulation of lymphocyte mediated immunity | 3.83839E-20 | 29 |
| BP | GO:0045621 | positive regulation of lymphocyte differentiation | 8.04058E-20 | 24 |
| BP | GO:0002286 | T cell activation involved in immune response | 1.11206E-19 | 25 |
| BP | GO:0002703 | regulation of leukocyte mediated immunity | 2.39986E-19 | 32 |
| BP | GO:0045088 | regulation of innate immune response | 3.72288E-19 | 46 |
| BP | GO:0043368 | positive T cell selection | 1.39558E-18 | 16 |
| BP | GO:0032729 | positive regulation of interferon-gamma production | 1.67274E-18 | 20 |
| BP | GO:1902107 | positive regulation of leukocyte differentiation | 2.15236E-18 | 27 |
| BP | GO:0042108 | positive regulation of cytokine biosynthetic process | 3.29388E-18 | 20 |
| BP | GO:0002237 | response to molecule of bacterial origin | 4.91842E-18 | 39 |
| BP | GO:0050663 | cytokine secretion | 6.46621E-18 | 33 |
| BP | GO:0042035 | regulation of cytokine biosynthetic process | 1.03812E-17 | 24 |
| BP | GO:0048872 | homeostasis of number of cells | 1.38414E-17 | 33 |
| BP | GO:0050864 | regulation of B cell activation | 1.62064E-17 | 29 |
| BP | GO:0050853 | B cell receptor signaling pathway | 1.75738E-17 | 25 |
| BP | GO:1903708 | positive regulation of hemopoiesis | 1.88597E-17 | 29 |
| BP | GO:0007204 | positive regulation of cytosolic calcium ion concentration | 1.89913E-17 | 37 |
| BP | GO:0045582 | positive regulation of T cell differentiation | 2.07062E-17 | 21 |
| BP | GO:0030183 | B cell differentiation | 2.58901E-17 | 25 |
| BP | GO:0043367 | CD4-positive, alpha-beta T cell differentiation | 2.93069E-17 | 20 |
| BP | GO:0042089 | cytokine biosynthetic process | 6.65434E-17 | 24 |
| BP | GO:0072676 | lymphocyte migration | 7.3293E-17 | 23 |
| BP | GO:0001818 | negative regulation of cytokine production | 7.96378E-17 | 35 |
| BP | GO:0042107 | cytokine metabolic process | 8.09645E-17 | 24 |
| BP | GO:0050868 | negative regulation of T cell activation | 9.05013E-17 | 23 |
| BP | GO:0035710 | CD4-positive, alpha-beta T cell activation | 2.03623E-16 | 21 |
| BP | GO:0030888 | regulation of B cell proliferation | 2.0502E-16 | 18 |
| BP | GO:0046635 | positive regulation of alpha-beta T cell activation | 2.83464E-16 | 18 |
| BP | GO:0032496 | response to lipopolysaccharide | 3.7799E-16 | 36 |
| BP | GO:0050777 | negative regulation of immune response | 7.41762E-16 | 25 |
| BP | GO:0051480 | regulation of cytosolic calcium ion concentration | 7.62349E-16 | 37 |
| BP | GO:0031295 | T cell costimulation | 8.30818E-16 | 17 |
| BP | GO:0031294 | lymphocyte costimulation | 1.16169E-15 | 17 |
| BP | GO:0060326 | cell chemotaxis | 1.22483E-15 | 34 |
| BP | GO:0002699 | positive regulation of immune effector process | 1.33011E-15 | 29 |
| BP | GO:0002292 | T cell differentiation involved in immune response | 1.74245E-15 | 18 |
| BP | GO:1903038 | negative regulation of leukocyte cell-cell adhesion | 2.37862E-15 | 23 |
| BP | GO:0030595 | leukocyte chemotaxis | 3.53366E-15 | 29 |
| BP | GO:0002287 | alpha-beta T cell activation involved in immune response | 4.13969E-15 | 17 |
| BP | GO:0002293 | alpha-beta T cell differentiation involved in immune response | 4.13969E-15 | 17 |
| BP | GO:0032623 | interleukin-2 production | 5.59649E-15 | 17 |
| BP | GO:0071219 | cellular response to molecule of bacterial origin | 6.39494E-15 | 28 |
| BP | GO:0022408 | negative regulation of cell-cell adhesion | 7.89458E-15 | 26 |
| BP | GO:1990868 | response to chemokine | 8.75796E-15 | 20 |
| BP | GO:1990869 | cellular response to chemokine | 8.75796E-15 | 20 |
| BP | GO:0032663 | regulation of interleukin-2 production | 9.14609E-15 | 16 |
| BP | GO:0001776 | leukocyte homeostasis | 1.07314E-14 | 19 |
| BP | GO:0007162 | negative regulation of cell adhesion | 1.16322E-14 | 32 |
| BP | GO:0072678 | T cell migration | 1.33524E-14 | 17 |
| BP | GO:0071216 | cellular response to biotic stimulus | 1.41471E-14 | 29 |
| BP | GO:0002824 | positive regulation of adaptive immune response based  on somatic recombination of immune receptors built  from immunoglobulin superfamily domains | 1.62827E-14 | 20 |
| BP | GO:0071887 | leukocyte apoptotic process | 3.59662E-14 | 20 |
| BP | GO:0050707 | regulation of cytokine secretion | 3.85924E-14 | 27 |
| BP | GO:0055074 | calcium ion homeostasis | 4.14785E-14 | 40 |
| BP | GO:0002821 | positive regulation of adaptive immune response | 4.35936E-14 | 20 |
| BP | GO:0032103 | positive regulation of response to external stimulus | 4.52713E-14 | 33 |
| BP | GO:1903555 | regulation of tumor necrosis factor superfamily  cytokine production | 4.99242E-14 | 24 |
| BP | GO:0002456 | T cell mediated immunity | 5.27216E-14 | 20 |
| BP | GO:0002294 | CD4-positive, alpha-beta T cell differentiation  involved in immune response | 5.79E-14 | 16 |
| BP | GO:0071346 | cellular response to interferon-gamma | 5.8069E-14 | 25 |
| BP | GO:2000106 | regulation of leukocyte apoptotic process | 7.69128E-14 | 18 |
| BP | GO:0034341 | response to interferon-gamma | 7.89506E-14 | 26 |
| BP | GO:0006874 | cellular calcium ion homeostasis | 8.08633E-14 | 39 |
| BP | GO:0019722 | calcium-mediated signaling | 9.73165E-14 | 27 |
| BP | GO:0071706 | tumor necrosis factor superfamily cytokine production | 9.91416E-14 | 24 |
| BP | GO:0002312 | B cell activation involved in immune response | 1.08578E-13 | 17 |
| BP | GO:0046637 | regulation of alpha-beta T cell differentiation | 1.3397E-13 | 16 |
| BP | GO:0031341 | regulation of cell killing | 1.35566E-13 | 19 |
| BP | GO:0071222 | cellular response to lipopolysaccharide | 1.60729E-13 | 26 |
| BP | GO:0045089 | positive regulation of innate immune response | 1.79495E-13 | 35 |
| BP | GO:0050855 | regulation of B cell receptor signaling pathway | 2.06891E-13 | 12 |
| BP | GO:0070098 | chemokine-mediated signaling pathway | 2.26434E-13 | 18 |
| BP | GO:0097529 | myeloid leukocyte migration | 2.85049E-13 | 26 |
| BP | GO:0032680 | regulation of tumor necrosis factor production | 2.86303E-13 | 23 |
| BP | GO:0001910 | regulation of leukocyte mediated cytotoxicity | 3.50469E-13 | 17 |
| BP | GO:0070664 | negative regulation of leukocyte proliferation | 3.50469E-13 | 17 |
| BP | GO:0002685 | regulation of leukocyte migration | 4.18397E-13 | 25 |
| BP | GO:0032640 | tumor necrosis factor production | 4.28165E-13 | 23 |
| BP | GO:0002708 | positive regulation of lymphocyte mediated immunity | 5.02836E-13 | 19 |
| BP | GO:0042093 | T-helper cell differentiation | 5.876E-13 | 15 |
| BP | GO:0001909 | leukocyte mediated cytotoxicity | 7.17192E-13 | 19 |
| BP | GO:0072503 | cellular divalent inorganic cation homeostasis | 8.37316E-13 | 39 |
| BP | GO:0032633 | interleukin-4 production | 1.3123E-12 | 12 |
| BP | GO:0097530 | granulocyte migration | 1.55881E-12 | 21 |
| BP | GO:0032945 | negative regulation of mononuclear cell proliferation | 1.59518E-12 | 16 |
| BP | GO:0050672 | negative regulation of lymphocyte proliferation | 1.59518E-12 | 16 |
| BP | GO:0009615 | response to virus | 1.85694E-12 | 31 |
| BP | GO:0002440 | production of molecular mediator of immune response | 2.00101E-12 | 29 |
| BP | GO:0050727 | regulation of inflammatory response | 2.24504E-12 | 38 |
| BP | GO:0032655 | regulation of interleukin-12 production | 3.38899E-12 | 14 |
| BP | GO:1990266 | neutrophil migration | 4.41869E-12 | 19 |
| BP | GO:0032615 | interleukin-12 production | 5.83725E-12 | 14 |
| BP | GO:0002698 | negative regulation of immune effector process | 6.01482E-12 | 19 |
| BP | GO:0043370 | regulation of CD4-positive, alpha-beta T cell differentiation | 8.19878E-12 | 13 |
| BP | GO:0002700 | regulation of production of molecular mediator  of immune response | 1.02751E-11 | 20 |
| BP | GO:0032743 | positive regulation of interleukin-2 production | 1.03988E-11 | 11 |
| BP | GO:0050869 | negative regulation of B cell activation | 1.03988E-11 | 11 |
| BP | GO:0002709 | regulation of T cell mediated immunity | 1.1358E-11 | 15 |
| BP | GO:0032753 | positive regulation of interleukin-4 production | 1.20425E-11 | 10 |
| BP | GO:0002704 | negative regulation of leukocyte mediated immunity | 1.47394E-11 | 13 |
| BP | GO:2000514 | regulation of CD4-positive, alpha-beta T cell activation | 2.06021E-11 | 14 |
| BP | GO:0002260 | lymphocyte homeostasis | 2.61141E-11 | 14 |
| BP | GO:0002335 | mature B cell differentiation | 3.31533E-11 | 10 |
| BP | GO:0045086 | positive regulation of interleukin-2 biosynthetic process | 3.80659E-11 | 8 |
| BP | GO:0002705 | positive regulation of leukocyte mediated immunity | 3.87259E-11 | 19 |
| BP | GO:0030593 | neutrophil chemotaxis | 4.69031E-11 | 17 |
| BP | GO:0019932 | second-messenger-mediated signaling | 4.706E-11 | 34 |
| BP | GO:0030099 | myeloid cell differentiation | 4.88783E-11 | 33 |
| BP | GO:0045066 | regulatory T cell differentiation | 5.0544E-11 | 11 |
| BP | GO:0045076 | regulation of interleukin-2 biosynthetic process | 5.06913E-11 | 9 |
| BP | GO:0032673 | regulation of interleukin-4 production | 8.24476E-11 | 10 |
| BP | GO:0036336 | dendritic cell migration | 8.24476E-11 | 10 |
| BP | GO:0071621 | granulocyte chemotaxis | 8.45875E-11 | 18 |
| BP | GO:0031343 | positive regulation of cell killing | 9.88348E-11 | 14 |
| BP | GO:0070227 | lymphocyte apoptotic process | 1.21678E-10 | 14 |
| BP | GO:0010818 | T cell chemotaxis | 1.25894E-10 | 10 |
| BP | GO:0050858 | negative regulation of antigen receptor-mediated  signaling pathway | 1.25894E-10 | 10 |
| BP | GO:0033077 | T cell differentiation in thymus | 1.49258E-10 | 14 |
| BP | GO:0035590 | purinergic nucleotide receptor signaling pathway | 1.55457E-10 | 9 |
| BP | GO:0051607 | defense response to virus | 1.89703E-10 | 24 |
| BP | GO:0042094 | interleukin-2 biosynthetic process | 2.5828E-10 | 9 |
| BP | GO:0050710 | negative regulation of cytokine secretion | 3.2662E-10 | 14 |
| BP | GO:0001906 | cell killing | 3.3951E-10 | 20 |
| BP | GO:2000401 | regulation of lymphocyte migration | 4.51628E-10 | 13 |
| BP | GO:0043087 | regulation of GTPase activity | 4.79662E-10 | 34 |
| BP | GO:0060759 | regulation of response to cytokine stimulus | 4.91476E-10 | 21 |
| BP | GO:0045589 | regulation of regulatory T cell differentiation | 5.74636E-10 | 10 |
| BP | GO:0060333 | interferon-gamma-mediated signaling pathway | 5.80619E-10 | 15 |
| BP | GO:0002407 | dendritic cell chemotaxis | 6.54361E-10 | 9 |
| BP | GO:0050857 | positive regulation of antigen receptor-mediated  signaling pathway | 6.54361E-10 | 9 |
| BP | GO:0006968 | cellular defense response | 6.78313E-10 | 12 |
| BP | GO:0032715 | negative regulation of interleukin-6 production | 6.78313E-10 | 12 |
| BP | GO:0070228 | regulation of lymphocyte apoptotic process | 6.78313E-10 | 12 |
| BP | GO:0046633 | alpha-beta T cell proliferation | 8.09332E-10 | 10 |
| BP | GO:0042088 | T-helper 1 type immune response | 8.59167E-10 | 11 |
| BP | GO:0001959 | regulation of cytokine-mediated signaling pathway | 8.64973E-10 | 20 |
| BP | GO:0050920 | regulation of chemotaxis | 9.93778E-10 | 22 |
| BP | GO:0043383 | negative T cell selection | 1.14886E-09 | 7 |
| BP | GO:0031348 | negative regulation of defense response | 1.14956E-09 | 23 |
| BP | GO:0002687 | positive regulation of leukocyte migration | 1.33286E-09 | 17 |
| BP | GO:0001912 | positive regulation of leukocyte mediated cytotoxicity | 1.34218E-09 | 12 |
| BP | GO:0002360 | T cell lineage commitment | 1.50721E-09 | 9 |
| BP | GO:0002688 | regulation of leukocyte chemotaxis | 1.79679E-09 | 16 |
| BP | GO:0043547 | positive regulation of GTPase activity | 1.95305E-09 | 30 |
| BP | GO:0032612 | interleukin-1 production | 2.0481E-09 | 16 |
| BP | GO:2000516 | positive regulation of CD4-positive, alpha-beta T cell activation | 2.10233E-09 | 10 |
| BP | GO:0046638 | positive regulation of alpha-beta T cell differentiation | 2.41466E-09 | 11 |
| BP | GO:2000107 | negative regulation of leukocyte apoptotic process | 2.41466E-09 | 11 |
| BP | GO:0035589 | G protein-coupled purinergic nucleotide receptor  signaling pathway | 2.44479E-09 | 7 |
| BP | GO:0032732 | positive regulation of interleukin-1 production | 2.54397E-09 | 12 |
| BP | GO:0042130 | negative regulation of T cell proliferation | 2.54397E-09 | 12 |
| BP | GO:0030101 | natural killer cell activation | 2.59304E-09 | 14 |
| BP | GO:0050921 | positive regulation of chemotaxis | 3.06537E-09 | 17 |
| BP | GO:0045577 | regulation of B cell differentiation | 3.21135E-09 | 9 |
| BP | GO:0043369 | CD4-positive or CD8-positive, alpha-beta T cell  lineage commitment | 3.27928E-09 | 8 |
| BP | GO:0032652 | regulation of interleukin-1 production | 3.44698E-09 | 15 |
| BP | GO:1903557 | positive regulation of tumor necrosis factor superfamily  cytokine production | 3.53917E-09 | 14 |
| BP | GO:0002707 | negative regulation of lymphocyte mediated immunity | 3.76763E-09 | 10 |
| BP | GO:0001771 | immunological synapse formation | 4.80236E-09 | 7 |
| BP | GO:0050715 | positive regulation of cytokine secretion | 4.81821E-09 | 17 |
| BP | GO:0050708 | regulation of protein secretion | 4.88029E-09 | 32 |
| BP | GO:0050856 | regulation of T cell receptor signaling pathway | 4.9738E-09 | 10 |
| BP | GO:0045061 | thymic T cell selection | 5.20164E-09 | 8 |
| BP | GO:0002791 | regulation of peptide secretion | 5.40131E-09 | 33 |
| BP | GO:0043372 | positive regulation of CD4-positive, alpha-beta  T cell differentiation | 6.41179E-09 | 9 |
| BP | GO:0048247 | lymphocyte chemotaxis | 6.7966E-09 | 12 |
| BP | GO:0032653 | regulation of interleukin-10 production | 7.62936E-09 | 11 |
| BP | GO:0032731 | positive regulation of interleukin-1 beta production | 7.62936E-09 | 11 |
| BP | GO:0032479 | regulation of type I interferon production | 7.92779E-09 | 16 |
| BP | GO:0030890 | positive regulation of B cell proliferation | 8.45199E-09 | 10 |
| BP | GO:0032606 | type I interferon production | 9.98188E-09 | 16 |
| BP | GO:0002224 | toll-like receptor signaling pathway | 1.02377E-08 | 17 |
| BP | GO:0019882 | antigen processing and presentation | 1.14593E-08 | 21 |
| BP | GO:0036037 | CD8-positive, alpha-beta T cell activation | 1.2085E-08 | 8 |
| BP | GO:0035587 | purinergic receptor signaling pathway | 1.21162E-08 | 9 |
| BP | GO:0032613 | interleukin-10 production | 1.42918E-08 | 11 |
| BP | GO:0002291 | T cell activation via T cell receptor contact with antigen  bound to MHC molecule on antigen presenting cell | 1.51737E-08 | 6 |
| BP | GO:0032695 | negative regulation of interleukin-12 production | 1.54421E-08 | 7 |
| BP | GO:2000404 | regulation of T cell migration | 1.76953E-08 | 10 |
| BP | GO:0002718 | regulation of cytokine production involved in immune response | 1.82006E-08 | 13 |
| BP | GO:0032720 | negative regulation of tumor necrosis factor production | 1.96797E-08 | 12 |
| BP | GO:0050729 | positive regulation of inflammatory response | 2.08179E-08 | 17 |
| BP | GO:0042533 | tumor necrosis factor biosynthetic process | 2.18394E-08 | 9 |
| BP | GO:0042534 | regulation of tumor necrosis factor biosynthetic process | 2.18394E-08 | 9 |
| BP | GO:0032611 | interleukin-1 beta production | 2.20998E-08 | 14 |
| BP | GO:0002820 | negative regulation of adaptive immune response | 2.23338E-08 | 10 |
| BP | GO:0002377 | immunoglobulin production | 2.26368E-08 | 19 |
| BP | GO:0032760 | positive regulation of tumor necrosis factor production | 2.43809E-08 | 13 |
| BP | GO:0002367 | cytokine production involved in immune response | 2.51382E-08 | 14 |
| BP | GO:0002507 | tolerance induction | 2.57046E-08 | 8 |
| BP | GO:0043373 | CD4-positive, alpha-beta T cell lineage commitment | 2.57837E-08 | 7 |
| BP | GO:1903556 | negative regulation of tumor necrosis factor superfamily  cytokine production | 2.73758E-08 | 12 |
| BP | GO:0002715 | regulation of natural killer cell mediated immunity | 2.80142E-08 | 10 |
| BP | GO:0002690 | positive regulation of leukocyte chemotaxis | 2.81312E-08 | 13 |
| BP | GO:0002701 | negative regulation of production of molecular mediator  of immune response | 2.88638E-08 | 9 |
| BP | GO:0045622 | regulation of T-helper cell differentiation | 2.88638E-08 | 9 |
| BP | GO:0045060 | negative thymic T cell selection | 3.27973E-08 | 6 |
| BP | GO:0060402 | calcium ion transport into cytosol | 3.37235E-08 | 17 |
| BP | GO:0070231 | T cell apoptotic process | 3.4933E-08 | 10 |
| BP | GO:0032651 | regulation of interleukin-1 beta production | 4.27068E-08 | 13 |
| BP | GO:0032635 | interleukin-6 production | 4.4632E-08 | 17 |
| BP | GO:2000403 | positive regulation of lymphocyte migration | 4.90202E-08 | 9 |
| BP | GO:0002218 | activation of innate immune response | 6.29622E-08 | 24 |
| BP | GO:0002363 | alpha-beta T cell lineage commitment | 6.4444E-08 | 7 |
| BP | GO:0046641 | positive regulation of alpha-beta T cell proliferation | 6.4444E-08 | 7 |
| BP | GO:0032647 | regulation of interferon-alpha production | 6.99412E-08 | 8 |
| BP | GO:0002228 | natural killer cell mediated immunity | 7.5119E-08 | 11 |
| BP | GO:0072538 | T-helper 17 type immune response | 9.48439E-08 | 8 |
| BP | GO:0002577 | regulation of antigen processing and presentation | 9.73788E-08 | 7 |
| BP | GO:0042535 | positive regulation of tumor necrosis factor  biosynthetic process | 9.73788E-08 | 7 |
| BP | GO:0002823 | negative regulation of adaptive immune response based  on somatic recombination of immune receptors built  from immunoglobulin superfamily domains | 1.0197E-07 | 9 |
| BP | GO:0060401 | cytosolic calcium ion transport | 1.08494E-07 | 17 |
| BP | GO:0032675 | regulation of interleukin-6 production | 1.16411E-07 | 16 |
| BP | GO:0045059 | positive thymic T cell selection | 1.17589E-07 | 6 |
| BP | GO:0050702 | interleukin-1 beta secretion | 1.17726E-07 | 10 |
| BP | GO:0001782 | B cell homeostasis | 1.27001E-07 | 8 |
| BP | GO:0032607 | interferon-alpha production | 1.27001E-07 | 8 |
| BP | GO:0046640 | regulation of alpha-beta T cell proliferation | 1.27001E-07 | 8 |
| BP | GO:0050690 | regulation of defense response to virus by virus | 1.27001E-07 | 8 |
| BP | GO:0006816 | calcium ion transport | 1.30151E-07 | 28 |
| BP | GO:0002221 | pattern recognition receptor signaling pathway | 1.6623E-07 | 18 |
| BP | GO:2000406 | positive regulation of T cell migration | 1.6809E-07 | 8 |
| BP | GO:0018108 | peptidyl-tyrosine phosphorylation | 1.8339E-07 | 25 |
| BP | GO:0043374 | CD8-positive, alpha-beta T cell differentiation | 2.0218E-07 | 6 |
| BP | GO:0002381 | immunoglobulin production involved in  immunoglobulin mediated immune response | 2.03378E-07 | 10 |
| BP | GO:0072643 | interferon-gamma secretion | 2.06673E-07 | 7 |
| BP | GO:0018212 | peptidyl-tyrosine modification | 2.14326E-07 | 25 |
| BP | GO:0007599 | hemostasis | 2.17891E-07 | 24 |
| BP | GO:0042269 | regulation of natural killer cell mediated cytotoxicity | 2.45654E-07 | 9 |
| BP | GO:0050709 | negative regulation of protein secretion | 2.52363E-07 | 15 |
| BP | GO:0050871 | positive regulation of B cell activation | 2.76904E-07 | 15 |
| BP | GO:0002573 | myeloid leukocyte differentiation | 2.80814E-07 | 18 |
| BP | GO:0001914 | regulation of T cell mediated cytotoxicity | 2.85273E-07 | 8 |
| BP | GO:0070232 | regulation of T cell apoptotic process | 2.85273E-07 | 8 |
| BP | GO:0014066 | regulation of phosphatidylinositol 3-kinase signaling | 3.04098E-07 | 14 |
| BP | GO:0051209 | release of sequestered calcium ion into cytosol | 3.04098E-07 | 14 |
| BP | GO:0002758 | innate immune response-activating signal transduction | 3.11547E-07 | 22 |
| BP | GO:0045824 | negative regulation of innate immune response | 3.39502E-07 | 10 |
| BP | GO:0070838 | divalent metal ion transport | 3.50528E-07 | 29 |
| BP | GO:0032660 | regulation of interleukin-17 production | 3.6633E-07 | 8 |
| BP | GO:0002437 | inflammatory response to antigenic stimulus | 3.68003E-07 | 9 |
| BP | GO:0050706 | regulation of interleukin-1 beta secretion | 3.68003E-07 | 9 |
| BP | GO:0051283 | negative regulation of sequestering of calcium ion | 3.7105E-07 | 14 |
| BP | GO:0097696 | STAT cascade | 3.93027E-07 | 16 |
| BP | GO:0042267 | natural killer cell mediated cytotoxicity | 3.99943E-07 | 10 |
| BP | GO:0035588 | G protein-coupled purinergic receptor signaling pathway | 4.04631E-07 | 7 |
| BP | GO:0033209 | tumor necrosis factor-mediated signaling pathway | 4.26548E-07 | 16 |
| BP | GO:0051282 | regulation of sequestering of calcium ion | 4.50967E-07 | 14 |
| BP | GO:0072511 | divalent inorganic cation transport | 4.5227E-07 | 29 |
| BP | GO:1903531 | negative regulation of secretion by cell | 4.63543E-07 | 18 |
| BP | GO:0045123 | cellular extravasation | 4.69606E-07 | 10 |
| BP | GO:0050701 | interleukin-1 secretion | 4.69606E-07 | 10 |
| BP | GO:0002792 | negative regulation of peptide secretion | 4.75132E-07 | 15 |
| BP | GO:0002295 | T-helper cell lineage commitment | 5.20447E-07 | 6 |
| BP | GO:0030889 | negative regulation of B cell proliferation | 5.20447E-07 | 6 |
| BP | GO:0002711 | positive regulation of T cell mediated immunity | 5.4002E-07 | 9 |
| BP | GO:0051208 | sequestering of calcium ion | 5.99977E-07 | 14 |
| BP | GO:0042116 | macrophage activation | 6.33832E-07 | 12 |
| BP | GO:0007596 | blood coagulation | 6.36362E-07 | 23 |
| BP | GO:0002637 | regulation of immunoglobulin production | 6.41419E-07 | 10 |
| BP | GO:0001913 | T cell mediated cytotoxicity | 6.49504E-07 | 9 |
| BP | GO:0034612 | response to tumor necrosis factor | 6.80206E-07 | 22 |
| BP | GO:0051056 | regulation of small GTPase mediated signal transduction | 7.05541E-07 | 23 |
| BP | GO:0030168 | platelet activation | 7.297E-07 | 15 |
| BP | GO:0072539 | T-helper 17 cell differentiation | 7.41925E-07 | 7 |
| BP | GO:0032102 | negative regulation of response to external stimulus | 7.47198E-07 | 24 |
| BP | GO:0045072 | regulation of interferon-gamma biosynthetic process | 7.90264E-07 | 6 |
| BP | GO:0071356 | cellular response to tumor necrosis factor | 8.40168E-07 | 21 |
| BP | GO:0050817 | coagulation | 8.65048E-07 | 23 |
| BP | GO:0001768 | establishment of T cell polarity | 8.73806E-07 | 5 |
| BP | GO:0045348 | positive regulation of MHC class II biosynthetic process | 8.73806E-07 | 5 |
| BP | GO:0006909 | phagocytosis | 9.07347E-07 | 24 |
| BP | GO:0019724 | B cell mediated immunity | 9.14068E-07 | 18 |
| BP | GO:0032620 | interleukin-17 production | 9.17542E-07 | 8 |
| BP | GO:0002712 | regulation of B cell mediated immunity | 9.2718E-07 | 9 |
| BP | GO:0002889 | regulation of immunoglobulin mediated immune response | 9.2718E-07 | 9 |
| BP | GO:0007259 | JAK-STAT cascade | 9.35912E-07 | 15 |
| BP | GO:0007249 | I-kappaB kinase/NF-kappaB signaling | 9.65487E-07 | 20 |
| BP | GO:0002825 | regulation of T-helper 1 type immune response | 9.83792E-07 | 7 |
| BP | GO:0001911 | negative regulation of leukocyte mediated cytotoxicity | 1.16468E-06 | 6 |
| BP | GO:0042095 | interferon-gamma biosynthetic process | 1.16468E-06 | 6 |
| BP | GO:0070233 | negative regulation of T cell apoptotic process | 1.16468E-06 | 6 |
| BP | GO:0001773 | myeloid dendritic cell activation | 1.28841E-06 | 7 |
| BP | GO:0050704 | regulation of interleukin-1 secretion | 1.30198E-06 | 9 |
| BP | GO:1905517 | macrophage migration | 1.30198E-06 | 9 |
| BP | GO:0097553 | calcium ion transmembrane import into cytosol | 1.34749E-06 | 14 |
| BP | GO:0002369 | T cell cytokine production | 1.39145E-06 | 8 |
| BP | GO:1902106 | negative regulation of leukocyte differentiation | 1.52743E-06 | 12 |
| BP | GO:0001767 | establishment of lymphocyte polarity | 1.57465E-06 | 5 |
| BP | GO:0051924 | regulation of calcium ion transport | 1.64883E-06 | 19 |
| BP | GO:0002313 | mature B cell differentiation involved in immune response | 1.67247E-06 | 6 |
| BP | GO:0002544 | chronic inflammatory response | 1.67247E-06 | 6 |
| BP | GO:0002710 | negative regulation of T cell mediated immunity | 1.67247E-06 | 6 |
| BP | GO:0006959 | humoral immune response | 1.72022E-06 | 23 |
| BP | GO:0034121 | regulation of toll-like receptor signaling pathway | 1.74959E-06 | 10 |
| BP | GO:0050766 | positive regulation of phagocytosis | 1.74959E-06 | 10 |
| BP | GO:0042119 | neutrophil activation | 2.04134E-06 | 28 |
| BP | GO:0032088 | negative regulation of NF-kappaB transcription factor activity | 2.0701E-06 | 11 |
| BP | GO:0002262 | myeloid cell homeostasis | 2.4181E-06 | 14 |
| BP | GO:0050728 | negative regulation of inflammatory response | 2.57254E-06 | 15 |
| BP | GO:0007254 | JNK cascade | 2.58449E-06 | 17 |
| BP | GO:0071674 | mononuclear cell migration | 2.59291E-06 | 11 |
| BP | GO:0014065 | phosphatidylinositol 3-kinase signaling | 2.6211E-06 | 14 |
| BP | GO:0051048 | negative regulation of secretion | 2.6505E-06 | 18 |
| BP | GO:0002468 | dendritic cell antigen processing and presentation | 2.65336E-06 | 5 |
| BP | GO:0045579 | positive regulation of B cell differentiation | 2.65336E-06 | 5 |
| BP | GO:0072540 | T-helper 17 cell lineage commitment | 2.65336E-06 | 5 |
| BP | GO:0002724 | regulation of T cell cytokine production | 2.71157E-06 | 7 |
| BP | GO:0051482 | positive regulation of cytosolic calcium ion concentration  involved in phospholipase C-activating G  protein-coupled signaling pathway | 2.71157E-06 | 7 |
| BP | GO:0002793 | positive regulation of peptide secretion | 2.76973E-06 | 20 |
| BP | GO:0051224 | negative regulation of protein transport | 2.93182E-06 | 16 |
| BP | GO:0001779 | natural killer cell differentiation | 3.22914E-06 | 6 |
| BP | GO:0016064 | immunoglobulin mediated immune response | 3.32389E-06 | 17 |
| BP | GO:0050714 | positive regulation of protein secretion | 3.63794E-06 | 19 |
| BP | GO:0051896 | regulation of protein kinase B signaling | 3.76634E-06 | 18 |
| BP | GO:1904950 | negative regulation of establishment of protein localization | 3.82594E-06 | 16 |
| BP | GO:0043491 | protein kinase B signaling | 3.84104E-06 | 19 |
| BP | GO:0045807 | positive regulation of endocytosis | 3.88198E-06 | 14 |
| BP | GO:0032480 | negative regulation of type I interferon production | 4.2342E-06 | 8 |
| BP | GO:0035723 | interleukin-15-mediated signaling pathway | 4.23822E-06 | 5 |
| BP | GO:0071350 | cellular response to interleukin-15 | 4.23822E-06 | 5 |
| BP | GO:0050718 | positive regulation of interleukin-1 beta secretion | 4.25024E-06 | 7 |
| BP | GO:0051897 | positive regulation of protein kinase B signaling | 4.25559E-06 | 15 |
| BP | GO:0031342 | negative regulation of cell killing | 4.36256E-06 | 6 |
| BP | GO:0051090 | regulation of DNA-binding transcription factor activity | 4.40068E-06 | 25 |
| BP | GO:0045637 | regulation of myeloid cell differentiation | 5.59177E-06 | 18 |
| BP | GO:0032703 | negative regulation of interleukin-2 production | 5.79929E-06 | 6 |
| BP | GO:0048015 | phosphatidylinositol-mediated signaling | 6.00067E-06 | 15 |
| BP | GO:0050848 | regulation of calcium-mediated signaling | 6.02029E-06 | 11 |
| BP | GO:0032689 | negative regulation of interferon-gamma production | 6.45489E-06 | 7 |
| BP | GO:0032735 | positive regulation of interleukin-12 production | 6.45489E-06 | 7 |
| BP | GO:0045346 | regulation of MHC class II biosynthetic process | 6.48046E-06 | 5 |
| BP | GO:0046629 | gamma-delta T cell activation | 6.48046E-06 | 5 |
| BP | GO:0050862 | positive regulation of T cell receptor signaling pathway | 6.48046E-06 | 5 |
| BP | GO:0070672 | response to interleukin-15 | 6.48046E-06 | 5 |
| BP | GO:0002831 | regulation of response to biotic stimulus | 6.75889E-06 | 13 |
| BP | GO:0035023 | regulation of Rho protein signal transduction | 7.31281E-06 | 13 |
| BP | GO:0046330 | positive regulation of JNK cascade | 7.31281E-06 | 13 |
| BP | GO:0048017 | inositol lipid-mediated signaling | 7.33064E-06 | 15 |
| BP | GO:0050730 | regulation of peptidyl-tyrosine phosphorylation | 7.34741E-06 | 18 |
| BP | GO:0002861 | regulation of inflammatory response to antigenic stimulus | 7.59752E-06 | 6 |
| BP | GO:0034162 | toll-like receptor 9 signaling pathway | 7.59752E-06 | 6 |
| BP | GO:0046328 | regulation of JNK cascade | 7.82896E-06 | 15 |
| BP | GO:0002755 | MyD88-dependent toll-like receptor signaling pathway | 7.87085E-06 | 7 |
| BP | GO:0007265 | Ras protein signal transduction | 8.27199E-06 | 25 |
| BP | GO:0043029 | T cell homeostasis | 9.53527E-06 | 7 |
| BP | GO:0045730 | respiratory burst | 9.53527E-06 | 7 |
| BP | GO:0002467 | germinal center formation | 9.55516E-06 | 5 |
| BP | GO:0045342 | MHC class II biosynthetic process | 9.55516E-06 | 5 |
| BP | GO:0002719 | negative regulation of cytokine production  involved in immune response | 9.82242E-06 | 6 |
| BP | GO:0043122 | regulation of I-kappaB kinase/NF-kappaB signaling | 1.01333E-05 | 17 |
| BP | GO:0031098 | stress-activated protein kinase signaling cascade | 1.05781E-05 | 20 |
| BP | GO:0046578 | regulation of Ras protein signal transduction | 1.07089E-05 | 17 |
| BP | GO:0033627 | cell adhesion mediated by integrin | 1.10025E-05 | 9 |
| BP | GO:0050716 | positive regulation of interleukin-1 secretion | 1.14812E-05 | 7 |
| BP | GO:1904892 | regulation of STAT cascade | 1.15583E-05 | 13 |
| BP | GO:0050731 | positive regulation of peptidyl-tyrosine phosphorylation | 1.22465E-05 | 15 |
| BP | GO:0014068 | positive regulation of phosphatidylinositol 3-kinase signaling | 1.28986E-05 | 10 |
| BP | GO:0002643 | regulation of tolerance induction | 1.36618E-05 | 5 |
| BP | GO:1905521 | regulation of macrophage migration | 1.37448E-05 | 7 |
| BP | GO:1901623 | regulation of lymphocyte chemotaxis | 1.58502E-05 | 6 |
| BP | GO:0010819 | regulation of T cell chemotaxis | 1.9025E-05 | 5 |
| BP | GO:0050688 | regulation of defense response to virus | 1.97828E-05 | 9 |
| BP | GO:1904894 | positive regulation of STAT cascade | 2.12042E-05 | 10 |
| BP | GO:0060760 | positive regulation of response to cytokine stimulus | 2.19712E-05 | 8 |
| BP | GO:1903707 | negative regulation of hemopoiesis | 2.19746E-05 | 13 |
| BP | GO:0032677 | regulation of interleukin-8 production | 2.21144E-05 | 9 |
| BP | GO:0007266 | Rho protein signal transduction | 2.37015E-05 | 15 |
| BP | GO:0035025 | positive regulation of Rho protein signal transduction | 2.45573E-05 | 6 |
| BP | GO:0070229 | negative regulation of lymphocyte apoptotic process | 2.45573E-05 | 6 |
| BP | GO:0002200 | somatic diversification of immune receptors | 2.46754E-05 | 9 |
| BP | GO:0032693 | negative regulation of interleukin-10 production | 2.58944E-05 | 5 |
| BP | GO:0002702 | positive regulation of production of molecular  mediator of immune response | 2.81203E-05 | 10 |
| BP | GO:0019884 | antigen processing and presentation of exogenous antigen | 2.83104E-05 | 14 |
| BP | GO:0010959 | regulation of metal ion transport | 2.86121E-05 | 22 |
| BP | GO:0046425 | regulation of JAK-STAT cascade | 2.9488E-05 | 12 |
| BP | GO:0045954 | positive regulation of natural killer cell mediated cytotoxicity | 3.01623E-05 | 6 |
| BP | GO:0071622 | regulation of granulocyte chemotaxis | 3.13179E-05 | 7 |
| BP | GO:0051403 | stress-activated MAPK cascade | 3.27231E-05 | 18 |
| BP | GO:1901222 | regulation of NIK/NF-kappaB signaling | 3.27957E-05 | 11 |
| BP | GO:0045063 | T-helper 1 cell differentiation | 3.4545E-05 | 5 |
| BP | GO:0032621 | interleukin-18 production | 3.49303E-05 | 4 |
| BP | GO:0072610 | interleukin-12 secretion | 3.49303E-05 | 4 |
| BP | GO:0009612 | response to mechanical stimulus | 3.51914E-05 | 15 |
| BP | GO:0034113 | heterotypic cell-cell adhesion | 3.64242E-05 | 8 |
| BP | GO:0045581 | negative regulation of T cell differentiation | 3.64309E-05 | 7 |
| BP | GO:0050764 | regulation of phagocytosis | 3.68842E-05 | 10 |
| BP | GO:0032872 | regulation of stress-activated MAPK cascade | 3.86548E-05 | 16 |
| BP | GO:0002562 | somatic diversification of immune receptors via  germline recombination within a single locus | 4.10716E-05 | 8 |
| BP | GO:0016444 | somatic cell DNA recombination | 4.10716E-05 | 8 |
| BP | GO:0033628 | regulation of cell adhesion mediated by integrin | 4.22098E-05 | 7 |
| BP | GO:0071675 | regulation of mononuclear cell migration | 4.22098E-05 | 7 |
| BP | GO:0070302 | regulation of stress-activated protein kinase signaling cascade | 4.27751E-05 | 16 |
| BP | GO:0007200 | phospholipase C-activating G protein-coupled  receptor signaling pathway | 4.39425E-05 | 10 |
| BP | GO:0032814 | regulation of natural killer cell activation | 4.44446E-05 | 6 |
| BP | GO:0002827 | positive regulation of T-helper 1 type immune response | 4.52775E-05 | 5 |
| BP | GO:0033630 | positive regulation of cell adhesion mediated by integrin | 4.52775E-05 | 5 |
| BP | GO:0050860 | negative regulation of T cell receptor signaling pathway | 4.52775E-05 | 5 |
| BP | GO:0038093 | Fc receptor signaling pathway | 4.72783E-05 | 16 |
| BP | GO:0032637 | interleukin-8 production | 5.06732E-05 | 9 |
| BP | GO:0046579 | positive regulation of Ras protein signal transduction | 5.18589E-05 | 8 |
| BP | GO:0002717 | positive regulation of natural killer cell mediated immunity | 5.33767E-05 | 6 |
| BP | GO:0002517 | T cell tolerance induction | 5.39906E-05 | 4 |
| BP | GO:0002604 | regulation of dendritic cell antigen processing and presentation | 5.39906E-05 | 4 |
| BP | GO:0002638 | negative regulation of immunoglobulin production | 5.39906E-05 | 4 |
| BP | GO:0002923 | regulation of humoral immune response mediated  by circulating immunoglobulin | 5.39906E-05 | 4 |
| BP | GO:0033089 | positive regulation of T cell differentiation in thymus | 5.39906E-05 | 4 |
| BP | GO:0035747 | natural killer cell chemotaxis | 5.39906E-05 | 4 |
| BP | GO:0045351 | type I interferon biosynthetic process | 5.39906E-05 | 4 |
| BP | GO:0070757 | interleukin-35-mediated signaling pathway | 5.39906E-05 | 4 |
| BP | GO:0001774 | microglial cell activation | 5.60293E-05 | 7 |
| BP | GO:0002269 | leukocyte activation involved in inflammatory response | 5.60293E-05 | 7 |
| BP | GO:0043433 | negative regulation of DNA-binding transcription factor activity | 5.78498E-05 | 13 |
| BP | GO:0002548 | monocyte chemotaxis | 5.80792E-05 | 8 |
| BP | GO:0140131 | positive regulation of lymphocyte chemotaxis | 5.84177E-05 | 5 |
| BP | GO:1901739 | regulation of myoblast fusion | 5.84177E-05 | 5 |
| BP | GO:2000316 | regulation of T-helper 17 type immune response | 5.84177E-05 | 5 |
| BP | GO:0002686 | negative regulation of leukocyte migration | 6.42124E-05 | 7 |
| BP | GO:0032874 | positive regulation of stress-activated MAPK cascade | 6.52512E-05 | 13 |
| BP | GO:0070304 | positive regulation of stress-activated protein  kinase signaling cascade | 6.92506E-05 | 13 |
| BP | GO:0001961 | positive regulation of cytokine-mediated signaling pathway | 7.33465E-05 | 7 |
| BP | GO:0045624 | positive regulation of T-helper cell differentiation | 7.43162E-05 | 5 |
| BP | GO:0048305 | immunoglobulin secretion | 7.43162E-05 | 5 |
| BP | GO:0002478 | antigen processing and presentation of exogenous peptide antigen | 7.78916E-05 | 13 |
| BP | GO:0002863 | positive regulation of inflammatory response to antigenic stimulus | 7.96593E-05 | 4 |
| BP | GO:0038110 | interleukin-2-mediated signaling pathway | 7.96593E-05 | 4 |
| BP | GO:0048302 | regulation of isotype switching to IgG isotypes | 7.96593E-05 | 4 |
| BP | GO:0032733 | positive regulation of interleukin-10 production | 8.90611E-05 | 6 |
| BP | GO:1902622 | regulation of neutrophil migration | 8.90611E-05 | 6 |
| BP | GO:0009595 | detection of biotic stimulus | 9.33487E-05 | 5 |
| BP | GO:0046427 | positive regulation of JAK-STAT cascade | 9.67503E-05 | 9 |
| BP | GO:0050691 | regulation of defense response to virus by host | 0.000104453 | 6 |
| BP | GO:0051057 | positive regulation of small GTPase mediated signal transduction | 0.000109863 | 8 |
| BP | GO:0002430 | complement receptor mediated signaling pathway | 0.00011318 | 4 |
| BP | GO:0032823 | regulation of natural killer cell differentiation | 0.00011318 | 4 |
| BP | GO:0033623 | regulation of integrin activation | 0.00011318 | 4 |
| BP | GO:0045078 | positive regulation of interferon-gamma biosynthetic process | 0.00011318 | 4 |
| BP | GO:0048291 | isotype switching to IgG isotypes | 0.00011318 | 4 |
| BP | GO:0071352 | cellular response to interleukin-2 | 0.00011318 | 4 |
| BP | GO:0072672 | neutrophil extravasation | 0.00011318 | 4 |
| BP | GO:1902713 | regulation of interferon-gamma secretion | 0.00011318 | 4 |
| BP | GO:0090023 | positive regulation of neutrophil chemotaxis | 0.000115915 | 5 |
| BP | GO:0010524 | positive regulation of calcium ion transport into cytosol | 0.000121104 | 7 |
| BP | GO:0043900 | regulation of multi-organism process | 0.000123938 | 21 |
| BP | GO:0030218 | erythrocyte differentiation | 0.000133626 | 10 |
| BP | GO:0009988 | cell-cell recognition | 0.000133893 | 8 |
| BP | GO:0043030 | regulation of macrophage activation | 0.000136318 | 7 |
| BP | GO:0043551 | regulation of phosphatidylinositol 3-kinase activity | 0.000136318 | 7 |
| BP | GO:0045620 | negative regulation of lymphocyte differentiation | 0.000136318 | 7 |
| BP | GO:0050901 | leukocyte tethering or rolling | 0.000142438 | 5 |
| BP | GO:0090025 | regulation of monocyte chemotaxis | 0.000142438 | 5 |
| BP | GO:0044546 | NLRP3 inflammasome complex assembly | 0.000155859 | 4 |
| BP | GO:0045591 | positive regulation of regulatory T cell differentiation | 0.000155859 | 4 |
| BP | GO:0070669 | response to interleukin-2 | 0.000155859 | 4 |
| BP | GO:0150076 | neuroinflammatory response | 0.0001621 | 8 |
| BP | GO:0048002 | antigen processing and presentation of peptide antigen | 0.000168993 | 13 |
| BP | GO:0070374 | positive regulation of ERK1 and ERK2 cascade | 0.000170676 | 14 |
| BP | GO:0001916 | positive regulation of T cell mediated cytotoxicity | 0.000173364 | 5 |
| BP | GO:0010758 | regulation of macrophage chemotaxis | 0.000173364 | 5 |
| BP | GO:0071624 | positive regulation of granulocyte chemotaxis | 0.000173364 | 5 |
| BP | GO:0050798 | activated T cell proliferation | 0.000188368 | 6 |
| BP | GO:0031663 | lipopolysaccharide-mediated signaling pathway | 0.000191484 | 7 |
| BP | GO:0061900 | glial cell activation | 0.000191484 | 7 |
| BP | GO:0032481 | positive regulation of type I interferon production | 0.000195025 | 8 |
| BP | GO:0051235 | maintenance of location | 0.000202067 | 18 |
| BP | GO:0019886 | antigen processing and presentation of exogenous  peptide antigen via MHC class II | 0.00020336 | 9 |
| BP | GO:0002716 | negative regulation of natural killer cell mediated immunity | 0.000209061 | 4 |
| BP | GO:0051770 | positive regulation of nitric-oxide synthase biosynthetic process | 0.000209061 | 4 |
| BP | GO:2001185 | regulation of CD8-positive, alpha-beta T cell activation | 0.000209061 | 4 |
| BP | GO:0097028 | dendritic cell differentiation | 0.000215954 | 6 |
| BP | GO:0043312 | neutrophil degranulation | 0.000222353 | 23 |
| BP | GO:0071260 | cellular response to mechanical stimulus | 0.000233251 | 8 |
| BP | GO:0034101 | erythrocyte homeostasis | 0.000233714 | 10 |
| BP | GO:0045576 | mast cell activation | 0.000237365 | 7 |
| BP | GO:0002283 | neutrophil activation involved in immune response | 0.000242883 | 23 |
| BP | GO:0050850 | positive regulation of calcium-mediated signaling | 0.000246626 | 6 |
| BP | GO:0051928 | positive regulation of calcium ion transport | 0.000249782 | 10 |
| BP | GO:2000108 | positive regulation of leukocyte apoptotic process | 0.000250324 | 5 |
| BP | GO:0002495 | antigen processing and presentation of peptide  antigen via MHC class II | 0.000255552 | 9 |
| BP | GO:0001780 | neutrophil homeostasis | 0.000274194 | 4 |
| BP | GO:0010820 | positive regulation of T cell chemotaxis | 0.000274194 | 4 |
| BP | GO:0033631 | cell-cell adhesion mediated by integrin | 0.000274194 | 4 |
| BP | GO:0002504 | antigen processing and presentation of peptide or  polysaccharide antigen via MHC class II | 0.000275239 | 9 |
| BP | GO:1903532 | positive regulation of secretion by cell | 0.000278729 | 20 |
| BP | GO:0030100 | regulation of endocytosis | 0.000280319 | 16 |
| BP | GO:0061756 | leukocyte adhesion to vascular endothelial cell | 0.000280622 | 6 |
| BP | GO:0051092 | positive regulation of NF-kappaB transcription factor activity | 0.000288912 | 11 |
| BP | GO:0060142 | regulation of syncytium formation by plasma membrane fusion | 0.000297359 | 5 |
| BP | GO:1902624 | positive regulation of neutrophil migration | 0.000297359 | 5 |
| BP | GO:0002446 | neutrophil mediated immunity | 0.000333202 | 23 |
| BP | GO:0070588 | calcium ion transmembrane transport | 0.000341212 | 17 |
| BP | GO:0090022 | regulation of neutrophil chemotaxis | 0.000350799 | 5 |
| BP | GO:0032740 | positive regulation of interleukin-17 production | 0.000352708 | 4 |
| BP | GO:1903975 | regulation of glial cell migration | 0.000352708 | 4 |
| BP | GO:0016445 | somatic diversification of immunoglobulins | 0.000355624 | 7 |
| BP | GO:0043550 | regulation of lipid kinase activity | 0.000355624 | 7 |
| BP | GO:0070371 | ERK1 and ERK2 cascade | 0.000367 | 17 |
| BP | GO:0002204 | somatic recombination of immunoglobulin genes  involved in immune response | 0.000405079 | 6 |
| BP | GO:0002208 | somatic diversification of immunoglobulins  involved in immune response | 0.000405079 | 6 |
| BP | GO:0035722 | interleukin-12-mediated signaling pathway | 0.000405079 | 6 |
| BP | GO:0045190 | isotype switching | 0.000405079 | 6 |
| BP | GO:0007260 | tyrosine phosphorylation of STAT protein | 0.0004183 | 8 |
| BP | GO:0046777 | protein autophosphorylation | 0.000423971 | 14 |
| BP | GO:0070230 | positive regulation of lymphocyte apoptotic process | 0.000446083 | 4 |
| BP | GO:0090026 | positive regulation of monocyte chemotaxis | 0.000446083 | 4 |
| BP | GO:2000319 | regulation of T-helper 17 cell differentiation | 0.000446083 | 4 |
| BP | GO:0032648 | regulation of interferon-beta production | 0.000454944 | 6 |
| BP | GO:0038061 | NIK/NF-kappaB signaling | 0.000460813 | 12 |
| BP | GO:0043123 | positive regulation of I-kappaB kinase/NF-kappaB signaling | 0.000460813 | 12 |
| BP | GO:0043552 | positive regulation of phosphatidylinositol 3-kinase activity | 0.000479109 | 5 |
| BP | GO:0051222 | positive regulation of protein transport | 0.000504949 | 20 |
| BP | GO:0002448 | mast cell mediated immunity | 0.000509464 | 6 |
| BP | GO:0032757 | positive regulation of interleukin-8 production | 0.000509464 | 6 |
| BP | GO:0071349 | cellular response to interleukin-12 | 0.000509464 | 6 |
| BP | GO:0045191 | regulation of isotype switching | 0.000555132 | 5 |
| BP | GO:0002726 | positive regulation of T cell cytokine production | 0.000555826 | 4 |
| BP | GO:0002922 | positive regulation of humoral immune response | 0.000555826 | 4 |
| BP | GO:0032700 | negative regulation of interleukin-17 production | 0.000555826 | 4 |
| BP | GO:1901741 | positive regulation of myoblast fusion | 0.000555826 | 4 |
| BP | GO:0032608 | interferon-beta production | 0.000568935 | 6 |
| BP | GO:0070671 | response to interleukin-12 | 0.000568935 | 6 |
| BP | GO:0070372 | regulation of ERK1 and ERK2 cascade | 0.000575296 | 16 |
| BP | GO:1904951 | positive regulation of establishment of protein localization | 0.000605773 | 21 |
| BP | GO:0045639 | positive regulation of myeloid cell differentiation | 0.000612479 | 8 |
| BP | GO:0010922 | positive regulation of phosphatase activity | 0.000639864 | 5 |
| BP | GO:0051047 | positive regulation of secretion | 0.000678352 | 20 |
| BP | GO:0035458 | cellular response to interferon-beta | 0.000683463 | 4 |
| BP | GO:0043371 | negative regulation of CD4-positive, alpha-beta  T cell differentiation | 0.000683463 | 4 |
| BP | GO:0051767 | nitric-oxide synthase biosynthetic process | 0.000683463 | 4 |
| BP | GO:0051769 | regulation of nitric-oxide synthase biosynthetic process | 0.000683463 | 4 |
| BP | GO:1905523 | positive regulation of macrophage migration | 0.000683463 | 4 |
| BP | GO:0016447 | somatic recombination of immunoglobulin gene segments | 0.000703943 | 6 |
| BP | GO:0002532 | production of molecular mediator involved in  inflammatory response | 0.000731813 | 7 |
| BP | GO:0043405 | regulation of MAP kinase activity | 0.00073251 | 17 |
| BP | GO:0042092 | type 2 immune response | 0.00073392 | 5 |
| BP | GO:0070423 | nucleotide-binding oligomerization domain  containing signaling pathway | 0.00073392 | 5 |
| BP | GO:0002761 | regulation of myeloid leukocyte differentiation | 0.000756157 | 9 |
| BP | GO:0060337 | type I interferon signaling pathway | 0.000815363 | 8 |
| BP | GO:0071357 | cellular response to type I interferon | 0.000815363 | 8 |
| BP | GO:0002438 | acute inflammatory response to antigenic stimulus | 0.000830533 | 4 |
| BP | GO:0035743 | CD4-positive, alpha-beta T cell cytokine production | 0.000830533 | 4 |
| BP | GO:0035872 | nucleotide-binding domain, leucine rich repeat containing  receptor signaling pathway | 0.000837929 | 5 |
| BP | GO:0090218 | positive regulation of lipid kinase activity | 0.000837929 | 5 |
| BP | GO:0097191 | extrinsic apoptotic signaling pathway | 0.000859251 | 13 |
| BP | GO:0002720 | positive regulation of cytokine production involved in  immune response | 0.000862487 | 6 |
| BP | GO:0001960 | negative regulation of cytokine-mediated signaling pathway | 0.00093505 | 7 |
| BP | GO:0030316 | osteoclast differentiation | 0.00093532 | 8 |
| BP | GO:0002714 | positive regulation of B cell mediated immunity | 0.000952535 | 5 |
| BP | GO:0002891 | positive regulation of immunoglobulin mediated immune response | 0.000952535 | 5 |
| BP | GO:0038083 | peptidyl-tyrosine autophosphorylation | 0.000952535 | 5 |
| BP | GO:0046006 | regulation of activated T cell proliferation | 0.000952535 | 5 |
| BP | GO:0002713 | negative regulation of B cell mediated immunity | 0.000958739 | 3 |
| BP | GO:0002890 | negative regulation of immunoglobulin mediated immune response | 0.000958739 | 3 |
| BP | GO:0044803 | multi-organism membrane organization | 0.000958739 | 3 |
| BP | GO:0045625 | regulation of T-helper 1 cell differentiation | 0.000958739 | 3 |
| BP | GO:0048304 | positive regulation of isotype switching to IgG isotypes | 0.000958739 | 3 |
| BP | GO:0050861 | positive regulation of B cell receptor signaling pathway | 0.000958739 | 3 |
| BP | GO:0071104 | response to interleukin-9 | 0.000958739 | 3 |
| BP | GO:0097048 | dendritic cell apoptotic process | 0.000958739 | 3 |
| BP | GO:2000668 | regulation of dendritic cell apoptotic process | 0.000958739 | 3 |
| BP | GO:0046718 | viral entry into host cell | 0.000962092 | 9 |
| BP | GO:0006925 | inflammatory cell apoptotic process | 0.000998581 | 4 |
| BP | GO:0032727 | positive regulation of interferon-alpha production | 0.000998581 | 4 |
| BP | GO:0033622 | integrin activation | 0.000998581 | 4 |
| BP | GO:0034340 | response to type I interferon | 0.001069018 | 8 |
| BP | GO:0046636 | negative regulation of alpha-beta T cell activation | 0.001078391 | 5 |
| BP | GO:0048246 | macrophage chemotaxis | 0.001078391 | 5 |
| BP | GO:0051091 | positive regulation of DNA-binding transcription factor activity | 0.001182189 | 14 |
| BP | GO:0034695 | response to prostaglandin E | 0.001189157 | 4 |
| BP | GO:0050830 | defense response to Gram-positive bacterium | 0.001217562 | 8 |
| BP | GO:0032722 | positive regulation of chemokine production | 0.001260872 | 6 |
| BP | GO:0035306 | positive regulation of dephosphorylation | 0.001260872 | 6 |
| BP | GO:0010522 | regulation of calcium ion transport into cytosol | 0.00129776 | 8 |
| BP | GO:0002887 | negative regulation of myeloid leukocyte mediated immunity | 0.00129802 | 3 |
| BP | GO:0033632 | regulation of cell-cell adhesion mediated by integrin | 0.00129802 | 3 |
| BP | GO:0046007 | negative regulation of activated T cell proliferation | 0.00129802 | 3 |
| BP | GO:0070106 | interleukin-27-mediated signaling pathway | 0.00129802 | 3 |
| BP | GO:2000318 | positive regulation of T-helper 17 type immune response | 0.00129802 | 3 |
| BP | GO:0019835 | cytolysis | 0.001366522 | 5 |
| BP | GO:0042036 | negative regulation of cytokine biosynthetic process | 0.001366522 | 5 |
| BP | GO:0098760 | response to interleukin-7 | 0.001366522 | 5 |
| BP | GO:0098761 | cellular response to interleukin-7 | 0.001366522 | 5 |
| BP | GO:0046847 | filopodium assembly | 0.00137945 | 6 |
| BP | GO:0070527 | platelet aggregation | 0.00137945 | 6 |
| BP | GO:0033081 | regulation of T cell differentiation in thymus | 0.001403805 | 4 |
| BP | GO:0042832 | defense response to protozoan | 0.001403805 | 4 |
| BP | GO:0046639 | negative regulation of alpha-beta T cell differentiation | 0.001403805 | 4 |
| BP | GO:0071677 | positive regulation of mononuclear cell migration | 0.001403805 | 4 |
| BP | GO:0034109 | homotypic cell-cell adhesion | 0.001473342 | 7 |
| BP | GO:0060761 | negative regulation of response to cytokine stimulus | 0.001473342 | 7 |
| BP | GO:0007520 | myoblast fusion | 0.001530155 | 5 |
| BP | GO:0042742 | defense response to bacterium | 0.001566397 | 16 |
| BP | GO:0032642 | regulation of chemokine production | 0.001582674 | 7 |
| BP | GO:0010803 | regulation of tumor necrosis factor-mediated signaling pathway | 0.001641941 | 6 |
| BP | GO:0001562 | response to protozoan | 0.001644065 | 4 |
| BP | GO:0060330 | regulation of response to interferon-gamma | 0.001644065 | 4 |
| BP | GO:0060334 | regulation of interferon-gamma-mediated signaling pathway | 0.001644065 | 4 |
| BP | GO:2000482 | regulation of interleukin-8 secretion | 0.001644065 | 4 |
| BP | GO:0042509 | regulation of tyrosine phosphorylation of STAT protein | 0.001698221 | 7 |
| BP | GO:0045416 | positive regulation of interleukin-8 biosynthetic process | 0.00170414 | 3 |
| BP | GO:0070486 | leukocyte aggregation | 0.00170414 | 3 |
| BP | GO:0097340 | inhibition of cysteine-type endopeptidase activity | 0.00170414 | 3 |
| BP | GO:0097341 | zymogen inhibition | 0.00170414 | 3 |
| BP | GO:1900165 | negative regulation of interleukin-6 secretion | 0.00170414 | 3 |
| BP | GO:1905522 | negative regulation of macrophage migration | 0.00170414 | 3 |
| BP | GO:0010559 | regulation of glycoprotein biosynthetic process | 0.001707749 | 5 |
| BP | GO:0071902 | positive regulation of protein serine/threonine kinase activity | 0.001770684 | 16 |
| BP | GO:0008037 | cell recognition | 0.001868045 | 12 |
| BP | GO:0030866 | cortical actin cytoskeleton organization | 0.001900002 | 5 |
| BP | GO:0002230 | positive regulation of defense response to virus by host | 0.001911465 | 4 |
| BP | GO:0043270 | positive regulation of ion transport | 0.001929703 | 14 |
| BP | GO:0030260 | entry into host cell | 0.001964533 | 9 |
| BP | GO:0044409 | entry into host | 0.001964533 | 9 |
| BP | GO:0051806 | entry into cell of other organism involved in symbiotic interaction | 0.001964533 | 9 |
| BP | GO:0051828 | entry into other organism involved in symbiotic interaction | 0.001964533 | 9 |
| BP | GO:0006919 | activation of cysteine-type endopeptidase activity  involved in apoptotic process | 0.002084529 | 7 |
| BP | GO:0045670 | regulation of osteoclast differentiation | 0.002104713 | 6 |
| BP | GO:0070266 | necroptotic process | 0.002107615 | 5 |
| BP | GO:0002679 | respiratory burst involved in defense response | 0.002181428 | 3 |
| BP | GO:0002864 | regulation of acute inflammatory response to antigenic stimulus | 0.002181428 | 3 |
| BP | GO:0043301 | negative regulation of leukocyte degranulation | 0.002181428 | 3 |
| BP | GO:0045779 | negative regulation of bone resorption | 0.002181428 | 3 |
| BP | GO:0050713 | negative regulation of interleukin-1 beta secretion | 0.002181428 | 3 |
| BP | GO:0070431 | nucleotide-binding oligomerization domain  containing 2 signaling pathway | 0.002181428 | 3 |
| BP | GO:1900225 | regulation of NLRP3 inflammasome complex assembly | 0.002181428 | 3 |
| BP | GO:0043032 | positive regulation of macrophage activation | 0.002207518 | 4 |
| BP | GO:0045671 | negative regulation of osteoclast differentiation | 0.002207518 | 4 |
| BP | GO:0060143 | positive regulation of syncytium formation by  plasma membrane fusion | 0.002207518 | 4 |
| BP | GO:1904019 | epithelial cell apoptotic process | 0.002224828 | 8 |
| BP | GO:1903725 | regulation of phospholipid metabolic process | 0.002377575 | 7 |
| BP | GO:0002691 | regulation of cellular extravasation | 0.002533717 | 4 |
| BP | GO:0035456 | response to interferon-beta | 0.002533717 | 4 |
| BP | GO:0032602 | chemokine production | 0.002535501 | 7 |
| BP | GO:0045953 | negative regulation of natural killer cell mediated cytotoxicity | 0.002733855 | 3 |
| BP | GO:0098543 | detection of other organism | 0.002733855 | 3 |
| BP | GO:0002832 | negative regulation of response to biotic stimulus | 0.002829682 | 5 |
| BP | GO:0043303 | mast cell degranulation | 0.002829682 | 5 |
| BP | GO:0045646 | regulation of erythrocyte differentiation | 0.002829682 | 5 |
| BP | GO:0072606 | interleukin-8 secretion | 0.002891534 | 4 |
| BP | GO:2000515 | negative regulation of CD4-positive, alpha-beta T cell activation | 0.002891534 | 4 |
| BP | GO:0043406 | positive regulation of MAP kinase activity | 0.003017383 | 13 |
| BP | GO:0106106 | cold-induced thermogenesis | 0.003052068 | 9 |
| BP | GO:0120161 | regulation of cold-induced thermogenesis | 0.003052068 | 9 |
| BP | GO:0002279 | mast cell activation involved in immune response | 0.003105815 | 5 |
| BP | GO:0030865 | cortical cytoskeleton organization | 0.003105815 | 5 |
| BP | GO:1903727 | positive regulation of phospholipid metabolic process | 0.003105815 | 5 |
| BP | GO:0043901 | negative regulation of multi-organism process | 0.003128236 | 10 |
| BP | GO:0002828 | regulation of type 2 immune response | 0.003282417 | 4 |
| BP | GO:0032728 | positive regulation of interferon-beta production | 0.003282417 | 4 |
| BP | GO:0038111 | interleukin-7-mediated signaling pathway | 0.003282417 | 4 |
| BP | GO:0048873 | homeostasis of number of cells within a tissue | 0.003282417 | 4 |
| BP | GO:0010759 | positive regulation of macrophage chemotaxis | 0.003365054 | 3 |
| BP | GO:0030852 | regulation of granulocyte differentiation | 0.003365054 | 3 |
| BP | GO:0032645 | regulation of granulocyte macrophage  colony-stimulating factor production | 0.003365054 | 3 |
| BP | GO:0034134 | toll-like receptor 2 signaling pathway | 0.003365054 | 3 |
| BP | GO:0046851 | negative regulation of bone remodeling | 0.003365054 | 3 |
| BP | GO:2000402 | negative regulation of lymphocyte migration | 0.003365054 | 3 |
| BP | GO:2000484 | positive regulation of interleukin-8 secretion | 0.003365054 | 3 |
| BP | GO:0002534 | cytokine production involved in inflammatory response | 0.003400854 | 5 |
| BP | GO:0043124 | negative regulation of I-kappaB kinase/NF-kappaB signaling | 0.003400854 | 5 |
| BP | GO:0097300 | programmed necrotic cell death | 0.003400854 | 5 |
| BP | GO:1903018 | regulation of glycoprotein metabolic process | 0.003400854 | 5 |
| BP | GO:0046488 | phosphatidylinositol metabolic process | 0.003542047 | 10 |
| BP | GO:0042531 | positive regulation of tyrosine phosphorylation of STAT protein | 0.003561977 | 6 |
| BP | GO:0120032 | regulation of plasma membrane bounded cell projection assembly | 0.003689245 | 10 |
| BP | GO:0010543 | regulation of platelet activation | 0.003707783 | 4 |
| BP | GO:0033198 | response to ATP | 0.003707783 | 4 |
| BP | GO:0034694 | response to prostaglandin | 0.003707783 | 4 |
| BP | GO:0045648 | positive regulation of erythrocyte differentiation | 0.003707783 | 4 |
| BP | GO:0060491 | regulation of cell projection assembly | 0.003998178 | 10 |
| BP | GO:0032604 | granulocyte macrophage colony-stimulating factor production | 0.004078329 | 3 |
| BP | GO:0043031 | negative regulation of macrophage activation | 0.004078329 | 3 |
| BP | GO:0045623 | negative regulation of T-helper cell differentiation | 0.004078329 | 3 |
| BP | GO:0050711 | negative regulation of interleukin-1 secretion | 0.004078329 | 3 |
| BP | GO:0031334 | positive regulation of protein complex assembly | 0.004162322 | 13 |
| BP | GO:0071353 | cellular response to interleukin-4 | 0.004169022 | 4 |
| BP | GO:0008360 | regulation of cell shape | 0.00418838 | 9 |
| BP | GO:0031529 | ruffle organization | 0.004406512 | 5 |
| BP | GO:2000116 | regulation of cysteine-type endopeptidase activity | 0.004419428 | 12 |
| BP | GO:0045165 | cell fate commitment | 0.004428758 | 13 |
| BP | GO:0042326 | negative regulation of phosphorylation | 0.004435368 | 19 |
| BP | GO:0007163 | establishment or maintenance of cell polarity | 0.004606507 | 11 |
| BP | GO:0006471 | protein ADP-ribosylation | 0.00466749 | 4 |
| BP | GO:1901223 | negative regulation of NIK/NF-kappaB signaling | 0.00466749 | 4 |
| BP | GO:0035690 | cellular response to drug | 0.004705329 | 16 |
| BP | GO:0002418 | immune response to tumor cell | 0.004876677 | 3 |
| BP | GO:0002693 | positive regulation of cellular extravasation | 0.004876677 | 3 |
| BP | GO:0048535 | lymph node development | 0.004876677 | 3 |
| BP | GO:0051709 | regulation of killing of cells of other organism | 0.004876677 | 3 |
| BP | GO:1904996 | positive regulation of leukocyte adhesion to vascular endothelial cell | 0.004876677 | 3 |
| BP | GO:1990845 | adaptive thermogenesis | 0.004972052 | 9 |
| BP | GO:0000018 | regulation of DNA recombination | 0.005126201 | 7 |
| BP | GO:0031532 | actin cytoskeleton reorganization | 0.005126201 | 7 |
| BP | GO:2001236 | regulation of extrinsic apoptotic signaling pathway | 0.005184684 | 9 |
| BP | GO:0070670 | response to interleukin-4 | 0.005204507 | 4 |
| BP | GO:0043409 | negative regulation of MAPK cascade | 0.005243676 | 10 |
| BP | GO:1901224 | positive regulation of NIK/NF-kappaB signaling | 0.005316922 | 6 |
| BP | GO:0034767 | positive regulation of ion transmembrane transport | 0.005404299 | 9 |
| BP | GO:0043281 | regulation of cysteine-type endopeptidase activity  involved in apoptotic process | 0.005482377 | 11 |
| BP | GO:0007194 | negative regulation of adenylate cyclase activity | 0.005762795 | 3 |
| BP | GO:0150079 | negative regulation of neuroinflammatory response | 0.005762795 | 3 |
| BP | GO:0034142 | toll-like receptor 4 signaling pathway | 0.005781357 | 4 |
| BP | GO:0000768 | syncytium formation by plasma membrane fusion | 0.006054859 | 5 |
| BP | GO:0140253 | cell-cell fusion | 0.006054859 | 5 |
| BP | GO:0007584 | response to nutrient | 0.006273584 | 11 |
| BP | GO:0002689 | negative regulation of leukocyte chemotaxis | 0.006739102 | 3 |
| BP | GO:0034138 | toll-like receptor 3 signaling pathway | 0.006739102 | 3 |
| BP | GO:0045414 | regulation of interleukin-8 biosynthetic process | 0.006739102 | 3 |
| BP | GO:0098581 | detection of external biotic stimulus | 0.006739102 | 3 |
| BP | GO:0002920 | regulation of humoral immune response | 0.006974821 | 8 |
| BP | GO:0006949 | syncytium formation | 0.00702321 | 5 |
| BP | GO:1902041 | regulation of extrinsic apoptotic signaling pathway  via death domain receptors | 0.00702321 | 5 |
| BP | GO:0010831 | positive regulation of myotube differentiation | 0.007763157 | 4 |
| BP | GO:1904037 | positive regulation of epithelial cell apoptotic process | 0.007763157 | 4 |
| BP | GO:0002374 | cytokine secretion involved in immune response | 0.007807744 | 3 |
| BP | GO:0010560 | positive regulation of glycoprotein biosynthetic process | 0.007807744 | 3 |
| BP | GO:0031280 | negative regulation of cyclase activity | 0.007807744 | 3 |
| BP | GO:0034104 | negative regulation of tissue remodeling | 0.007807744 | 3 |
| BP | GO:0042228 | interleukin-8 biosynthetic process | 0.007807744 | 3 |
| BP | GO:1900017 | positive regulation of cytokine production involved  in inflammatory response | 0.007807744 | 3 |
| BP | GO:1903978 | regulation of microglial cell activation | 0.007807744 | 3 |
| BP | GO:0032692 | negative regulation of interleukin-1 production | 0.008511385 | 4 |
| BP | GO:0045911 | positive regulation of DNA recombination | 0.008511385 | 4 |
| BP | GO:0002433 | immune response-regulating cell surface receptor  signaling pathway involved in phagocytosis | 0.00862478 | 8 |
| BP | GO:0038096 | Fc-gamma receptor signaling pathway involved in  phagocytosis | 0.00862478 | 8 |
| BP | GO:0001933 | negative regulation of protein phosphorylation | 0.008691209 | 17 |
| BP | GO:0032516 | positive regulation of phosphoprotein phosphatase activity | 0.008970613 | 3 |
| BP | GO:0019058 | viral life cycle | 0.009025372 | 14 |
| BP | GO:0043254 | regulation of protein complex assembly | 0.00923437 | 18 |
| BP | GO:0002753 | cytoplasmic pattern recognition receptor signaling pathway | 0.009274861 | 5 |
| BP | GO:0010830 | regulation of myotube differentiation | 0.009274861 | 5 |
| BP | GO:0045453 | bone resorption | 0.009274861 | 5 |
| BP | GO:0070265 | necrotic cell death | 0.009274861 | 5 |
| BP | GO:0051281 | positive regulation of release of sequestered calcium ion into cytosol | 0.009305259 | 4 |
| BP | GO:0038094 | Fc-gamma receptor signaling pathway | 0.00974562 | 8 |
| BP | GO:0007015 | actin filament organization | 0.00986996 | 16 |
| BP | GO:0043506 | regulation of JUN kinase activity | 0.01005976 | 6 |
| BP | GO:0010955 | negative regulation of protein processing | 0.01014581 | 4 |
| BP | GO:0034122 | negative regulation of toll-like receptor signaling pathway | 0.01014581 | 4 |
| BP | GO:0150077 | regulation of neuroinflammatory response | 0.01014581 | 4 |
| BP | GO:1903318 | negative regulation of protein maturation | 0.01014581 | 4 |
| BP | GO:0001659 | temperature homeostasis | 0.010354072 | 9 |
| BP | GO:1904064 | positive regulation of cation transmembrane transport | 0.010550792 | 8 |
| BP | GO:0050922 | negative regulation of chemotaxis | 0.010568333 | 5 |
| BP | GO:0006661 | phosphatidylinositol biosynthetic process | 0.010691457 | 7 |
| BP | GO:0002431 | Fc receptor mediated stimulatory signaling pathway | 0.010971374 | 8 |
| BP | GO:0002639 | positive regulation of immunoglobulin production | 0.011034027 | 4 |
| BP | GO:0006509 | membrane protein ectodomain proteolysis | 0.011034027 | 4 |
| BP | GO:0032958 | inositol phosphate biosynthetic process | 0.011034027 | 4 |
| BP | GO:0046849 | bone remodeling | 0.011168133 | 6 |
| BP | GO:0072577 | endothelial cell apoptotic process | 0.011259117 | 5 |
| BP | GO:0002347 | response to tumor cell | 0.011585384 | 3 |
| BP | GO:0051350 | negative regulation of lyase activity | 0.011585384 | 3 |
| BP | GO:0060055 | angiogenesis involved in wound healing | 0.011585384 | 3 |
| BP | GO:0071496 | cellular response to external stimulus | 0.011805971 | 14 |
| BP | GO:0008154 | actin polymerization or depolymerization | 0.012309236 | 10 |
| BP | GO:0051701 | interaction with host | 0.012309236 | 10 |
| BP | GO:2000117 | negative regulation of cysteine-type endopeptidase activity | 0.012360162 | 6 |
| BP | GO:1903034 | regulation of response to wounding | 0.012735489 | 9 |
| BP | GO:0030225 | macrophage differentiation | 0.012957174 | 4 |
| BP | GO:0035307 | positive regulation of protein dephosphorylation | 0.012957174 | 4 |
| BP | GO:0051489 | regulation of filopodium assembly | 0.012957174 | 4 |
| BP | GO:0031579 | membrane raft organization | 0.013039895 | 3 |
| BP | GO:1903020 | positive regulation of glycoprotein metabolic process | 0.013039895 | 3 |
| BP | GO:0010811 | positive regulation of cell-substrate adhesion | 0.013270384 | 7 |
| BP | GO:1903169 | regulation of calcium ion transmembrane transport | 0.01375958 | 8 |
| BP | GO:0031664 | regulation of lipopolysaccharide-mediated signaling pathway | 0.014593872 | 3 |
| BP | GO:0034110 | regulation of homotypic cell-cell adhesion | 0.014593872 | 3 |
| BP | GO:0045830 | positive regulation of isotype switching | 0.014593872 | 3 |
| BP | GO:0072677 | eosinophil migration | 0.014593872 | 3 |
| BP | GO:1903306 | negative regulation of regulated secretory pathway | 0.014593872 | 3 |
| BP | GO:2000353 | positive regulation of endothelial cell apoptotic process | 0.014593872 | 3 |
| BP | GO:0014013 | regulation of gliogenesis | 0.015016647 | 7 |
| BP | GO:1900015 | regulation of cytokine production involved in inflammatory response | 0.015081664 | 4 |
| BP | GO:0061515 | myeloid cell development | 0.015176339 | 5 |
| BP | GO:0061025 | membrane fusion | 0.015332854 | 8 |
| BP | GO:0043647 | inositol phosphate metabolic process | 0.016056215 | 5 |
| BP | GO:1904427 | positive regulation of calcium ion transmembrane transport | 0.016056215 | 5 |
| BP | GO:0120163 | negative regulation of cold-induced thermogenesis | 0.016221383 | 4 |
| BP | GO:0032800 | receptor biosynthetic process | 0.016248099 | 3 |
| BP | GO:0060706 | cell differentiation involved in embryonic placenta development | 0.016248099 | 3 |
| BP | GO:1900120 | regulation of receptor binding | 0.016248099 | 3 |
| BP | GO:0001787 | natural killer cell proliferation | 0.017360232 | 2 |
| BP | GO:0010603 | regulation of cytoplasmic mRNA processing body assembly | 0.017360232 | 2 |
| BP | GO:0034135 | regulation of toll-like receptor 2 signaling pathway | 0.017360232 | 2 |
| BP | GO:0042536 | negative regulation of tumor necrosis factor biosynthetic process | 0.017360232 | 2 |
| BP | GO:0043379 | memory T cell differentiation | 0.017360232 | 2 |
| BP | GO:0070099 | regulation of chemokine-mediated signaling pathway | 0.017360232 | 2 |
| BP | GO:0071609 | chemokine (C-C motif) ligand 5 production | 0.017360232 | 2 |
| BP | GO:0090715 | immunological memory formation process | 0.017360232 | 2 |
| BP | GO:1900122 | positive regulation of receptor binding | 0.017360232 | 2 |
| BP | GO:2000109 | regulation of macrophage apoptotic process | 0.017360232 | 2 |
| BP | GO:2000317 | negative regulation of T-helper 17 type immune response | 0.017360232 | 2 |
| BP | GO:0002762 | negative regulation of myeloid leukocyte differentiation | 0.017413708 | 4 |
| BP | GO:0044764 | multi-organism cellular process | 0.017413708 | 4 |
| BP | GO:0072604 | interleukin-6 secretion | 0.017413708 | 4 |
| BP | GO:0007188 | adenylate cyclase-modulating G protein-coupled  receptor signaling pathway | 0.017552495 | 10 |
| BP | GO:0061844 | antimicrobial humoral immune response mediated  by antimicrobial peptide | 0.017916431 | 5 |
| BP | GO:0034446 | substrate adhesion-dependent cell spreading | 0.018024242 | 6 |
| BP | GO:0120034 | positive regulation of plasma membrane bounded  cell projection assembly | 0.018024242 | 6 |
| BP | GO:0002673 | regulation of acute inflammatory response | 0.018238665 | 8 |
| BP | GO:0008347 | glial cell migration | 0.018659295 | 4 |
| BP | GO:0014015 | positive regulation of gliogenesis | 0.018897707 | 5 |
| BP | GO:0032418 | lysosome localization | 0.018897707 | 5 |
| BP | GO:0043507 | positive regulation of JUN kinase activity | 0.018897707 | 5 |
| BP | GO:0046677 | response to antibiotic | 0.019785175 | 13 |
| BP | GO:0045761 | regulation of adenylate cyclase activity | 0.019859507 | 3 |
| BP | GO:1904994 | regulation of leukocyte adhesion to vascular endothelial cell | 0.019859507 | 3 |
| BP | GO:0043280 | positive regulation of cysteine-type endopeptidase activity  involved in apoptotic process | 0.020463065 | 7 |
| BP | GO:0007229 | integrin-mediated signaling pathway | 0.020542785 | 6 |
| BP | GO:0001894 | tissue homeostasis | 0.020720161 | 10 |
| BP | GO:0002730 | regulation of dendritic cell cytokine production | 0.020929612 | 2 |
| BP | GO:0032490 | detection of molecule of bacterial origin | 0.020929612 | 2 |
| BP | GO:0045628 | regulation of T-helper 2 cell differentiation | 0.020929612 | 2 |
| BP | GO:0045919 | positive regulation of cytolysis | 0.020929612 | 2 |
| BP | GO:0070673 | response to interleukin-18 | 0.020929612 | 2 |
| BP | GO:1903977 | positive regulation of glial cell migration | 0.020929612 | 2 |
| BP | GO:2000551 | regulation of T-helper 2 cell cytokine production | 0.020929612 | 2 |
| BP | GO:2001269 | positive regulation of cysteine-type endopeptidase activity  involved in apoptotic signaling pathway | 0.020929612 | 2 |
| BP | GO:0035821 | modification of morphology or physiology of other organism | 0.021519466 | 8 |
| BP | GO:0033032 | regulation of myeloid cell apoptotic process | 0.02181735 | 3 |
| BP | GO:0070102 | interleukin-6-mediated signaling pathway | 0.02181735 | 3 |
| BP | GO:0016311 | dephosphorylation | 0.022759069 | 17 |
| BP | GO:0000302 | response to reactive oxygen species | 0.023663098 | 10 |
| BP | GO:0051491 | positive regulation of filopodium assembly | 0.023876787 | 3 |
| BP | GO:0050732 | negative regulation of peptidyl-tyrosine phosphorylation | 0.024185757 | 4 |
| BP | GO:0030193 | regulation of blood coagulation | 0.024332953 | 5 |
| BP | GO:0051098 | regulation of binding | 0.024730228 | 14 |
| BP | GO:0002371 | dendritic cell cytokine production | 0.024774706 | 2 |
| BP | GO:0002674 | negative regulation of acute inflammatory response | 0.024774706 | 2 |
| BP | GO:0002862 | negative regulation of inflammatory response to antigenic stimulus | 0.024774706 | 2 |
| BP | GO:0002921 | negative regulation of humoral immune response | 0.024774706 | 2 |
| BP | GO:0006216 | cytidine catabolic process | 0.024774706 | 2 |
| BP | GO:0009972 | cytidine deamination | 0.024774706 | 2 |
| BP | GO:0016045 | detection of bacterium | 0.024774706 | 2 |
| BP | GO:0016554 | cytidine to uridine editing | 0.024774706 | 2 |
| BP | GO:0032252 | secretory granule localization | 0.024774706 | 2 |
| BP | GO:0036005 | response to macrophage colony-stimulating factor | 0.024774706 | 2 |
| BP | GO:0036006 | cellular response to macrophage colony-stimulating factor stimulus | 0.024774706 | 2 |
| BP | GO:0042368 | vitamin D biosynthetic process | 0.024774706 | 2 |
| BP | GO:0043922 | negative regulation by host of viral transcription | 0.024774706 | 2 |
| BP | GO:0046087 | cytidine metabolic process | 0.024774706 | 2 |
| BP | GO:0051132 | NK T cell activation | 0.024774706 | 2 |
| BP | GO:0070493 | thrombin-activated receptor signaling pathway | 0.024774706 | 2 |
| BP | GO:0071888 | macrophage apoptotic process | 0.024774706 | 2 |
| BP | GO:0097284 | hepatocyte apoptotic process | 0.024774706 | 2 |
| BP | GO:2001198 | regulation of dendritic cell differentiation | 0.024774706 | 2 |
| BP | GO:0038095 | Fc-epsilon receptor signaling pathway | 0.025197667 | 8 |
| BP | GO:0051100 | negative regulation of binding | 0.025197667 | 8 |
| BP | GO:1900046 | regulation of hemostasis | 0.025528509 | 5 |
| BP | GO:0002763 | positive regulation of myeloid leukocyte differentiation | 0.025705862 | 4 |
| BP | GO:0048871 | multicellular organismal homeostasis | 0.025709018 | 17 |
| BP | GO:0002675 | positive regulation of acute inflammatory response | 0.026037751 | 3 |
| BP | GO:0050849 | negative regulation of calcium-mediated signaling | 0.026037751 | 3 |
| BP | GO:0007189 | adenylate cyclase-activating G protein-coupled  receptor signaling pathway | 0.026246135 | 7 |
| CC | GO:0009897 | external side of plasma membrane | 1.64259E-32 | 58 |
| CC | GO:0001772 | immunological synapse | 4.44304E-12 | 12 |
| CC | GO:0098802 | plasma membrane receptor complex | 3.17034E-09 | 25 |
| CC | GO:0042613 | MHC class II protein complex | 5.2323E-07 | 6 |
| CC | GO:0042611 | MHC protein complex | 5.55589E-07 | 7 |
| CC | GO:0098636 | protein complex involved in cell adhesion | 5.29016E-06 | 7 |
| CC | GO:0045121 | membrane raft | 3.62738E-05 | 19 |
| CC | GO:0098857 | membrane microdomain | 3.78703E-05 | 19 |
| CC | GO:0098589 | membrane region | 6.25322E-05 | 19 |
| CC | GO:0042101 | T cell receptor complex | 7.05814E-05 | 11 |
| CC | GO:0001891 | phagocytic cup | 0.000209967 | 5 |
| CC | GO:0035579 | specific granule membrane | 0.000616265 | 8 |
| CC | GO:0042629 | mast cell granule | 0.001001421 | 4 |
| CC | GO:0010008 | endosome membrane | 0.001146802 | 21 |
| CC | GO:0098562 | cytoplasmic side of membrane | 0.001287878 | 11 |
| CC | GO:0009898 | cytoplasmic side of plasma membrane | 0.001461293 | 10 |
| CC | GO:0030667 | secretory granule membrane | 0.001525644 | 15 |
| CC | GO:0045335 | phagocytic vesicle | 0.001782862 | 9 |
| CC | GO:0042581 | specific granule | 0.001941881 | 10 |
| CC | GO:0001931 | uropod | 0.002185838 | 3 |
| CC | GO:0031254 | cell trailing edge | 0.002185838 | 3 |
| CC | GO:0005942 | phosphatidylinositol 3-kinase complex | 0.002213599 | 4 |
| CC | GO:0061702 | inflammasome complex | 0.002739342 | 3 |
| CC | GO:0030665 | clathrin-coated vesicle membrane | 0.002790715 | 8 |
| CC | GO:0008305 | integrin complex | 0.003717739 | 4 |
| CC | GO:0030479 | actin cortical patch | 0.004086393 | 3 |
| CC | GO:0061645 | endocytic patch | 0.004086393 | 3 |
| CC | GO:0030139 | endocytic vesicle | 0.004651055 | 14 |
| CC | GO:0031904 | endosome lumen | 0.005218212 | 4 |
| CC | GO:0030136 | clathrin-coated vesicle | 0.006120031 | 10 |
| CC | GO:0070820 | tertiary granule | 0.007472929 | 9 |
| CC | GO:0005765 | lysosomal membrane | 0.007547619 | 15 |
| CC | GO:0098852 | lytic vacuole membrane | 0.007735845 | 15 |
| CC | GO:0036019 | endolysosome | 0.007822721 | 3 |
| CC | GO:0030666 | endocytic vesicle membrane | 0.008370968 | 9 |
| CC | GO:0045334 | clathrin-coated endocytic vesicle | 0.009938138 | 5 |
| MF | GO:0004896 | cytokine receptor activity | 1.71119E-15 | 21 |
| MF | GO:0015026 | coreceptor activity | 6.84777E-12 | 13 |
| MF | GO:0001614 | purinergic nucleotide receptor activity | 1.55098E-10 | 9 |
| MF | GO:0016502 | nucleotide receptor activity | 1.55098E-10 | 9 |
| MF | GO:0019955 | cytokine binding | 4.395E-10 | 18 |
| MF | GO:0035586 | purinergic receptor activity | 1.71052E-09 | 9 |
| MF | GO:0001637 | G protein-coupled chemoattractant receptor activity | 2.56536E-09 | 9 |
| MF | GO:0004950 | chemokine receptor activity | 2.56536E-09 | 9 |
| MF | GO:0001608 | G protein-coupled nucleotide receptor activity | 3.72889E-09 | 7 |
| MF | GO:0045028 | G protein-coupled purinergic nucleotide receptor activity | 3.72889E-09 | 7 |
| MF | GO:0005126 | cytokine receptor binding | 5.46104E-09 | 25 |
| MF | GO:0016493 | C-C chemokine receptor activity | 1.93935E-08 | 8 |
| MF | GO:0019956 | chemokine binding | 2.04779E-08 | 9 |
| MF | GO:0019957 | C-C chemokine binding | 2.8532E-08 | 8 |
| MF | GO:0005125 | cytokine activity | 9.93793E-08 | 20 |
| MF | GO:0023023 | MHC protein complex binding | 8.30432E-07 | 7 |
| MF | GO:0032395 | MHC class II receptor activity | 1.18053E-06 | 5 |
| MF | GO:0042379 | chemokine receptor binding | 1.73138E-06 | 10 |
| MF | GO:0005164 | tumor necrosis factor receptor binding | 4.05157E-06 | 7 |
| MF | GO:0004715 | non-membrane spanning protein tyrosine kinase activity | 6.61668E-06 | 8 |
| MF | GO:0008009 | chemokine activity | 1.07934E-05 | 8 |
| MF | GO:0023026 | MHC class II protein complex binding | 1.83367E-05 | 5 |
| MF | GO:0005085 | guanyl-nucleotide exchange factor activity | 2.31134E-05 | 16 |
| MF | GO:0048020 | CCR chemokine receptor binding | 3.95179E-05 | 7 |
| MF | GO:0030695 | GTPase regulator activity | 4.96956E-05 | 19 |
| MF | GO:0032813 | tumor necrosis factor receptor superfamily binding | 6.20072E-05 | 7 |
| MF | GO:0035591 | signaling adaptor activity | 6.64789E-05 | 9 |
| MF | GO:0045236 | CXCR chemokine receptor binding | 6.8475E-05 | 4 |
| MF | GO:0042169 | SH2 domain binding | 0.000124394 | 6 |
| MF | GO:0005096 | GTPase activator activity | 0.000128451 | 17 |
| MF | GO:0005070 | SH3/SH2 adaptor activity | 0.000138329 | 7 |
| MF | GO:0019865 | immunoglobulin binding | 0.000154234 | 5 |
| MF | GO:1990782 | protein tyrosine kinase binding | 0.000214377 | 9 |
| MF | GO:0001618 | virus receptor activity | 0.000223162 | 8 |
| MF | GO:0104005 | hijacked molecular function | 0.000223162 | 8 |
| MF | GO:0042287 | MHC protein binding | 0.000227552 | 6 |
| MF | GO:0060589 | nucleoside-triphosphatase regulator activity | 0.000249455 | 19 |
| MF | GO:0043028 | cysteine-type endopeptidase regulator activity  involved in apoptotic process | 0.00029964 | 6 |
| MF | GO:0008528 | G protein-coupled peptide receptor activity | 0.000405603 | 11 |
| MF | GO:0001653 | peptide receptor activity | 0.000570574 | 11 |
| MF | GO:0017048 | Rho GTPase binding | 0.000585282 | 12 |
| MF | GO:0042288 | MHC class I protein binding | 0.000858727 | 4 |
| MF | GO:0005088 | Ras guanyl-nucleotide exchange factor activity | 0.000941074 | 10 |
| MF | GO:0048018 | receptor ligand activity | 0.001127444 | 22 |
| MF | GO:0001784 | phosphotyrosine residue binding | 0.001787383 | 5 |
| MF | GO:0005089 | Rho guanyl-nucleotide exchange factor activity | 0.001801734 | 7 |
| MF | GO:0019992 | diacylglycerol binding | 0.002029801 | 3 |
| MF | GO:0061578 | Lys63-specific deubiquitinase activity | 0.002029801 | 3 |
| MF | GO:0043027 | cysteine-type endopeptidase inhibitor activity  involved in apoptotic process | 0.00205504 | 4 |
| MF | GO:0004435 | phosphatidylinositol phospholipase C activity | 0.002386833 | 4 |
| MF | GO:0017124 | SH3 domain binding | 0.002406926 | 9 |
| MF | GO:0005068 | transmembrane receptor protein tyrosine kinase adaptor activity | 0.002595769 | 3 |
| MF | GO:0030246 | carbohydrate binding | 0.002931347 | 14 |
| MF | GO:0003823 | antigen binding | 0.002990732 | 10 |
| MF | GO:0004629 | phospholipase C activity | 0.003157363 | 4 |
| MF | GO:0043325 | phosphatidylinositol-3,4-bisphosphate binding | 0.004082009 | 4 |
| MF | GO:0043548 | phosphatidylinositol 3-kinase binding | 0.004082009 | 4 |
| MF | GO:0005525 | GTP binding | 0.004155257 | 17 |
| MF | GO:0030674 | protein binding, bridging | 0.004600538 | 10 |
| MF | GO:0032550 | purine ribonucleoside binding | 0.004617139 | 17 |
| MF | GO:0004697 | protein kinase C activity | 0.004838869 | 3 |
| MF | GO:0001883 | purine nucleoside binding | 0.004990517 | 17 |
| MF | GO:0032549 | ribonucleoside binding | 0.005120333 | 17 |
| MF | GO:0045309 | protein phosphorylated amino acid binding | 0.005236917 | 5 |
| MF | GO:0001882 | nucleoside binding | 0.006108408 | 17 |
| MF | GO:0019001 | guanyl nucleotide binding | 0.006904908 | 17 |
| MF | GO:0032561 | guanyl ribonucleotide binding | 0.006904908 | 17 |
| MF | GO:0019902 | phosphatase binding | 0.008212379 | 10 |
| MF | GO:0004869 | cysteine-type endopeptidase inhibitor activity | 0.00901441 | 5 |
| MF | GO:0008329 | signaling pattern recognition receptor activity | 0.009228181 | 3 |
| MF | GO:1901981 | phosphatidylinositol phosphate binding | 0.009649819 | 9 |
| MF | GO:0004713 | protein tyrosine kinase activity | 0.009876967 | 8 |
| MF | GO:0038187 | pattern recognition receptor activity | 0.010592472 | 3 |
| MF | GO:0019903 | protein phosphatase binding | 0.012644169 | 8 |
| MF | GO:0005543 | phospholipid binding | 0.014500688 | 17 |
